# Panchromatic Photocatalysis using a Ruthenium Polypyridyl Complex

**DOI:** 10.1021/jacs.6c09162

**Published:** 2026-07-27

**Authors:** Minling Zhong, Joohyun Lee, Jie Huang, Jeanette A. Krause, Kaiqi Long, Claudia Turro, Christopher G. Elles, Yujie Sun

**Affiliations:** † Department of Chemistry, 2514 University of Cincinnati, Cincinnati, Ohio 45221, United States; ‡ Department of Chemistry, 4202 University of Kansas, Lawrence, Kansas 66045, United States; § Department of Chemistry and Biochemistry, 2647 The Ohio State University, Columbus, Ohio 43210, United States; ∥ Brigham and Women’s Hospital, Harvard Medical School, Boston, Massachusetts 02115, United States

## Abstract

Photocatalysis has reshaped synthetic chemistry and materials science by enabling various transformations under mild conditions using light as a traceless reagent. However, most molecular photocatalysts/photosensitizers only operate within a narrow blue-to-green window, which imposes practical limitations, such as shallow light penetration, competing absorption by substrates and additives, and incompatibility with light-sensitive functionalities. Extending the activity of photocatalysis into the deep-red and near-infrared (NIR) regions offers a compelling solution. However, the lower energy of red and NIR photons typically leads to weakly reactive or short-lived excited states, limiting their photocatalytic capability. A general strategy that enables a single molecular chromophore to function across a broad spectral range remains elusive. Herein we report a molecular engineering strategy that renders multiple excitation pathways generating the same reactive excited state within one ruthenium complex. By incorporating extended π-conjugation into a ruthenium polypyridyl framework, the complex can be activated through one-photon excitation in the short-wavelength visible region, weak direct excitation of the lowest metal-to-ligand charge transfer manifold in the deep red, and two-photon absorption under NIR light irradiation up to 850 nm. Spectroscopic and kinetic analyses demonstrate that distinct wavelength-dependent excitation modes populate a common long-lived MLCT excited state, preserving photocatalytic performance across the UV–vis–NIR spectral window. This wavelength-adaptive behavior enables diverse organic transformations and polymerization processes under conditions inaccessible to conventional photocatalysts. More broadly, this work illustrates how rational ligand design can make multiple excitation modes converge on one reactive state, offering a practical route to ruthenium photocatalysts that operate across the visible-to-NIR range.

## Graphical abstract

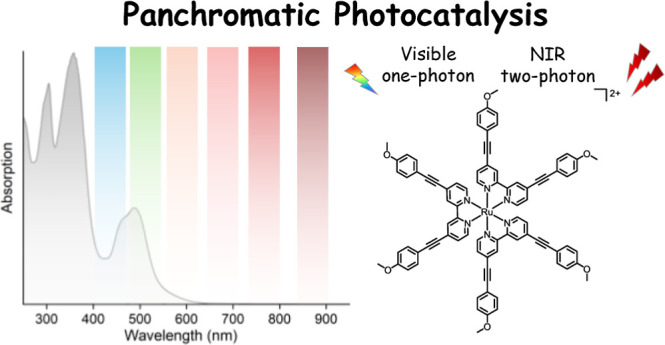

## Introduction

Photocatalysis has emerged as a versatile strategy for driving a large number of chemical transformations under ambient conditions.^1–3^ Most molecular photocatalysts/photosensitizers, including transition metal complexes and organic chromophores, rely on ultraviolet (UV) or short-wavelength visible light to access excited states with energies of 50–80 kcal mol^–1^ (Figure 1a).^4–8^ Despite their widespread application, these conventional one-photon-absorbing chromophores usually suffer from several intrinsic drawbacks, including shallow penetration depth through reaction media (especially in biological environments),^9,10^ competing light absorption by substrates and/or cocatalysts,^11^ poor compatibility with light-sensitive functional groups,^12^ and limited utilization of the solar spectrum. These constraints may ultimately restrict reaction scalability, selectivity, and energy utilization.^13^ Against this backdrop, near-infrared (NIR) light represents an attractive alternative excitation source because of its deeper penetration through reaction media and reduced absorption by most substrates.^14–16^ However, the practical implementation of NIR photocatalysis is difficult. According to the energy-gap law, chromophores that absorb light in the NIR region typically generate low-energy, short-lived excited states, which limit their ability to drive demanding chemical transformations. As a result, the efficient utilization of low-energy photons to generate chemically useful excited states of reactive chromophores remains challenging in conventional photocatalysis.

**Figure 1:**
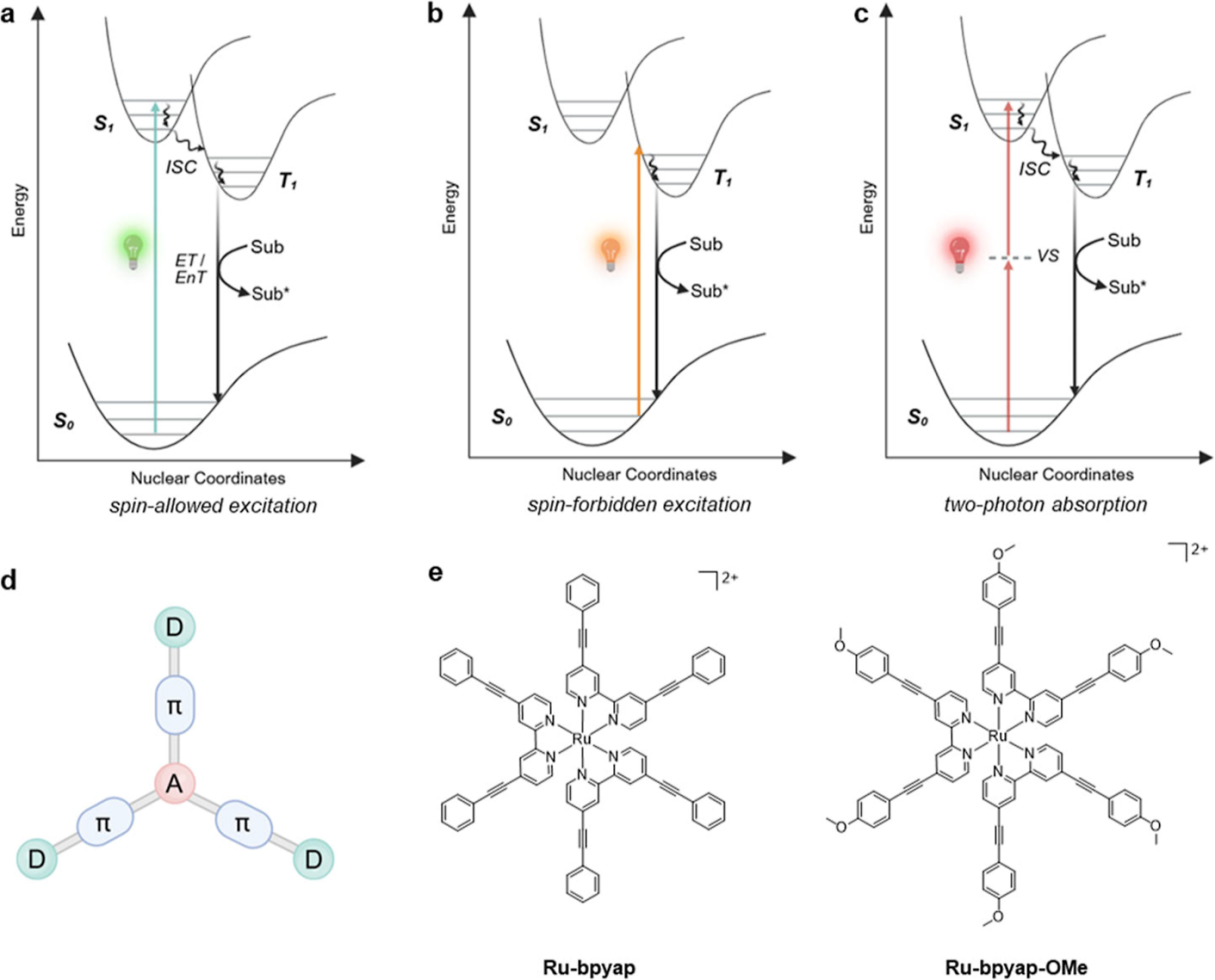
(a–c) Schematic illustration of photocatalysis enabled by (a) spin-allowed excitation, (b) direct excitation of the lowest (predominantly triplet) MLCT manifold, and (c) two-photon absorption. (d) Design strategies of TPA photocatalysts. (e) Molecular structures of **Ru-bpyap** and **Ru-bpyap-OMe**.

Several strategies have been developed to extend the applicable wavelength of the excitation light into the deep-red and NIR regions. For instance, incorporation of third–row transition metal, such as osmium,^17–20^ enhances spin–orbit coupling and enables direct spin-forbidden excitations into the desirable triplet excited states, circumventing the energy-wasting intersystem crossing process (Figure 1b).^21–23^ Although this approach extends the absorption to the red region, the resulting excited states often possess low energy and hence limited photocatalytic capability. Alternatively, multiphoton excitation strategies,^24–26^ such as triplet–triplet annihilation upconversion (TTA-UC),^27,28^ combine the energies of multiple low-energy photons to produce higher-energy excited states. Recent studies have demonstrated that NIR light-absorbing sensitizers can activate suitable annihilators to enable NIR light-driven photocatalysis.^29–31^ However, the efficiency of TTA-UC critically depends on the precise energetic matching and efficient interaction between sensitizer and annihilator,^32,33^ typically requiring an intermolecular distance lower than 10 Å to ensure effective energy transfer.^34^ These stringent requirements significantly increase system complexity and are further constrained by the limited availability of suitable sensitizer–annihilator pairs, thereby restricting general applicability.

Two-photon absorption (TPA)^35^ provides a conceptually simpler route to NIR photocatalysis, in which a single chromophore simultaneously absorbs two low-energy photons to reach a high-energy excited state (Figure 1c).^36^ This nonlinear optical process has been widely exploited in bioimaging,^37–39^ 3D microfabrication,^40,41^ and photodynamic therapy,^42–46^ yet its application in photocatalysis remains comparatively underdeveloped,^47,48^ largely because most conventional photocatalysts exhibit very small TPA cross sections (σ_2_). For example, the widely used [Ru­(bpy)_3_]^2+^ photosensitizer displays a σ_2_ value of only 4.3 GM at 880 nm (1 GM = 10^–50^ cm^4^ s photon^–1^),^49^ which severely limits its photocatalytic activity under NIR light excitation. Therefore, recent efforts have focused on the rational molecular design to enhance the TPA cross sections of new photocatalysts and sensitizers.^50,51^ Through the construction of octupolar donor-π-acceptor architectures (Figure 1d), strong intramolecular charge transfer and large molecular hyperpolarizability have been identified as key factors for achieving high σ_2_ values.^52^ Such a design can produce chromophores with substantially improved nonlinear optical response, enabling two-photon excitation even under inexpensive, incoherent light sources that exhibit photon-bunching behavior.^53^ Nevertheless, achieving panchromatic photocatalytic activity (i.e., applicable from UV–vis to NIR light excitation) within a single molecular platform remains an outstanding challenge.

Here we report a strategy to achieve panchromatic photocatalysis that integrates spin-allowed excitation, spin-forbidden excitation, and two-photon absorption within a single ruthenium polypyridyl complex **Ru-bpyap-OMe** (Figure 1e). Throughout this work, the terms “spin-allowed” and “spin-forbidden” are used in an operational sense, to distinguish direct optical population of the higher-lying ^1^MLCT manifold from the much weaker direct population of the lowest (predominantly triplet) MLCT manifold. We note that, owing to the strong spin–orbit coupling at the ruthenium center, these MLCT states are extensively spin-mixed and do not behave as the pure singlet/triplet states in organic chromophores; the spin labels are therefore approximate descriptors of transition probability rather than strict selection rules.^54–58^ By incorporating rigid, conjugated phenylethynyl-substituted bipyridine ligands, the resulting **Ru-bpyap-OMe** possesses enhanced π-conjugation, reduced nonradiative decay (τ > 1.5 μs), and a large TPA cross-section profile in the NIR region (approaching 2400 GM at ∼ 700 nm). **Ru-bpyap-OMe** exhibits photocatalytic activity across a broad spectral window (450–850 nm), wherein its reactive excited state is populated through distinct excitation pathways depending on the irradiation wavelength. By correlating spectroscopic features with photocatalytic performance, we demonstrate how wavelength-dependent excitation modes govern catalytic reactivity. This work illustrates how rational ligand design can bring multiple excitation modes to converge on a common reactive state, offering a practical route to ruthenium photocatalysts that operate across the visible-to-NIR range. Beyond the breadth of accessible wavelengths and the range of transformations it supports, a particular advantage is the large two-photon absorption cross-section of **Ru-bpyap-OMe**, which allows even its near-infrared reactivity to be driven by inexpensive, commercially available NIR LEDs rather than pulsed laser sources, helping to make this broad operational window practically accessible.

## Results and Discussion

### Synthesis, Characterization, and Theoretical Calculation

During the development of competent TPA chromophores, it was recognized that the rigid and cylindrically symmetric acetylene bridges can preserve π-conjugation across a broad range of rotational conformations, thereby minimizing rotation-induced energy dissipation and facilitating efficient charge delocalization.^59^ On this basis, we designed and synthesized a new class of Ru­(II) complexes bearing 4,4′-di­(phenylethynyl)-2,2′-bipyridine (bpyap) ligands and their derivatives (Figure 1e). The parent ligand bpyap and its *para*-methoxy-substituted analog bpyap-OMe were prepared via Sonogashira coupling of 4,4′-dibromo-2,2′-bipyridine with the corresponding phenylacetylene derivatives (see the Supporting Information for details). Depending on the solvent employed, three polymorphic forms of bpyap-OMe were obtained. Single-crystal X-ray diffraction analysis of the three polymorphs (Figure S1) reveals a nearly coplanar arrangement of the two pyridyl units, with a torsion angle of ∠N1–C12–C14–N2 = 168–180°, indicative of an extended and rigid conjugated backbone. The C7–C8 and C19–C20 bond lengths in the 1.196–1.200 Å range are characteristic of a C≡C triple bond and comparable to that of diphenylacetylene (1.198 Å),^60^ confirming the structural integrity of the acetylene linker. This rigid, conjugated architecture is expected to enhance intramolecular charge transfer and molecular hyperpolarizability, both critical parameters for achieving large TPA cross sections. Complexation was achieved by refluxing Ru­(DMSO)_4_Cl_2_ with 3.5 equiv of the corresponding ligand in ethanol, followed by anion exchange with excess NH_4_PF_6_. This procedure afforded the homoleptic complexes **Ru-bpyap** and **Ru-bpyap-OMe** in good yields. All complexes were fully characterized by NMR spectroscopy and mass spectrometry as detailed in Supporting Information.

The photophysical and electrochemical properties of these Ru complexes were systematically examined, using [Ru­(bpy)_3_]^2+^ (**Ru-bpy**) as a control sample. As shown in Figure 2a, all complexes display comparable spectral profiles in acetonitrile, with intense high-energy absorption bands in the 200–400 nm range assigned to intraligand ππ* transitions. In the visible region, the classical singlet MLCT bands are observed at 451 nm for **Ru-bpy** (ε = 13,852 M^–1^ cm^–1^), 484 nm for **Ru-bpyap** (ε = 44,737 M^–1^ cm^–1^), and 487 nm for **Ru-bpyap-OMe** (ε = 45,712 M^–1^ cm^–1^). The pronounced bathochromic shift and more than 3-fold increase in molar absorptivity from **Ru-bpy** to **Ru-bpyap** and **Ru-bpyap-OMe** reflect the enhanced π-conjugation and increased donor strength of the bpyap-type ligands, which collectively strengthen their ^1^MLCT absorption. At ambient temperature, **Ru-bpy** exhibits emission centered at 607 nm, whereas **Ru-bpyap** and **Ru-bpyap-OMe** display red-shifted phosphorescence maxima at 636 nm (Figure 2a), consistent with the stabilization of the ligand-centered π* orbitals. It should be noted that the excitation spectra closely mirror their corresponding absorption spectra (Figures S6 and S7), confirming that the observed emission originates from the target ruthenium complexes, instead of highly emissive impurities. Furthermore, time-resolved measurements in deaerated acetonitrile reveal excited-state lifetimes of 1.66 μs for **Ru-bpyap** and 1.48 μs for **Ru-bpyap-OMe**, both substantially longer than that of **Ru-bpy** (0.95 μs), under similar experimental conditions.^61^ Such a lifetime enhancement is consistent with increased structural rigidity and reduced nonradiative relaxation arising from the extended conjugated framework, features that are advantageous for photocatalytic applications requiring long-lived excited states.

**Figure 2:**
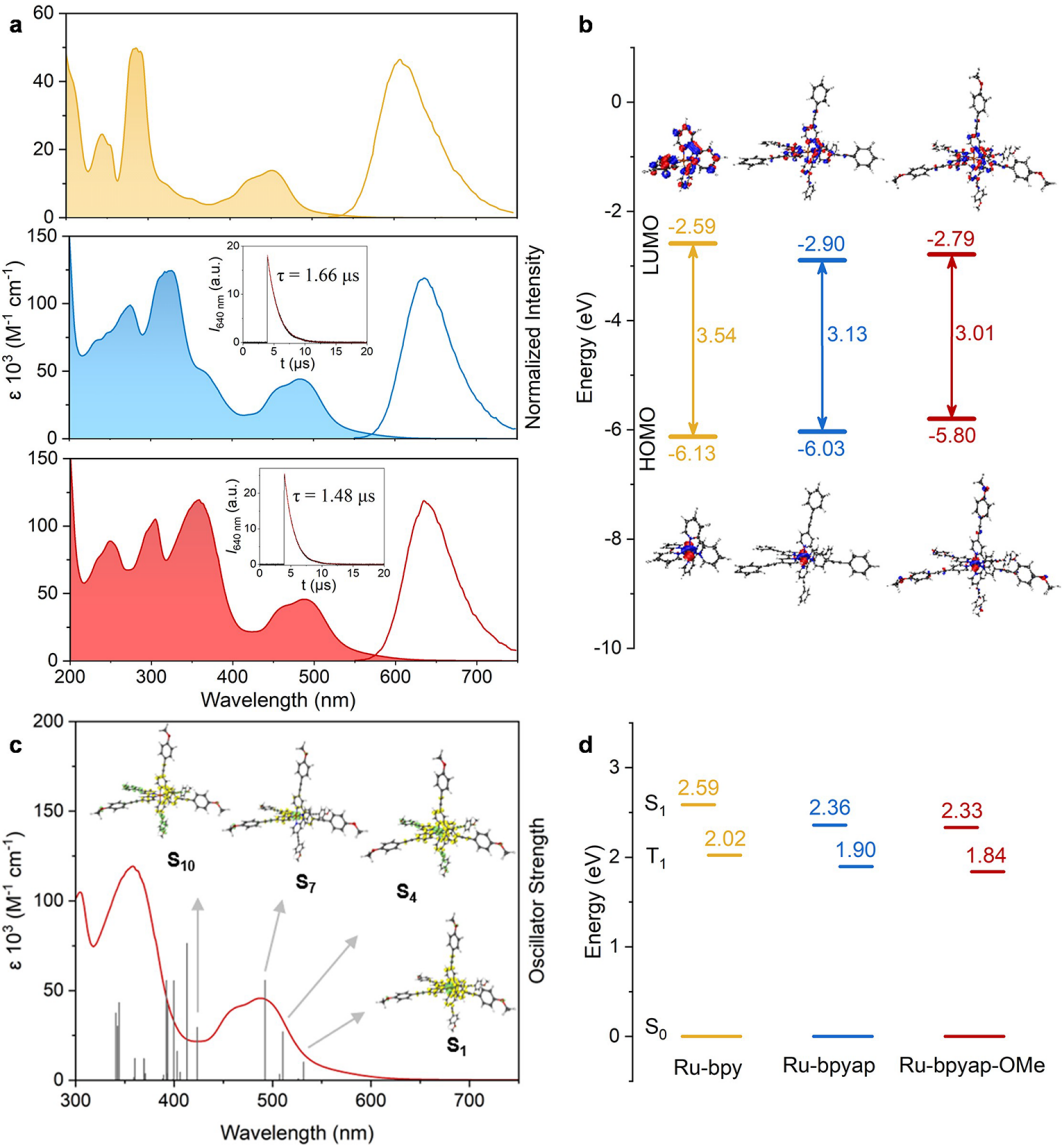
(a) Absorption (filled area) and normalized emission (line) spectra of **Ru-bpy**, **Ru-bpyap**, and **Ru-bpyap-OMe** in deaerated acetonitrile. Insets show the decay at their corresponding emission maxima. (b) Calculated frontier orbitals and corresponding energies of the three ruthenium complexes. (c) Comparison between the experimental absorption spectrum and the calculated singlet excited states of **Ru-bpyap-OMe** with the EDDMs of S_1_, S_4_, S_7_, and S_10_ states (isovalue = 0.04). Green and yellow indicate electron density decrease and increase, respectively. (d) Calculated relative energies of the S_0_, S_1_, and T_1_ states of the three ruthenium complexes.

Cyclic voltammetry further highlights the impact of ligand modification on the redox properties of the complexes (Figure S8). **Ru-bpyap** and **Ru-bpyap-OMe** exhibit a quasi-reversible Ru^III/II^ oxidation feature between +0.87 and +0.83 V vs Fc^+/0^ (all potentials reported herein are referenced to Fc^+/0^ unless otherwise noted), slightly cathodically shifted relative to **Ru-bpy** (+0.90 V).^55^ This negative shift reflects the stronger electron-donating character of the bpyap-type ligands, which destabilizes the Ru-centered highest occupied molecular orbital (HOMO) and facilitates oxidation. In addition, three reversible reduction processes are observed between −1.46 and −1.86 V, with the first reduction occurring at more positive potentials than in **Ru-bpy** (−1.70 V).^55^ This anodic shift indicates stabilization of the ligand π* orbitals due to extended conjugation, thereby narrowing the HOMO–LUMO (LUMO = lowest unoccupied molecular orbital) gap and rationalizing the observed red-shifted absorption and emission maxima.

Density functional theory (DFT) calculations provide further insight into the electronic structures of these complexes (Figure 2b). The calculated HOMOs are predominantly Ru 4d in character, with energies of −6.13, −6.03, and −5.80 eV for **Ru-bpy**, **Ru-bpyap**, and **Ru-bpyap-OMe**, respectively. The progressively increasing energy of the HOMOs among these complexes, particularly in **Ru-bpyap-OMe** with a methoxy-substituted bpyap ligand, is consistent with the enhanced electron-donating capability of the substituent. In contrast, the LUMOs are primarily ligand-centered, and the π-extension in bpyap-type ligands lowers the energy of this orbital through delocalization over the phenylethynyl framework. Time-dependent DFT (TD-DFT) calculations confirm that the lowest-energy singlet states possess dominant ^1^MLCT character. For **Ru-bpyap-OMe**, the calculated S_1_ state appears at 531 nm (*f* = 0.10), while the prominent S_7_ state at 492 nm (*f* = 0.55) corresponds closely to the experimentally observed ^1^MLCT absorption band (Figure 2c). Electron density difference maps (EDDMs) of representative singlet excited states (e.g., S_4_ and S_10_) reveal mixed MLCT and ILCT contributions, indicating substantial delocalization of the photoexcited electron density across the Ru center and the extended ligand scaffold. Furthermore, the calculated T_1_ energies decrease progressively from **Ru-bpy** to **Ru-bpyap** and **Ru-bpyap-OMe** (Figure 2d), in agreement with the experimentally observed bathochromic shift in the phosphorescence (Figure 2a).

To elucidate the excited-state dynamics underpinning the panchromatic response, both femtosecond transient absorption (fsTA) and nanosecond transient absorption (nsTA) spectroscopies were performed using 480 nm excitation. For **Ru-bpyap** in acetonitrile, the fsTA spectra display excited-state absorption features at 370–430 nm and beyond 530 nm, as well as a ground-state bleach between 470 and 530 nm. The transient absorption signal centered near 480 nm decays monoexponentially, resulting in a lifetime of 1.71 μs (Figure S9), consistent with the lifetime of the MLCT emission (Figure 2a). **Ru-bpyap-OMe** exhibits similar yet spectrally broadened features, with a ground-state bleach spanning 435–560 nm and excited-state absorption bands at 405 nm and beyond 560 nm (Figure 3a). Kinetic analysis at 405 nm yields an excited-state lifetime of 1.54 μs (Figure S10), in excellent agreement with the decay of the emission (Figure 2a). Strikingly, excitation at 800 nm (where no measurable one-photon absorption is observed) produces transient absorption spectra that are nearly identical to those obtained under 480 nm excitation (Figure 3a). The spectral similarities of the transient absorption results under both excitation conditions demonstrates that the same excited state is populated, providing direct spectroscopic evidence that the reactive MLCT of **Ru-bpyap-OMe** can be accessed via a two-photon absorption pathway under near-infrared light irradiation.

**Figure 3:**
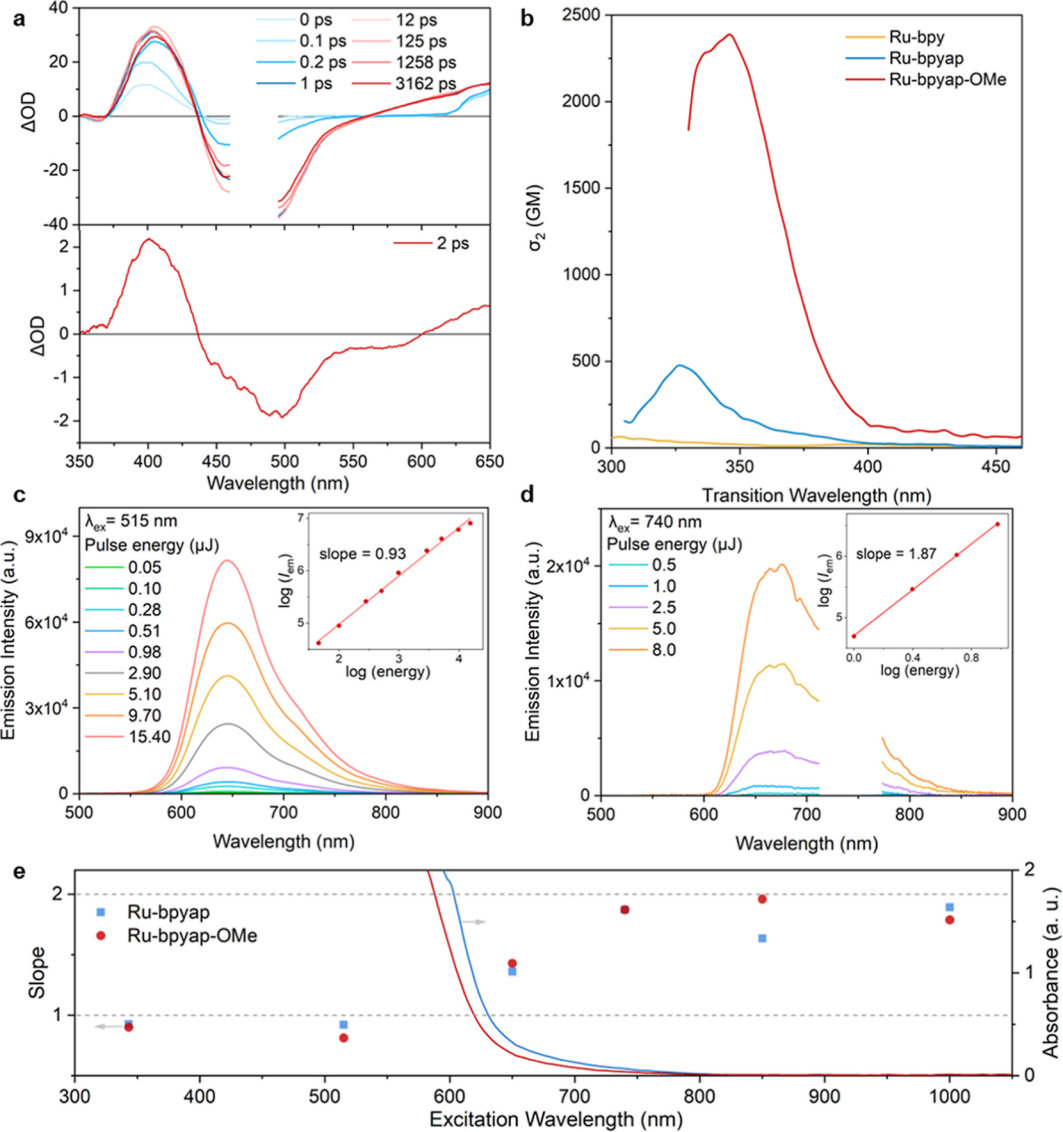
(a) Femtosecond transient absorption spectra of **Ru-bpyap-OMe** in deaerated acetonitrile under excitation at 480 nm (3 μJ) (top) and 800 nm (60 μJ) (bottom) at different delay times. (b) Two-photon absorption profiles of **Ru-bpy**, **Ru-bpyap**, and **Ru-bpyap-OMe** measured in deaerated acetonitrile. (c-d) Power-dependent emission spectra of **Ru-bpyap-OMe** upon excitation at 515 nm (c) and 740 nm (d). Insets show the linear fits of log­(emission intensity) versus log­(laser pulse energy). The pump-scattering region around 740 nm was removed in (d) for clarity. (e) Slopes of the logarithmic plots of emission intensity against laser pulse energy at various excitation wavelengths, together with the absorption spectra of **Ru-bpyap** and **Ru-bpyap-OMe** (10 mM in acetonitrile, corresponding to the concentration used for TPA emission measurements) showing the direct excitation region of the lowest MLCT manifold.

To quantitatively assess the nonlinear optical response of these ruthenium complexes, broadband two-photon absorption spectra were collected using a pump–probe method to determine their TPA profiles (Figure 3b), wherein the energy of the reported transition wavelength is equal to the combined energies of both pump and probe photons.^62^ As shown in Figure 3b, **Ru-bpy** exhibits modest TPA cross section (σ_2_) values ranging from 9 to 65 GM over the transition wavelength range of 300–440 nm, consistent with reported results.^49^ In contrast, the incorporation of the π-extended bpyap ligand markedly enhances the TPA response, with **Ru-bpyap** reaching σ_2_ of 477 GM at 327 nm. Further introduction of the electron-donating methoxy substituent dramatically amplifies the two-photon absorption, affording a maximum σ_2_ approaching 2400 GM at 346 nm for **Ru-bpyap-OMe**. Notably, significant σ_2_ values persist beyond 450 nm (corresponding to excitation wavelengths >900 nm). Taken together, these results indicate that **Ru-bpyap-OMe** exhibits efficient two-photon absorption across the 700–900 nm NIR region.

Because one-photon absorption (OPA) and TPA show distinct dependencies on excitation light intensity, power-dependence emission measurements were also conducted to further verify the excitation mechanism. OPA depends linearly on excitation intensity, whereas TPA has a quadratic dependence. The phosphorescence spectra of **Ru-bpyap-OMe** were collected under laser excitation spanning 343–1000 nm with varying power densities, and the logarithmic relationship between observed emission intensity and utilized laser pulse energy were plotted (Figures S11 and S12). At shorter excitation wavelengths such as 515 nm, the slope of the logarithmic plot is approximately 1 (0.93 in Figure 3c inset), consistent with a single-photon absorption mechanism. In contrast, excitation at longer wavelengths, such as 740 nm, yields a slope approaching 2 (1.87 in Figure 3d inset), indicative of a dominant two-photon excitation pathway. Across the full spectral range (343–1000 nm), the extracted slopes increase progressively from 0.92 to 1.96 (Figure 3e). Notably, excitation near the nominal 0–0 transition band of the reactive MLCT state (∼650 nm) produces an intermediate slope of ∼1.36, suggesting the coexistence of spin-forbidden single-photon excitation (in the operational sense defined above) and two-photon absorption in this regime. Collectively, the above photophysical and spectroscopic studies show that **Ru-bpyap** and **Ru-bpyap-OMe** share the same qualitative behavior, with the methoxy substitutions amplifying TPA features significantly. **Ru-bpyap-OMe** was therefore selected as the optimized photocatalyst for the wavelength-dependent studies described in the following section.

### Photocatalytic Performance as a Function of Irradiation Wavelength

To establish a direct relationship between the excitation pathway and panchromatic photocatalytic performance of **Ru-bpyap-OMe**, we first employed the hydrodehalogenation of 2-bromo-4′-fluoroacetophenone as a model reaction (Figure 4a). Experiments were conducted in deaerated acetonitrile using triethanolamine (TEOA) as a sacrificial electron/proton donor. Irradiation was performed with LEDs centered at 525, 660, and 740 nm, and the product was quantified by ^19^F NMR as a function of photolysis time (Figure 4b–d). By varying the excitation light intensity and analyzing the initial linear region of the conversion profiles (Figure S21), reaction rates were extracted and correlated with photon flux using logarithmic analysis (insets in Figure 4b–d). As shown in Figure 4b, under 525 nm irradiation (12 mW cm^–2^), complete conversion was achieved within 21 min using only 0.01 mol % **Ru-bpyap-OMe**. The reaction proceeds rapidly at low catalyst loading and moderate light intensity, consistent with the efficient population of its MLCT state through spin-allowed one-photon absorption and high molar absorptivity in this spectral region (Figure 2a). The initial reaction rate displays a linear dependence on photon power, confirming that the photocatalytic process at this wavelength is governed by the OPA mechanism. When the excitation wavelength was extended to 660 nm, different catalytic behavior emerged. Even with a higher photon power of 300 mW cm^–2^, full conversion required 2 mol % photocatalyst loading and 70 min of irradiation. The reduced efficiency is consistent with the significantly lower absorption coefficient at this wavelength and the involvement of spin-forbidden excitation. Despite diminished absorptivity, the reaction rate retains a linear dependence on photon power (slope = 1.01 in Figure 4c inset), indicating that the transformation remains dominated by a one-photon process. At 740 nm, where no appreciable one-photon absorption is detected, the reaction enters a distinct excitation regime. Under irradiation at 200–800 mW cm^–2^ using 10 mol % **Ru-bpyap-OMe**, the reaction rate exhibits a near-quadratic dependence (slope = 1.79) on photon power (Figure 4d inset), providing unambiguous evidence of a TPA-driven photocatalytic mechanism. This quadratic behavior aligns with the excitation power dependence of photoluminescence analysis (Figure 3e). Such a higher catalyst loading required at 740 nm reflects the intrinsically lower excitation probability associated with nonlinear optical processes. Nevertheless, when the amount of **Ru-bpyap-OMe** was reduced to 2 mol % and the reaction time extended to 15 h, complete conversion was still achieved under 740 nm irradiation (Figure S44 and S45), demonstrating that TPA-driven photocatalysis is both mechanistically operative and synthetically viable.

**Figure 4:**
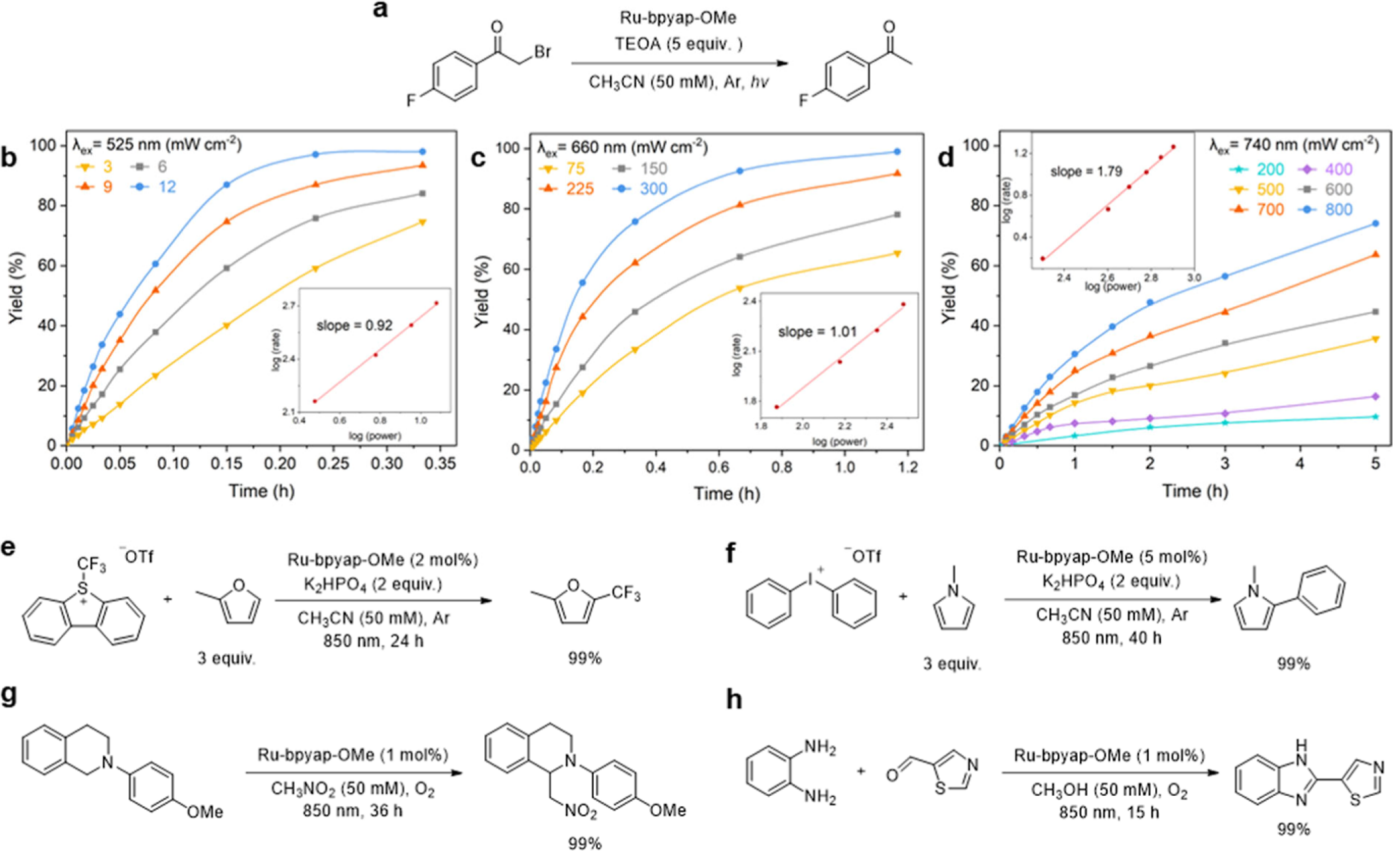
(a) Photocatalytic hydrodehalogenation of 2-bromo-4′-fluoroacetophenone using **Ru-bpyap-OMe** as the photocatalyst upon irradiation at different wavelengths. (b-d) Time-dependent product yields under irradiation with 525 nm (b), 660 nm (c), and 740 nm (d) LEDs of different power. Reaction conditions: 50 mM 2-bromo-4′-fluoroacetophenone, **Ru-bpyap-OMe** (0.01, 2.0, and 10 mol % for 525, 660, and 740 nm irradiation, respectively), 4 mL acetonitrile, argon atmosphere, room temperature. Insets show the linear fits of log­(reaction rate) versus log­(power density). (e-h) Four representative organic transformations photocatalyzed by **Ru-bpyap-OMe** under 850 nm irradiation: (e) trifluoromethylation of 2-methylfuran, (f) phenylation of 1-methyl-1*H*-pyrrole, (g) cross-dehydrogenative coupling between 2-(4-methoxyphenyl)-1,2,3,4-tetrahydroisoquinoline and nitromethane, and (h) cyclocondensation between *o*-phenylenediamine and 5-thiazolecarboxaldehyde.

To further assess the photocatalytic performance of **Ru-bpyap-OMe** to operate under NIR light excitation, we extended the irradiation wavelength to 850 nm. Although a calibrated LED source with precise intensity control as the above LEDs was not available, commercially accessible 850 nm LEDs were employed to perform the following four reactions using **Ru-bpyap-OMe** as the sole photocatalyst. As presented in Figure 4e, trifluoromethylation of 2-methylfuran using a dibenzothiophenium triflate reagent proceeded to complete conversion after 24 h of 850 nm irradiation with 2 mol % **Ru-bpyap-OMe**. Product formation was confirmed by both ^1^H and ^19^F NMR spectroscopies and high-resolution mass spectrometry (Figures S46, S47, and S53). The successful generation of trifluoromethyl radicals under 850 nm excitation highlights the substantial excited-state reducing power of **Ru-bpyap-OMe** accessible through TPA. Next, a redox-neutral phenylation reaction was evaluated under an argon atmosphere. In this system, diphenyliodonium triflate serves as a phenyl radical precursor, which reacts with 1-methyl-1*H* -pyrrole to afford the C–H phenylated product (Figure 4f). Under 850 nm irradiation for 40 h, complete conversion was achieved using 5 mol % **Ru-bpyap-OMe**. The formation of the phenylated product under strictly anaerobic conditions confirms that the TPA-accessed excited state of **Ru-bpyap-OMe** is also capable of engaging in an oxidative quenching pathway to generate aryl radicals.

The oxidative capacity of the NIR light-generated excited state of **Ru-bpyap-OMe** was further demonstrated under aerobic conditions. Cross-dehydrogenative coupling between 2-(4-methoxyphenyl)-1,2,3,4-tetrahydroisoquinoline and nitromethane proceeded efficiently using only 1 mol % **Ru-bpyap-OMe** under 850 nm irradiation, reaching complete conversion after 36 h (Figure 4g). In this transformation, O_2_ functions as the terminal oxidant. Similarly, cyclocondensation between *o*-phenylenediamine and 5-thiazolecarboxaldehyde to form 2-(5-thiazolyl)-1*H* -benzimidazole was achieved under identical irradiation conditions with 1 mol % photocatalyst, reaching full conversion after 15 h (Figure 4h).

For all the above reactions, control experiments confirmed that both light irradiation and the presence of **Ru-bpyap-OMe** are essential; no desired products were observed in the absence of light or photocatalyst. In short, the hydrodehalogenation study establishes a clear excitation mode transition from spin-allowed (525 nm) and spin-forbidden one-photon excitation (660 nm) to two-photon excitation (740 nm) as the irradiation wavelength is tuned from the visible to the NIR region. The successful execution of the four reactions upon 850 nm irradiation further validates **Ru-bpyap-OMe** as a competent TPA photocatalyst capable of harvesting low-energy NIR photons to drive synthetically meaningful chemical reactions.

### Panchromatic Photopolymerization

After establishing the wavelength-dependence of the photocatalytic performance of **Ru-bpyap-OMe**, we next evaluated its application in NIR light-triggered photoredox-catalyzed free-radical polymerization, a transformation that directly probes light penetration and practical scalability. Pentaerythritol triacrylate (PETA) was selected as a multifunctional acrylate monomer, and diphenyliodonium triflate (DPI) was employed as a radical initiator (Figure 5a). Upon irradiation, the excited **Ru-bpyap-OMe** is expected to reduce DPI to generate phenyl radicals, which initiate acrylate polymerization. To quantitatively evaluate NIR light penetration through light-absorbing media, a series of optical filter solutions were prepared to selectively attenuate specific spectral regions (Figure 5b). Selected inorganic salts, CoCl_2_, K_2_Cr_2_O_7_, NiSO_4_, and Cr­(NO_3_)_3_, as well as organic dyes Rhodamine 6G, Rhodamine B, methylene blue, and indocyanine green were dissolved at concentrations affording an absorbance of approximately 1.0 at their respective absorption maxima. These filter solutions span the UV–visible range and effectively block corresponding wavelengths while transmitting NIR photons (e.g., 850 nm). The polymerization mixture, containing [PETA]/[DPI]/[Ru-bpyap-OMe] = 200/1/0.01, was prepared in DMSO and loaded into a 150 mm-long NMR tube, which was then immersed in a 20 mL vial (22 mm diameter) containing the filter solution (Figure 5c). Each filter condition was tested in an independent experiment to avoid cross-contamination of optical effects. TPA absorption by **Ru-bpyap-OMe** upon irradiation with 850 nm through the filter medium resulted in the reduction of DPI and initiation of the polymerization reaction. Despite significant attenuation of UV and visible light by the filter solutions, self-supporting polymer gels were obtained within 40 min to 8 h depending on the filter composition (Figure 5d and Supporting Information). The successful formation of gels under all filter conditions confirms that polymerization can be driven by NIR excitation rather than residual shorter-wavelength transmission. These results directly demonstrate the penetration advantage of NIR light and validate **Ru-bpyap-OMe** as an effective TPA photocatalyst for bulk free-radical polymerization.

**Figure 5:**
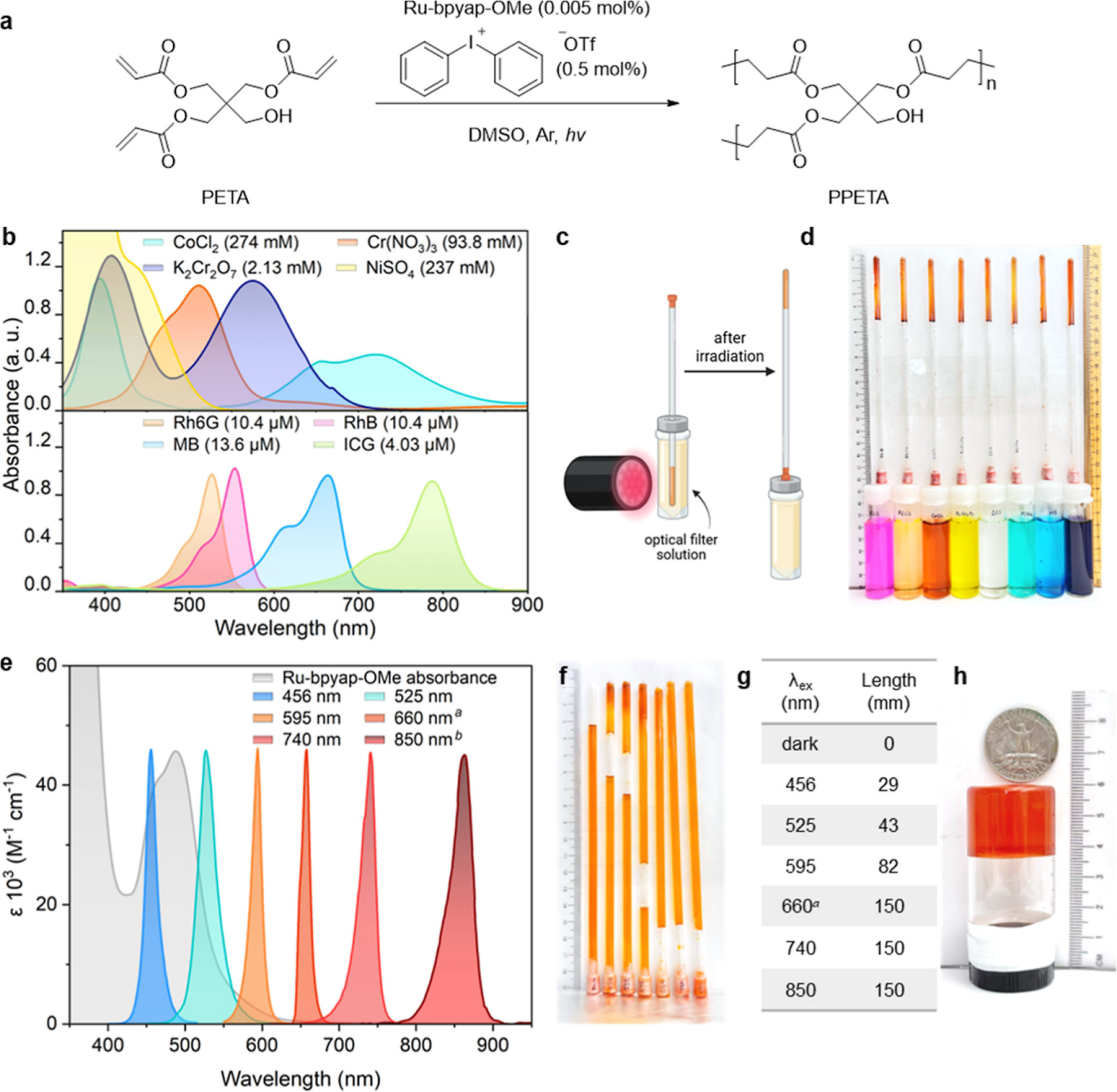
(a) Photopolymerization of pentaerythritol triacrylate (PETA) catalyzed by **Ru-bpyap-OMe**. (b) Absorption spectra of the optical filter solutions at the indicated concentrations. (c) Schematic illustration of the experimental setup employing optical filter solutions. (d) Photograph of the polymer gels formed upon irradiation at 850 nm through different optical filter solutions. From left to right, the optical filter solutions are Rhodamine B, Rhodamine 6G, CoCl_2_, K_2_Cr_2_O_7_, indocyanine green, NiSO_4_, methylene blue, and Cr­(NO_3_)_3_. (e) Absorption spectrum of Ru-bpyap-OMe (gray) and the normalized emission spectra of 456, 525, 595, 660, 740, and 850 nm LEDs. ^*a*^The 660 nm LED was equipped with a 645 nm long-pass optical filter. ^*b*^The 850 nm LED emission spectrum was obtained from Thorlabs. (f) Photograph of the polymer gels formed after 2 h irradiation at different wavelengths. From left to right: dark control and excitation at 456, 525, 595, 660, 740, and 850 nm. (g) Polymer gel lengths obtained after 2 h irradiation at different wavelengths. (h) PPETA gel prepared on a 10 g scale upon irradiation at 850 nm.

To further assess its panchromatic photocatalytic behavior, the same polymerization formulation was used to compare penetration depth under different excitation wavelengths. The emission spectra of the available LEDs (Figure 5e and see Supporting Information for specifications) reveal broad emission profiles centered at 456, 525, 595, 660, 740, and 850 nm. An NMR tube containing the polymerization mixture (150 mm length) was wrapped with aluminum foil to eliminate side excitation, ensuring that light entered exclusively from the bottom. After 2 h of irradiation, the extent of gel formation was measured as a function of wavelength (Figure 5f,g). No gelation was observed in the dark control, nor in parallel experiments performed without **Ru-bpyap-OMe** under irradiation at 525–850 nm, confirming that gel formation at these wavelengths is strictly photoinduced and dependent on the photocatalyst. In the presence of **Ru-bpyap-OMe**, under 456 nm irradiation, polymerization occurred only near the illuminated base, producing a gel length of 29 mm, consistent with strong absorption and rapid attenuation of blue light within the reaction medium. In contrast, the control experiment without photocatalyst gave only a 9 mm residual gel at this wavelength, indicating that gelation remains predominantly photocatalyst-driven even in the blue region (Figure S22). As the excitation wavelength increased, the gel length increased markedly. Irradiation at 525 nm produced deeper curing relative to 456 nm, while deep-red and NIR excitation (660, 740, and 850 nm) generated full-length (150 mm) self-supporting gels within 2 h (Figure 5g). The dramatic increase in effective penetration depth at longer wavelengths reflects reduced scattering and absorption losses, combined with the ability of **Ru-bpyap-OMe** to access the same reactive excited state via spin-forbidden one-photon absorption or direct two-photon absorption pathways. Finally, the practical scalability of this NIR light-driven polymerization was demonstrated in a 10 g-scale experiment conducted in a 20 mL vial under 850 nm irradiation for 2 h (Figure 5h). The formation of a uniform, self-supporting polymer mass confirms that TPA-driven photoredox initiation can be translated from small-scale NMR tubes to preparative-scale polymer synthesis. Collectively, these results highlight not only the excitation versatility of **Ru-bpyap-OMe** across UV–visible–NIR regimes but also the tangible advantages of panchromatic photocatalysis for applications requiring deep light penetration and scalable material fabrication.

Compared with other strategies developed for long-wavelength photocatalysis, **Ru-bpyap-OMe** offers a distinct combination of properties. Osmium-based complexes extend absorption into the red but generally access lower excited-state energies and weaker reducing power, while TTA-UC systems require carefully matched sensitizer–annihilator pairs and are operationally more demanding. Heterogeneous and nanoparticle-based photocatalysts, in turn, often suffer from limited substrate scope in homogeneous solutions. In contrast, within a single homogeneous molecular catalyst, **Ru-bpyap-OMe** combines photocatalytic activity at irradiation wavelengths as long as 850 nm with a long-lived, redox-competent excited state. This combination is particularly relevant to biological and optically dense media, where the long excitation wavelength provides deep penetration while the retention of high excited-state energy and competent redox power supports demanding transformations. Such a profile is difficult to achieve with one-photon long-wavelength systems, in which direct absorption of low-energy photons generally yields short-lived or weakly reactive excited states.

## Conclusion

This work reports a practical design route toward homogeneous ruthenium photocatalysts in which distinct, wavelength-dependent excitation mechanisms converge on a common reactive state to enable panchromatic catalysis across the visible-to-NIR window, with a two-photon absorption cross-section large enough to drive organic reactions using inexpensive NIR LEDs. Through extended π-conjugation and electronic modulation in **Ru-bpyap-OMe**, we demonstrate its broadband activation spanning the visible to NIR region while converging on the same long-lived (∼1.5 μs) and redox-active MLCT state. Spectroscopic, computational, and wavelength-dependent kinetic analyses collectively reveal how distinct excitation pathways operate across different spectral regimes. In particular, deep-red and NIR excitation enables reactivity in optically dense environments and bulk media where conventional visible-light photocatalysis is limited. The successful implementation of photopolymerization under 850 nm irradiation highlights the practical advantages of long-wavelength activation, including enhanced penetration depth and scalability. In short, our results offer a practical design route toward homogeneous ruthenium photocatalysts that remain effective across the visible-to-NIR window.

## Supplementary Material



## Notes

Methods for the synthesis, characterization, and photophysical studies of photocatalysts; additional photocatalytic experimental details, results, and methods, including photographs of experimental setup; ESI–MS, NMR spectra; DFT, TD-DFT, and EDDM calculation results (PDF)
