# Supplementary material for: Panchromatic Photocatalysis using a Ruthenium Polypyridyl Complex
Source: J Am Chem Soc. 2026 Jul 27;148(30):32440–50. doi: 10.1021/jacs.6c09162 (PMC13450383; doi:10.1021/jacs.6c09162)
Supplement: Supplementary file 1 [file ja6c09162_si_001.pdf]

Supporting Information

Panchromatic Photocatalysis Using a Ruthenium Polypyridyl Complex

Minling Zhong[1], Joohyun Lee[2], Jie Huang[3], Jeanette A. Krause[1], Kaiqi Long[4], Claudia Turro[3,*] Christopher G. Elles[2,*] & Yujie Sun[1,*]

[1]Department of Chemistry, University of Cincinnati, Cincinnati, OH 45221, United States.

[2]Department of Chemistry, University of Kansas, Lawrence, Kansas 66045, United States.

[3]Department of Chemistry and Biochemistry, The Ohio State University, Columbus, OH 43210, United States.

[4]Brigham and Women's Hospital, Harvard Medical School, Boston, MA 02115, United States.

[*]E-mail: turro.1@ ohio.edu; elles@ku.edu; yujie.sun@uc.edu

Contents

1. Experimental section 2
2. Synthesis and characterization 4
3. Crystallization method and crystal data 6
4. Photophysical and electrochemical data 9
5. Power-dependent emission spectra 13
6. Photocatalytic Experiments 15
7. Computational Details 19
8. Photocatalysis using Ru-bpyap-OMe 27
9. NMR Spectra and mass spectra 35
10. Cartesian coordinates at the optimized geometry 52
11. Reference 56

1. Experimental section

Materials and Instrumentation. Commercial reagents were purchased from Sigma Aldrich, Alfa Aesar, Ambeed and TCI and used as received without any further purification. ^{1}H, ^{13}C and ^{19}F NMR spectra were recorded with Bruker Advanced III or I (500 or 400 MHz) spectrometers. All chemical shifts (δ) were reported in ppm relative to d-solvent peaks. Multiplicity was indicated as follows: s (singlet), d (doublet), t (triplet), q (quartet), m (multiplet), dd (doublet of doublet), br (broad). Coupling constants were reported in hertz (Hz). UV-vis spectra measurements were recorded on a Cary 8454 UV-vis spectrophotometer (Agilent Technologies) using a quartz cuvette (10 × 10 mm, 3.5 mL). Emission spectra were recorded using a HORIBA Fluorolog-QM-75-21-C spectrophotometer. UPLC-MS analyses for Ru complexes were conducted with an Agilent 6530 QTOF Mass Spectrometer with 1290 Infinity Binary UPLC. LC-MS analysis for 5-trifluoromethylation-2-methylfuran was conducted with a Bruker timsTOF mass spectrometer.

Transient Absorption Spectroscopy. Femtosecond transient absorption spectra were performed on a home-built system. The 800 nm (8 mJ 1 kHz rep. rate, ~35 fs pulse width) laser beam is generated from an Astrella laser system then split by the Ti:Saph crystal to form the pump beam (3 mJ) and probe beam. The pump beam goes through an optical parametric amplifier (Coherent OPerA Solo) to generate the desire wavelengths (3-4 μJ). Samples are loaded into a flow cell opened to air. All samples were at a concentration that afforded an absorption of ~0.6 − 0.8 at the sample excitation wavelength. Fluorescence emission decay was measured on a LP980 spectrometer system (Edinburgh Instruments). The 355 nm laser beam is generated by a frequency-tripled Quanta-Ray INDI Nd:YAG laser (Spectra-Physics, ~6 ns pulses at 10 Hz) then goes through a tunable optical parametric oscillator (Spectra-Physics) to output the desired wavelengths, and the white light probe beam is generated from a 150 W Xe arc lamp. Single wavelength emission decay traces were collected using a PMT and digital oscilloscope (Tektronix MDO3022, 200 MHz, 2.5 GS/s). Spectral measurements were collected using an iCCD camera (iStar, Andor Technology). All the air-tight solutions were purged with N_2 for 15 min before experiments. The absorbance of the solutions was kept 0.6 − 0.8 at the excited wavelength. The absorption spectra were taken before and after the experiments to confirm no degradation over the measurements.

Two-Photon Absorption Spectroscopy. We measured broadband two-photon absorption (TPA) spectra using a pump-probe method with variable-wavelength excitation and broadband probe pulses.[1] The ~220 fs pump pulses for this measurement come from the output of an amplified Yb:KGW laser with an optical parametric amplifier (Light Conversion, Carbide + i-OPA) operating at 1 kHz. We used <1.5 μJ pump pulses at wavelengths ranging from 700 to 950 nm in 50 nm increments, or at the 1030 nm laser fundamental, and we obtained broadband probe pulses via white-light continuum

generation in a YAG substrate. The pump and probe pulses overlap in a 1 mm path length cuvette containing a 5-10 mM solution of the target compound in acetonitrile. The broadband TPA spectrum is obtained from the simultaneous absorption of the pump and probe light in the sample, where the excitation energy is equal to the sum of the individual pump and probe photon energies. We used only the portion of the probe light that is individually non-resonant with the linear (one-photon) absorption spectrum of the sample, and we used the overlapping stimulated Raman band of the solvent C-H stretching vibrations as an internal standard for calibrating the TPA cross section measurement.[2] This approach provides TPA cross sections that are accurate within <10% uncertainty. We combined the spectra at all pump wavelengths into a single broadband 2PA spectrum covering transition wavelengths over the range from 300 to 470 nm, where the transition wavelength is equivalent to the wavelength for one-photon absorption at the same energy as the sum of the pump and probe photons.

Power-dependent emission spectra. We used two different laser systems operating at 1 kHz repetition rate for the power-dependence emission measurements. A Ti:Sapphire laser (Coherent, Legend) produced <80 fs pump pulses at 650, 740, 850, and 990 nm via a tunable optical parametric amplifier (Light Conversion, TOPAS), whereas the Yb:KGW laser (Light Conversion, Carbide) produced ~220 fs pump pulses at 343, 515, 650, 850, and 1000 nm. For the Yb:KGW laser, we used an optical parametric amplifier (Light conversion, i-OPA) to generate the output from 650 to 1000 nm, and we used second harmonic generation (SHG) and third harmonic generation (THG) of the laser fundamental to generate 515 and 343 nm, respectively. We focused the pump beams into the sample using a 75-mm focal length lens and recorded the resulting emission spectrum at 90° using a pair of lenses to collect and focus the light into a fiber optic for delivery to a spectrometer (StellarNet, BLACK-Comet). The TPA measurements used samples with concentrations up to 10 mM in a square fluorescence cell with 3 mm path length (Starna), while the OPA measurements used lower-concentration samples in a square fluorescence cell with 1 cm path length (Starna). To adjust pump power, we used two continuously variable circular neutral density filters. The pump power was measured using a semiconductor energy sensor for the Ti:Sapphire laser (Coherent, LabMax J-10MT) and a thermal power sensor for the Yb:KGW laser (Coherent, PowerMax-USB PS10). The error in measuring the average pump power was less than 5% in each case. Emission measurements with the two lasers gave consistent results at 650, 850, and 990/1000 nm, with no noticeable changes due to the different pulse duration. An inner-filter effect was responsible for reabsorption of some emission in the higher-concentration samples, but otherwise the emission spectra were independent of excitation wavelength.

2. Synthesis and characterization

Synthesis of bpyap ligands

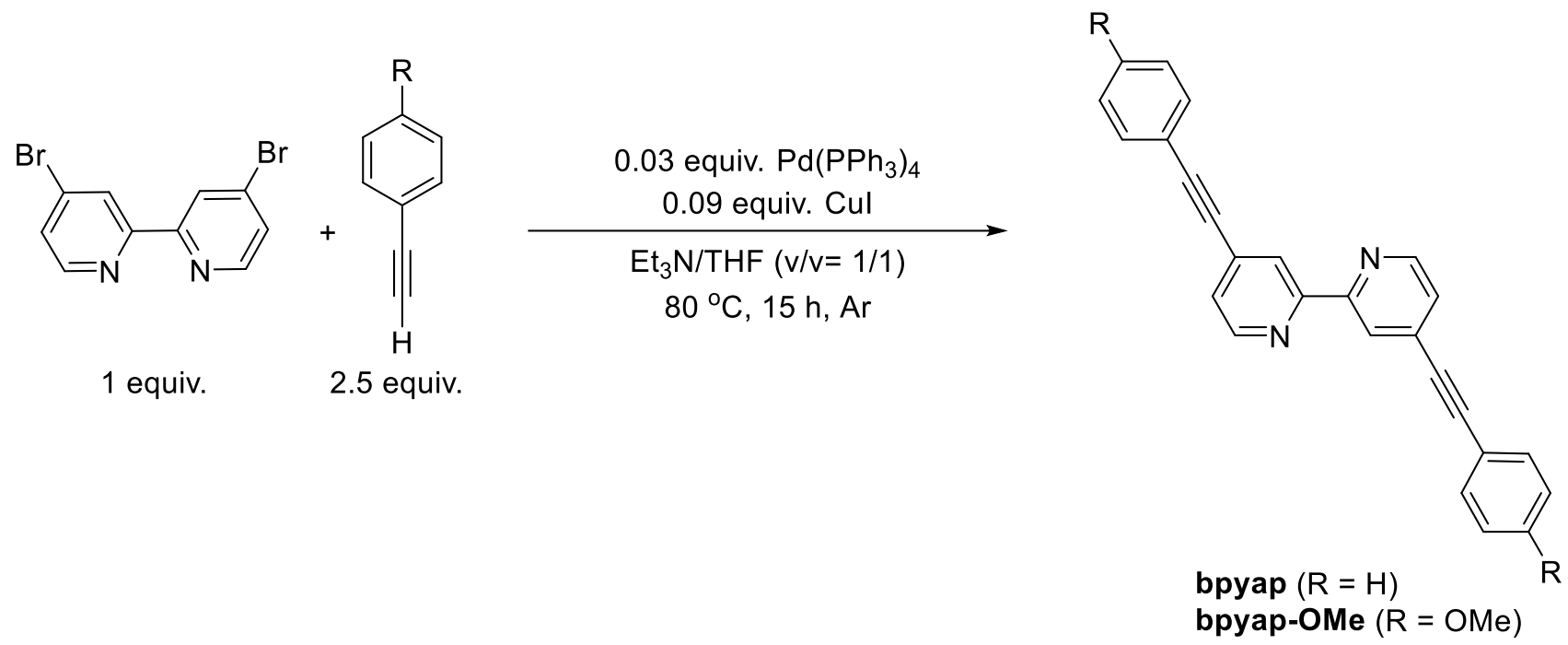

4,4'-Dibromo-2,2'-bipyridine (942 mg, 3 mmol, 1.0 equiv.), $Pd(PPh_3)_4$ (104 mg, 0.09 mmol, 3 mol%), and CuI (51 mg, 0.27 mmol, 9 mol%) were dissolved in THF/ Et_3N (20 mL,1:1 v/v) and loaded into a 100-mL round-bottom flask. After being purged with argon for 15 min, the reaction mixture was heated to 80 °C, and a solution of phenylacetylene (766 mg, 7.5 mmol, 2.5 equiv.) in THF/ Et_3N (20 mL,1:1 v/v) was then added dropwise. The resulting reaction mixture was stirred and refluxed at 80 ℃ for 15 h. After cooling to room temperature, the solvent was removed under reduced pressure. The crude product was purified on silica gel using hexane/dichloromethane as the eluent (gradient from 0 to 5:1 to 1:1, v/v). The desired product **bpyap** was obtained as a white solid (806 mg, 2.26 mmol, 75%).

^{1}H NMR (500 MHz, $CDCl_3$) δ 8.68 (d, J = 4.9 Hz, 2H), 8.54 (s, 2H), 7.58 (dd, J = 6.9, 3.0 Hz, 4H), 7.44 – 7.41 (m, 2H), 7.41 – 7.37 (m, 6H) ppm.

^{13}C{^{1}H} NMR (126 MHz, $CDCl_3$) δ 155.82, 149.38, 132.72, 132.09, 129.33, 128.65, 125.67, 123.42, 122.36, 94.24, 87.12 ppm.

Using a similar procedure, **bpyap-OMe** was synthesized from *p*-methoxyphenylacetylene and isolated as a pale-yellow solid (961 mg, 2.31 mmol, 77%).

^{1}H NMR (500 MHz, $CDCl_3$) δ 8.66 (d, J = 4.9 Hz, 2H), 8.51 (s, 2H), 7.54 – 7.48 (m, 4H), 7.39 (d, J = 4.8 Hz, 2H), 6.94 – 6.88 (m, 4H), 3.85 (s, 6H) ppm.

^{13}C{^{1}H} NMR (126 MHz, $CDCl_3$) δ 160.47, 155.81, 149.32, 133.68, 133.07, 125.45, 123.25, 114.43, 114.32, 94.56, 86.15, 55.51 ppm.

Synthesis of Ru-bpyap complexes

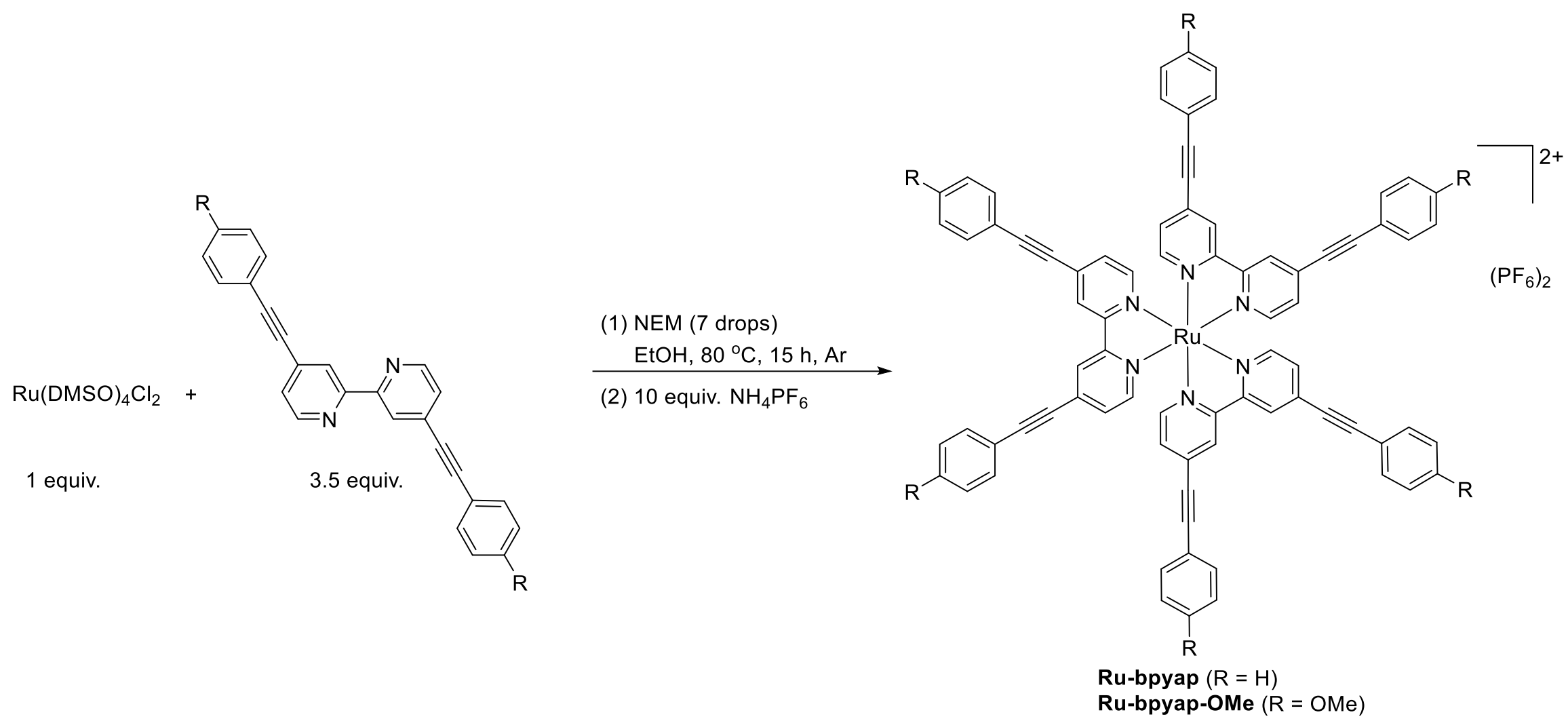

The $Ru(DMSO)_4Cl_2$ (145 mg, 0.3 mmol) and **bpyap** (374 mg, 1.05 mmol, 3.5 equiv.) were suspended in ethanol (40 mL). *N*-Ethylmorpholine (7 drops) was added, and the resulting mixture was purged with argon for 15 min and refluxed at 80 °C for 15 h. After cooling to room temperature, the resulting dark solution was filtered to remove any insoluble material, and an aqueous solution of $[NH_4][PF_6]$ (3 mmol, 10 equiv.) was added dropwise. The dark precipitate that formed was collected via centrifugation and recrystallized from acetonitrile/diethyl ether. The desired product **Ru-bpyap** was obtained as a dark red solid (330 mg, 0.23 mmol, 76%).

^{1}H NMR (500 MHz, CD_3CN) δ 8.66 (s, 6H), 7.77 (d, *J* = 5.9 Hz, 6H), 7.64 (d, *J* = 7.1 Hz, 12H), 7.48 (dd, *J* = 9.8, 7.0 Hz, 24H) ppm.

^{13}C{^{1}H} NMR (126 MHz, CD_3CN) δ 157.62, 152.85, 133.87, 133.07, 131.37, 129.98, 129.95, 127.25, 122.02, 99.00, 86.34 ppm.

^{19}F NMR (376 MHz, CD_3CN) δ -72.85 (d, *J* = 706.6 Hz) ppm.

UPLC-MS (ESI-positive mode) calc. for $[C_{78}H_{48}N_6Ru]^{2+}$ m/z: 585.1487. Found: 585.1557.

Using a similar procedure, **Ru-bpyap-OMe** was synthesized from **bpyap-OMe** and isolated as a dark red solid (349 mg, 0.21 mmol, 71%).

^{1}H NMR (400 MHz, CD_3CN) δ 8.64 (s, 6H), 7.77 (d, *J* = 5.9 Hz, 6H), 7.61 (s, 12H), 7.48 (dd, *J* = 5.9, 1.7 Hz, 6H), 7.05 (d, *J* = 8.8 Hz, 12H), 3.88 (s, 18H) ppm.

^{13}C{^{1}H} NMR (101 MHz, CD_3CN) δ 162.38, 157.60, 152.65, 134.92, 134.23, 129.57, 126.89, 115.64, 113.83, 99.80, 85.64, 56.30 ppm.

^{19}F NMR (376 MHz, CD_3CN) δ -72.87 (d, *J* = 706.3 Hz) ppm.

UPLC-MS (ESI-positive mode) calc. for $[C_{84}H_{60}N_6O_6Ru]^{2+}$ m/z: 675.1809. Found: 675.1826.

3. Crystallization method and crystal data

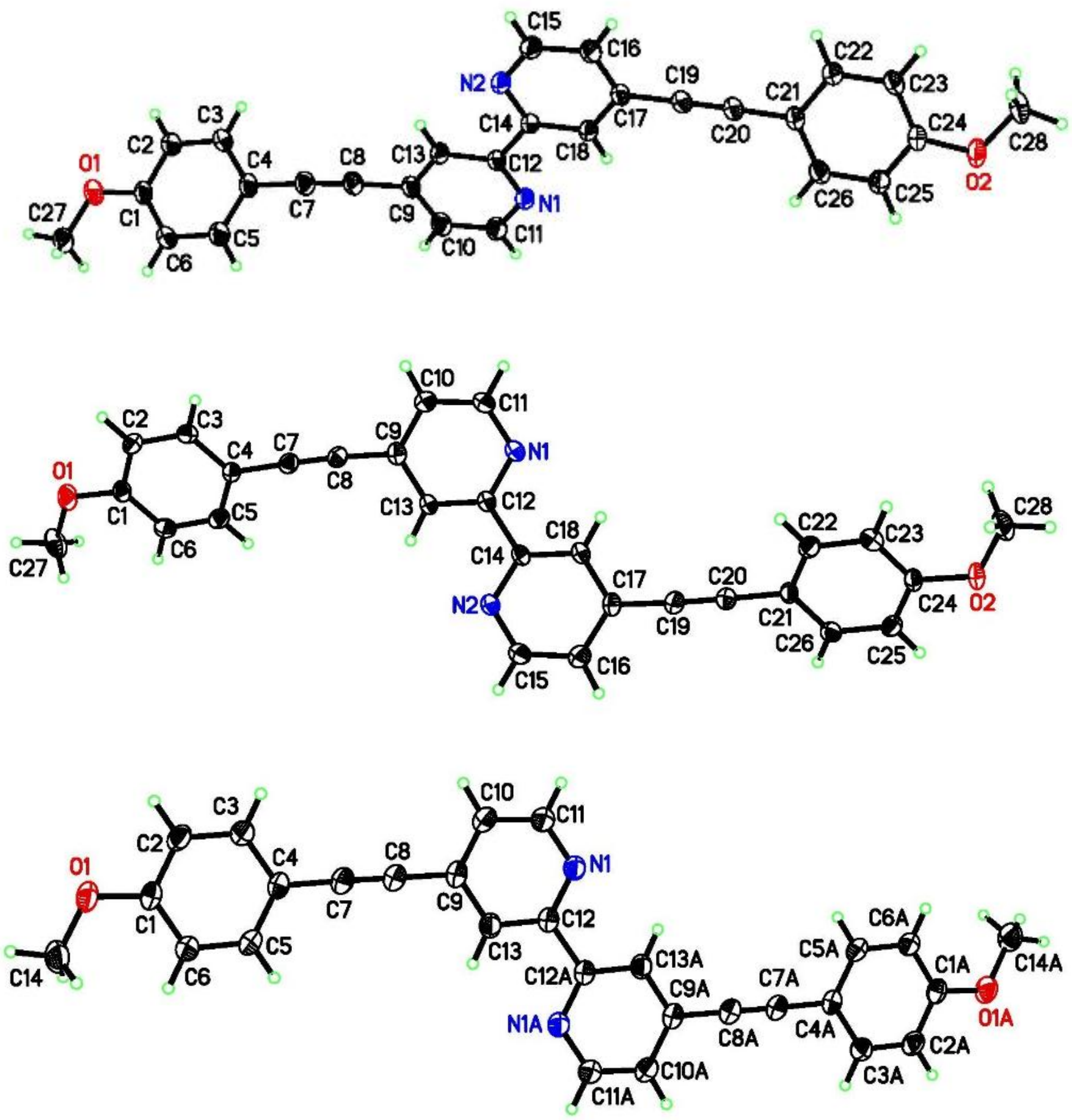

Figure S1. Molecular structure of **bpyap-OMe** (top: Polymorph I, middle: Polymorph II, bottom: Polymorph III) (anisotropic displacement ellipsoids are set at the 50% probability level).

Selected bond distances (Å) and angles (°):

	Polymorph I	Polymorph II		Polymorph III
C(4)-C(7)	1.4365(16)	1.434(3)	C(4)-C(7)	1.4318(13)
C(7)-C(8)	1.1970(16)	1.197(3)	C(7)-C(8)	1.1959(15)
C(8)-C(9)	1.4363(16)	1.434(3)	C(8)-C(9)	1.4351(14)
C(12)-C(14)	1.4855(15)	1.487(3)	C(12)-C(12A)	1.4904(18)
C(17)-C(19)	1.4320(15)	1.437(3)	C(8A)-C(9A) [a]	1.4351(14)
C(19)-C(20)	1.2004(16)	1.194(3)	C(7A)-C(8A) [a]	1.1959(15)
C(20)-C(21)	1.4316(15)	1.442(3)	C(4A)-C(7A) [a]	1.4318(13)
N(1)-C(12)-C(14)	116.47(9)	116.54(16)	N(1)-C(12)-C(12A)	116.50(10)
N(2)-C(14)-C(12)	116.22(9)	115.95(16)	N1A)-C(12A)-C(12) [a]	116.50(10)
C(8)-C(7)-C(4)	175.76(14)	178.2(2)	C(8)-C(7)-C(4)	178.99(11)
C(7)-C(8)-C(9)	176.33(14)	176.1(2)	C(7)-C(8)-C(9)	179.26(11)
C(20)-C(19)-C(17)	177.56(13)	175.4(2)	C(7A)-C(8A)-C(9A) [a]	179.26(11)
C(19)-C(20)-C(21)	178.35(13)	177.3(2)	C(8A)-C(7A)-C(4A) [a]	178.99(11)
N(1)-C(12)-C(14)-N(2)	179.70(10)	168.26(18)	N(1)-C(12)-C(12A)-N(1A)	180

[a]Symmetry transformation used to generate equivalent atoms for Polymorph III: -x+1, -y+1, -z.

Three polymorphs of **bpyap-OMe** were obtained from slow evaporation of different solvents at room temperature. Polymorph I (0.378 x 0.135 x 0.015 mm^3) grew from N,N-dimethylformamide, Polymorph II (0.228 x 0.048 x 0.015 mm^3) from dichloromethane, and Polymorph III (0.195 x 0.076 x 0.070 mm^3) from a mixture of tetrahydrofuran and diethyl ether.

For X-ray examination and data collection, suitable crystals were mounted in a loop with Paratone-N oil and transferred to the goniostat bathed in a cold N_2 stream. Intensity data for all three polymorphs were collected at 150K on a Bruker D8 Venture Mo-IµS Photon-II diffractometer, λ=0.71073Å. Data collection frames were measured in shutterless mode. The data frames were processed using the program SAINT. The data were corrected for decay, Lorentz and polarization effects as well as absorption and beam corrections based on the multi-scan technique.

The structure was solved by a combination of direct methods and the difference Fourier technique as implemented in the SHELX suite of programs and refined by full-matrix least squares on F^2 for reflections out to 0.75Å or 0.80Å resolution. Non-hydrogen atoms were refined with anisotropic displacement parameters. The H-atoms were calculated and treated with a riding model. The H-atom isotropic displacement parameters were defined as a*U_{eq} of the adjacent atom (a=1.5 for methyl and 1.2 for all others). The refinements converged with crystallographic agreement factors as summarized in the table below.

**CCDC No.**	**CCDC-2539228**	**CCDC-2539229**	**CCDC-2539230**
	Polymorph I	Polymorph II	Polymorph III
Formula	$C_{28}H_{20}N_2O_2$	$C_{28}H_{20}N_2O_2$	$C_{28}H_{20}N_2O_2$
Crystal system	Monoclinic	Monoclinic	Monoclinic
Space group	$P2_1/n$	$P2_1/n$	$P2_1/n$
Unit cell dimensions:			
a, Å	24.7498(15)	15.2163(5)	21.5247(9)
b, Å	12.5519(7)	7.0039(3)	5.2273(2)
c, Å	6.8116(4)	20.3720(7)	10.8904(4)
α, °	90	90	90
β, °	101.657(2)	104.2492(13)	116.7994(13)
γ, °	90	90	90
V, Å^3	2072.4(2)	2104.32(14)	1093.73(7)
Z	4	4	2
θ range data collect, °	2.998 – 28.316	2.063 – 26.379	2.915 – 28.286
Reflns collected	57616	34715	38878
Independent reflns / Rint	5130 / 0.0399	4293 / 0.0483	2694 / 0.0286
% Completeness to θ=25.242°	99.9	99.9	99.7
Data /restraints / parameters	5130 / 0 / 291	4293 / 0 / 291	2694 / 0 / 146
Final R [$I>2\sigma(I)$]	R1 = 0.0413	R1 = 0.0596	R1 = 0.0397
	wR2 = 0.1050	wR2 = 0.1563	wR2 = 0.1161
R indices (all data)	R1 = 0.0507	R1 = 0.0761	R1 = 0.0475
	wR2 = 0.1133	wR2 = 0.1691	wR2 = 0.1255
Goodness-of-Fit on F^2	1.030	1.065	1.040
Largest diff. peak / hole, eÅ^{-3}	0.258 / -0.179	0.512 / -0.306	0.248 / -0.166

Software used for the single crystal analyses: APEX3 v2019.2-37 and SAINT v8.40A, Bruker Analytical X-ray Instruments, Inc., Madison, WI; SADABS v2016/2, L. Krause, R. Herbst-Irmer, G.M. Sheldrick, D. Stalke, J. Appl. Cryst. (2015), 48, 3-10; SHELXTL v6.14, Bruker Analytical X-ray Instruments, Inc., Madison, WI; SHELX 2019/3, G.M. Sheldrick (2015) Acta Cryst., C71, 3-8.

4. Photophysical and electrochemical data

UV-vis and fluorescence spectroscopies

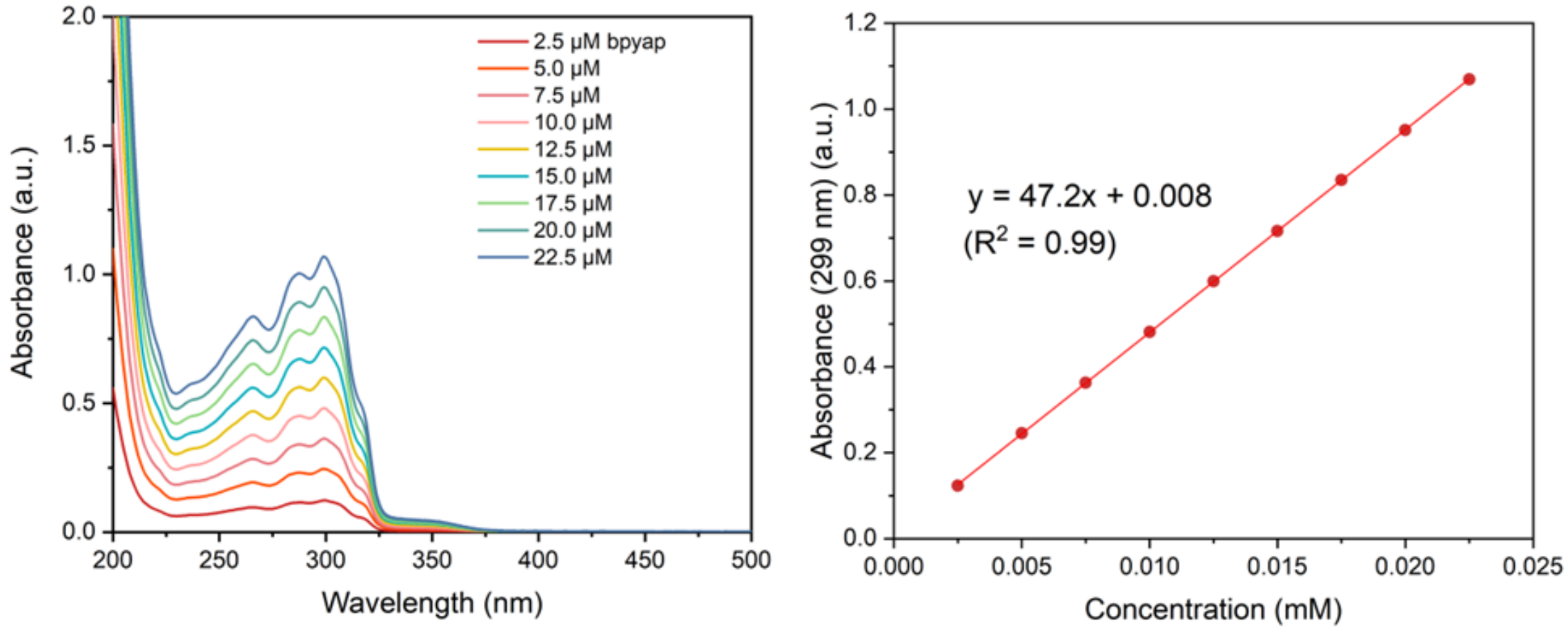

Figure S2. Absorption spectra of **bpyap** at different concentrations in CH_3CN.

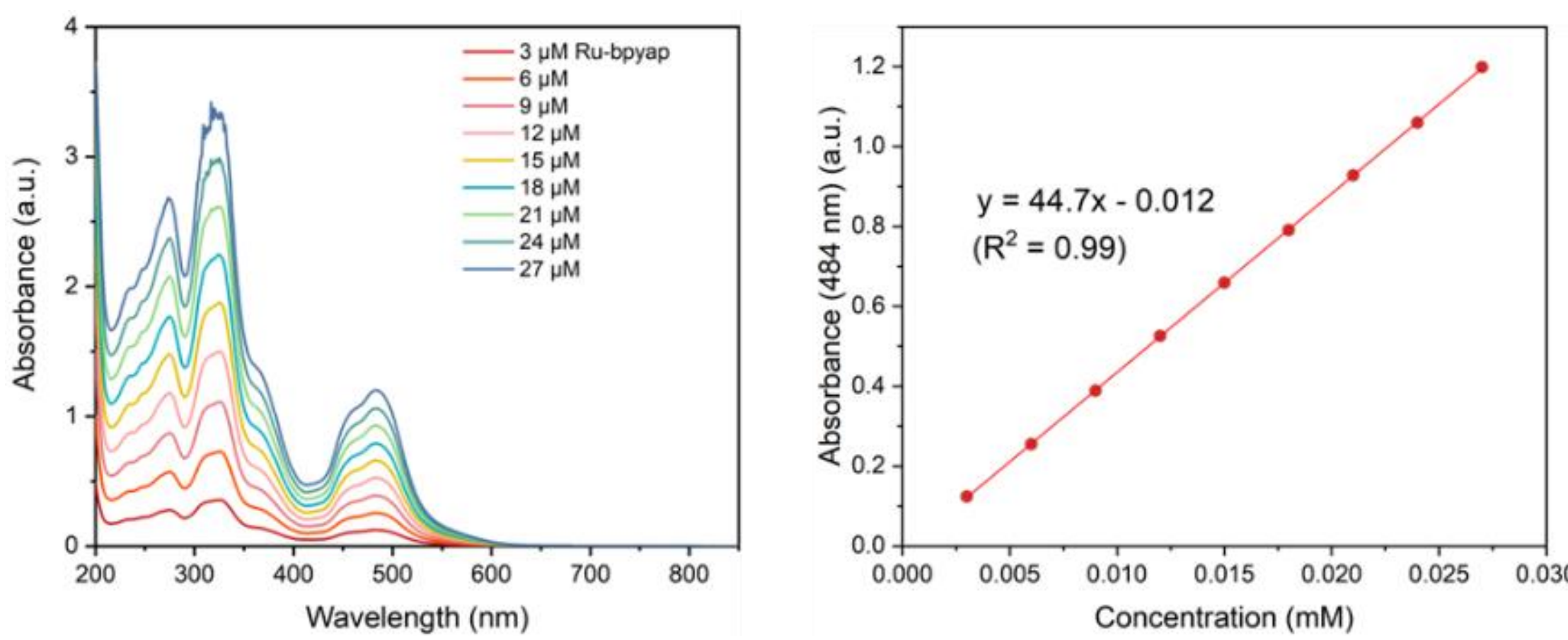

Figure S3. Absorption spectra of **Ru-bpyap** at different concentrations in CH_3CN.

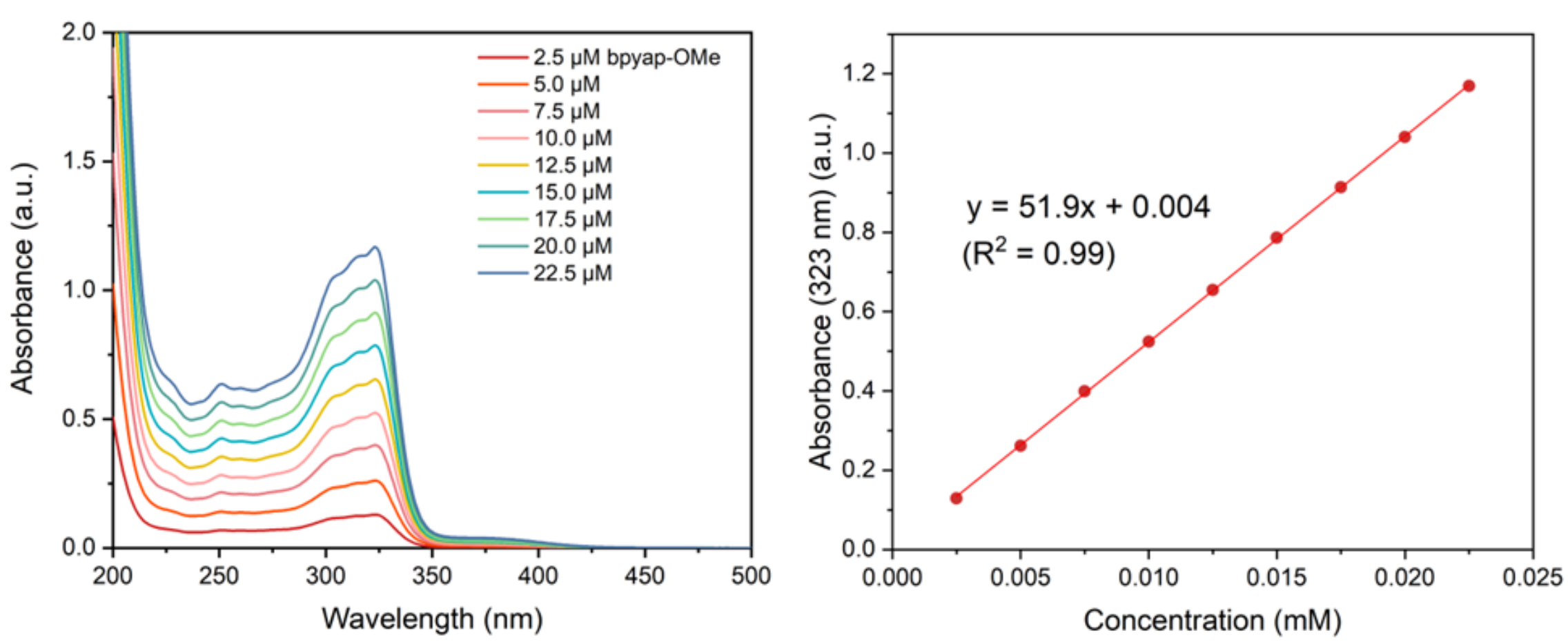

Figure S4. Absorption spectra of **bpyap-OMe** at different concentrations in CH_3CN.

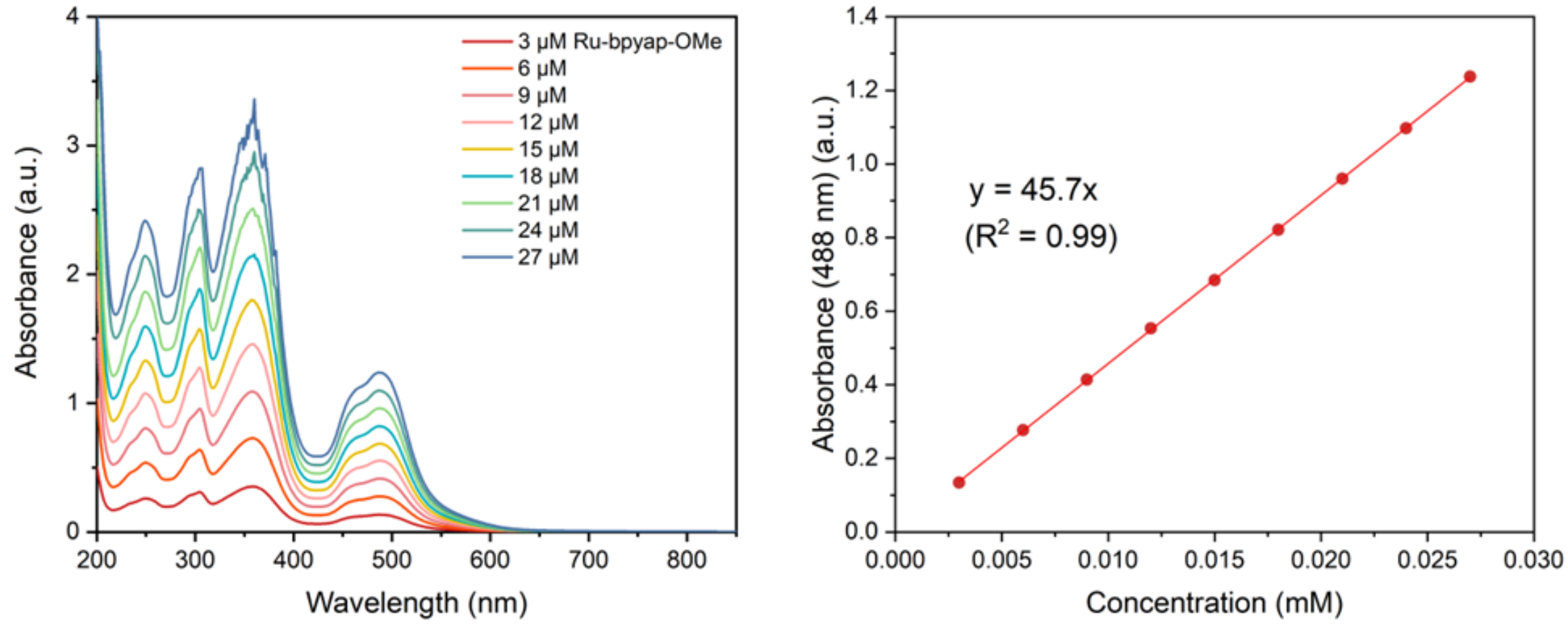

Figure S5. Absorption spectra of **Ru-bpyap-OMe** at different concentrations in CH_3CN.

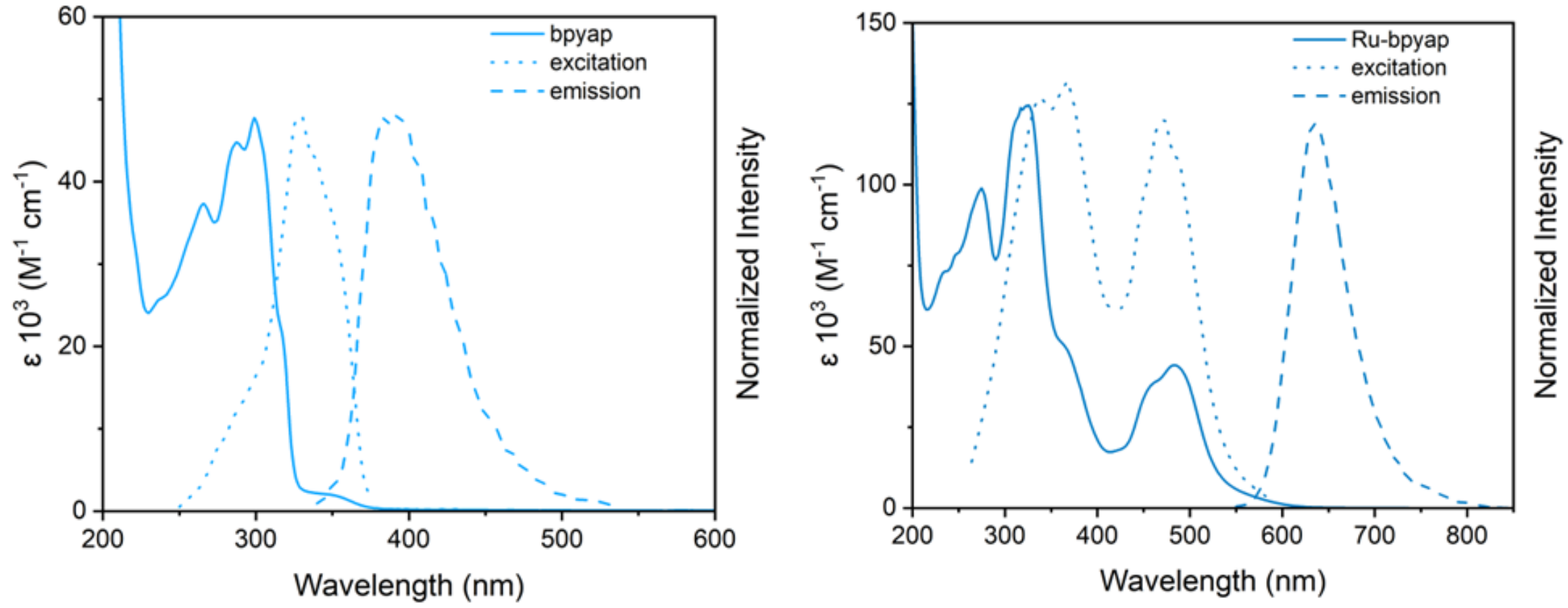

Figure S6. Absorption, excitation, and emission spectra of **bpyap** (left) and **Ru-bpyap** (right) in CH_3CN.

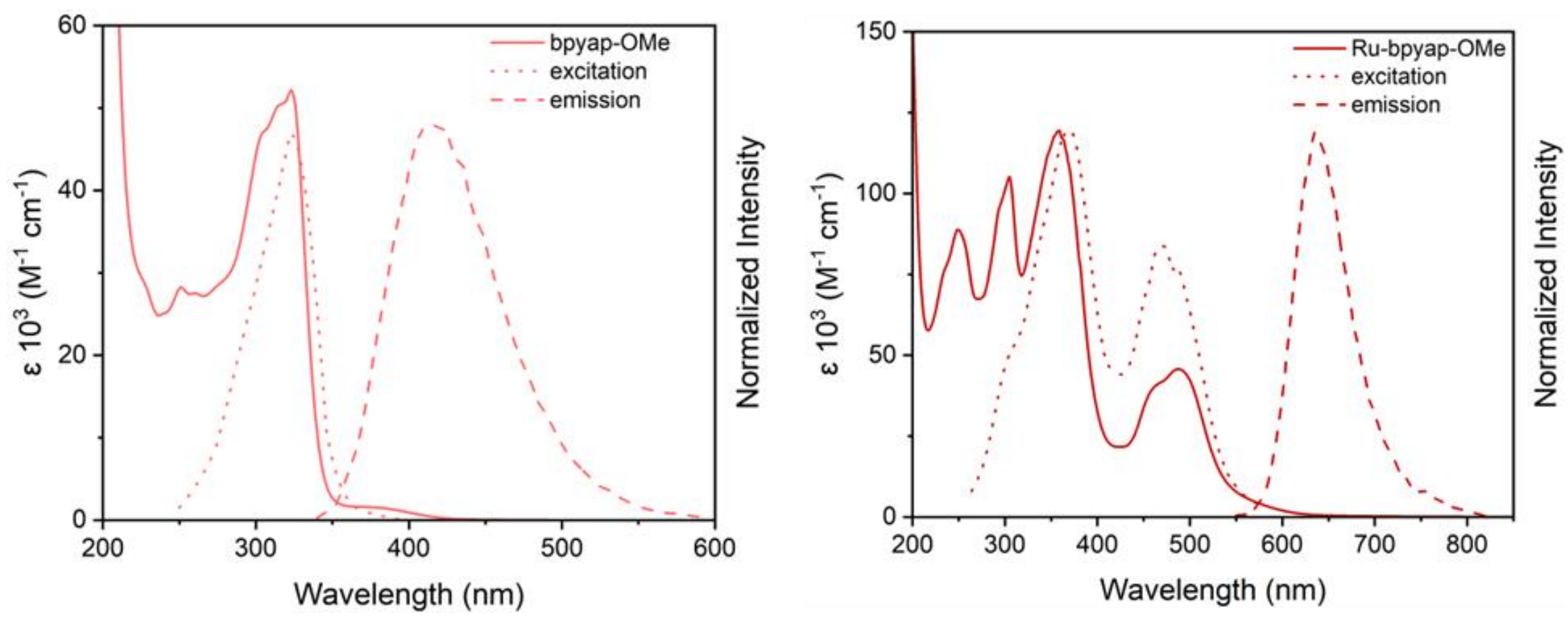

Figure S7. Absorption, excitation, and emission spectra of **bpyap-OMe** (left) and **Ru-bpyap-OMe** (right) in CH_3CN.

Electrochemistry measurements

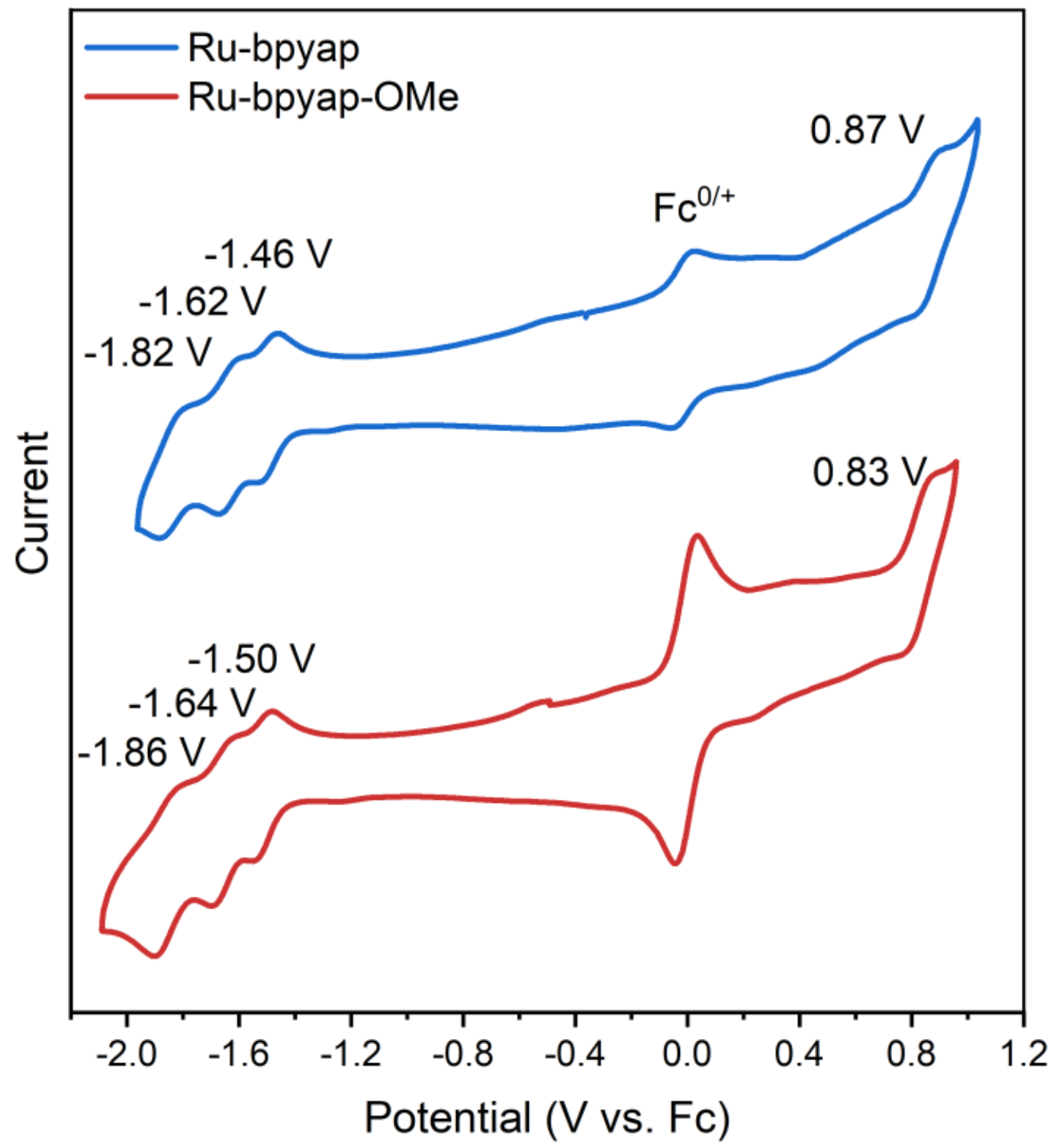

Figure S8. Cyclic voltammograms (CV) of **Ru-bpyap** and **Ru-bpyap-OMe** measured in deaerated DMF solution containing 0.1 M $LiClO_4$ as the supporting electrolyte. Three electrode system with Pt wire as the counter electrode, glassy carbon as the working electrode and Ag/AgCl as the reference electrode. Scan rate of the cyclic voltammetry is 100 mV/s.

Transient absorption spectra

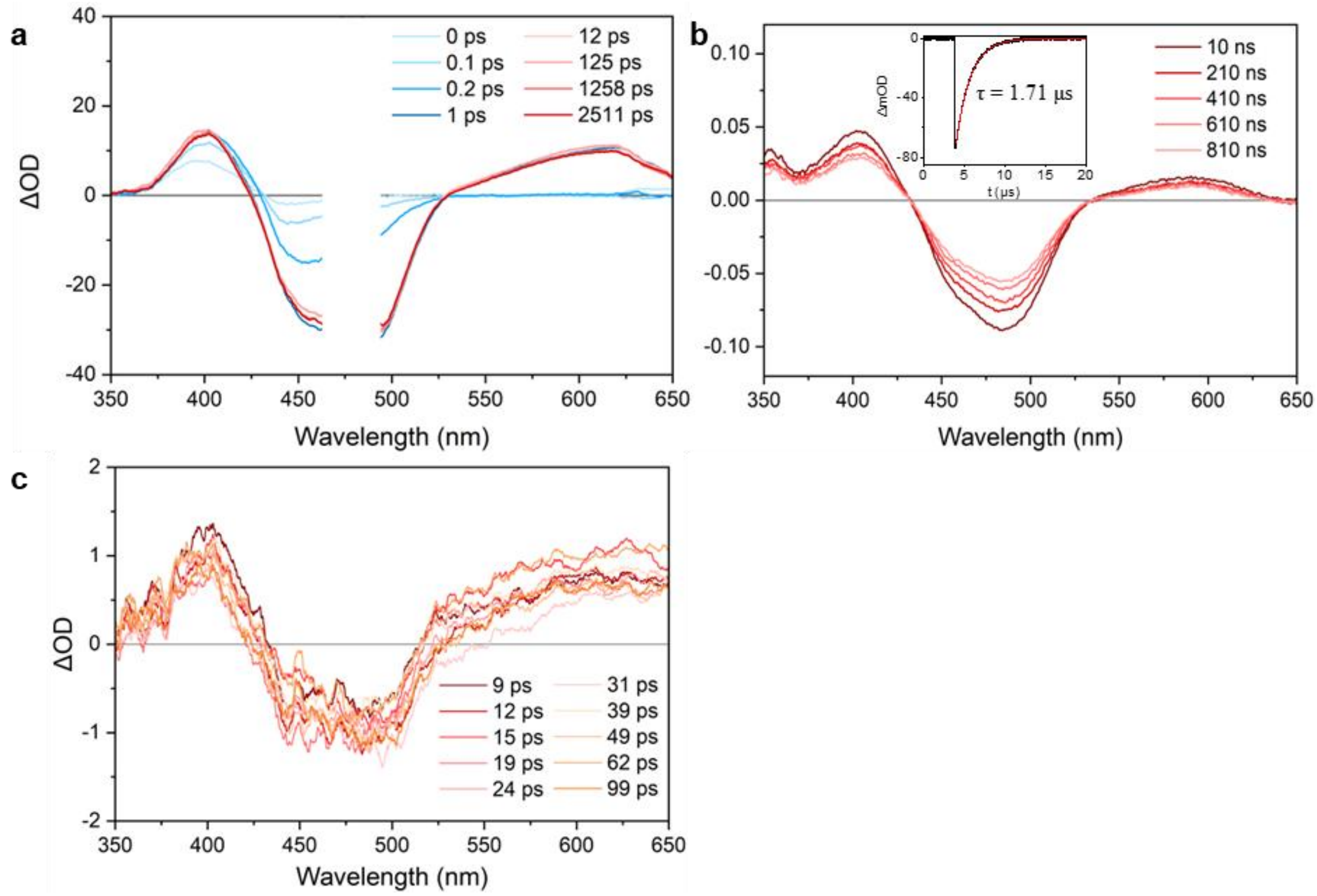

Figure S9. Transient absorption spectra of **Ru-bpyap** at different delay times: (**a**) femtosecond TA (480 nm excitation, 3 μJ), (**b**) nanosecond TA (480 nm excitation, 5 mJ), and (**c**) femtosecond TA (800 nm excitation, 60 μJ). Inset: decay trace at 485 nm with a monoexponential fit (τ = 1.71 μs).

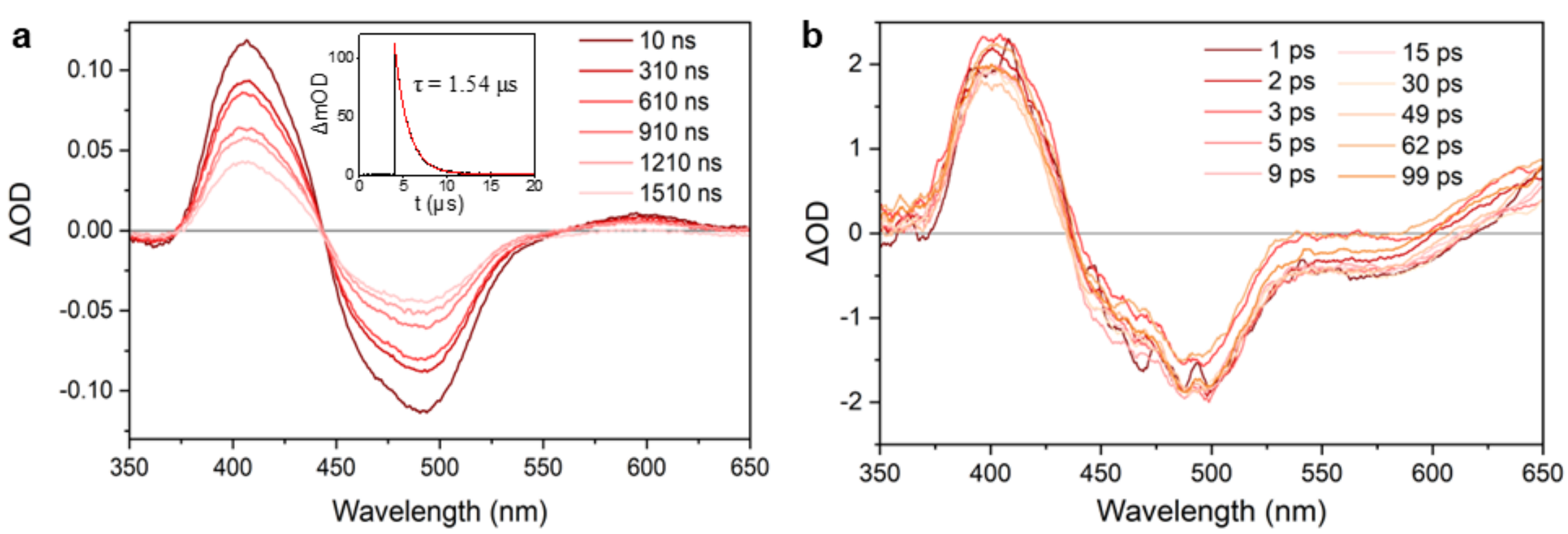

Figure S10. Transient absorption spectra of **Ru-bpyap-OMe** under irradiation of (**a**) 480 nm (3 μJ) and (**b**) 800 nm (60 μJ) at different delay times. The inset shows the kinetic decay trace monitored at 405 nm, along with a monoexponential fit yielding a lifetime of τ = 1.54 μs.

5. Power-dependent emission spectra

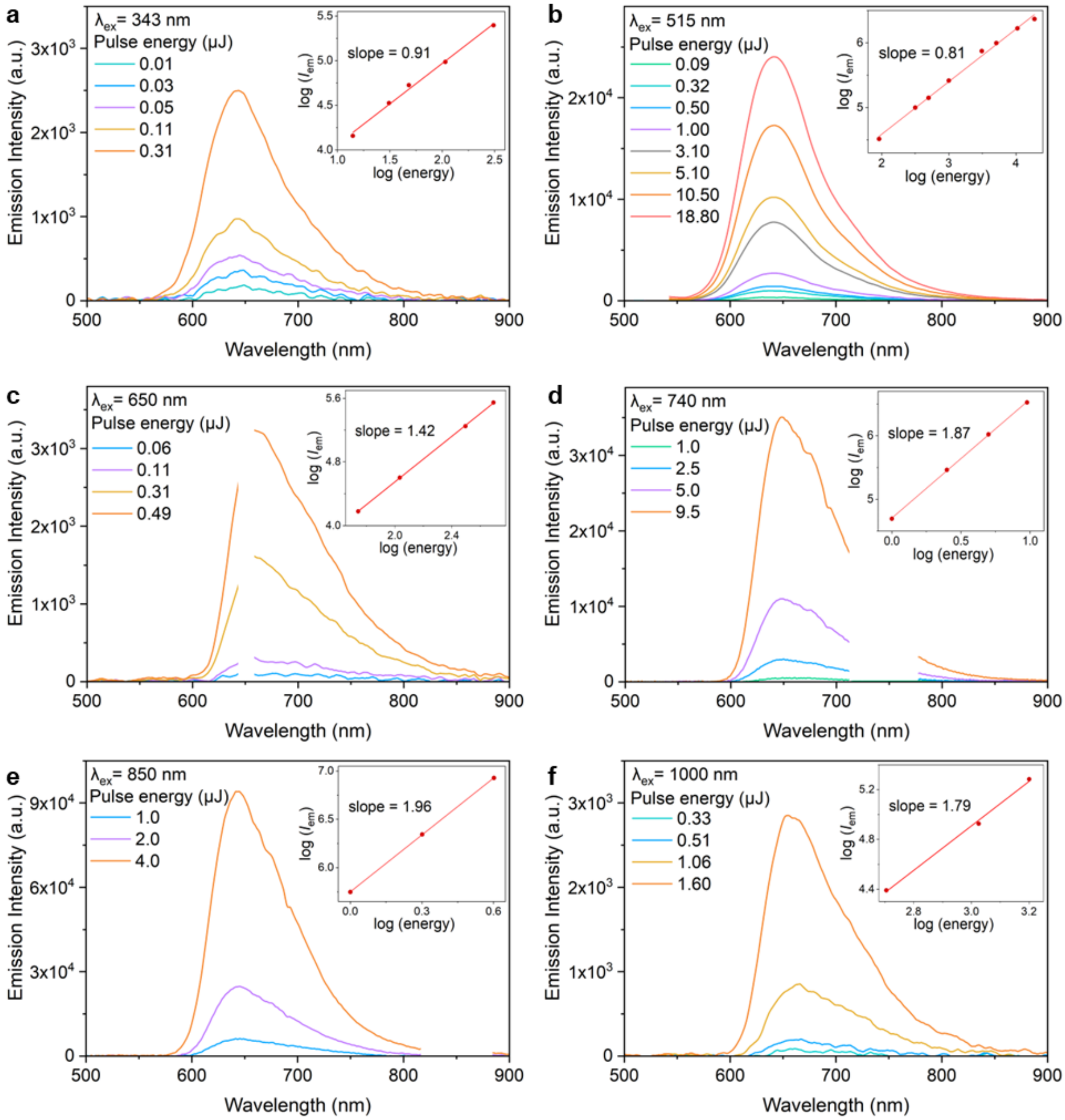

Figure S11. Power-dependent emission spectra of **Ru-bpyap** under laser excitation at (**a**) 343 nm, (**b**) 515 nm, (**c**) 650 nm, (**d**) 740 nm, (**e**) 850 nm, and (**f**) 1000 nm. Insets show linear fits of log(emission intensity) versus log(laser pulse energy). The pump-scattering regions at 515 nm, 650 nm, 740 nm and 850 nm were removed.

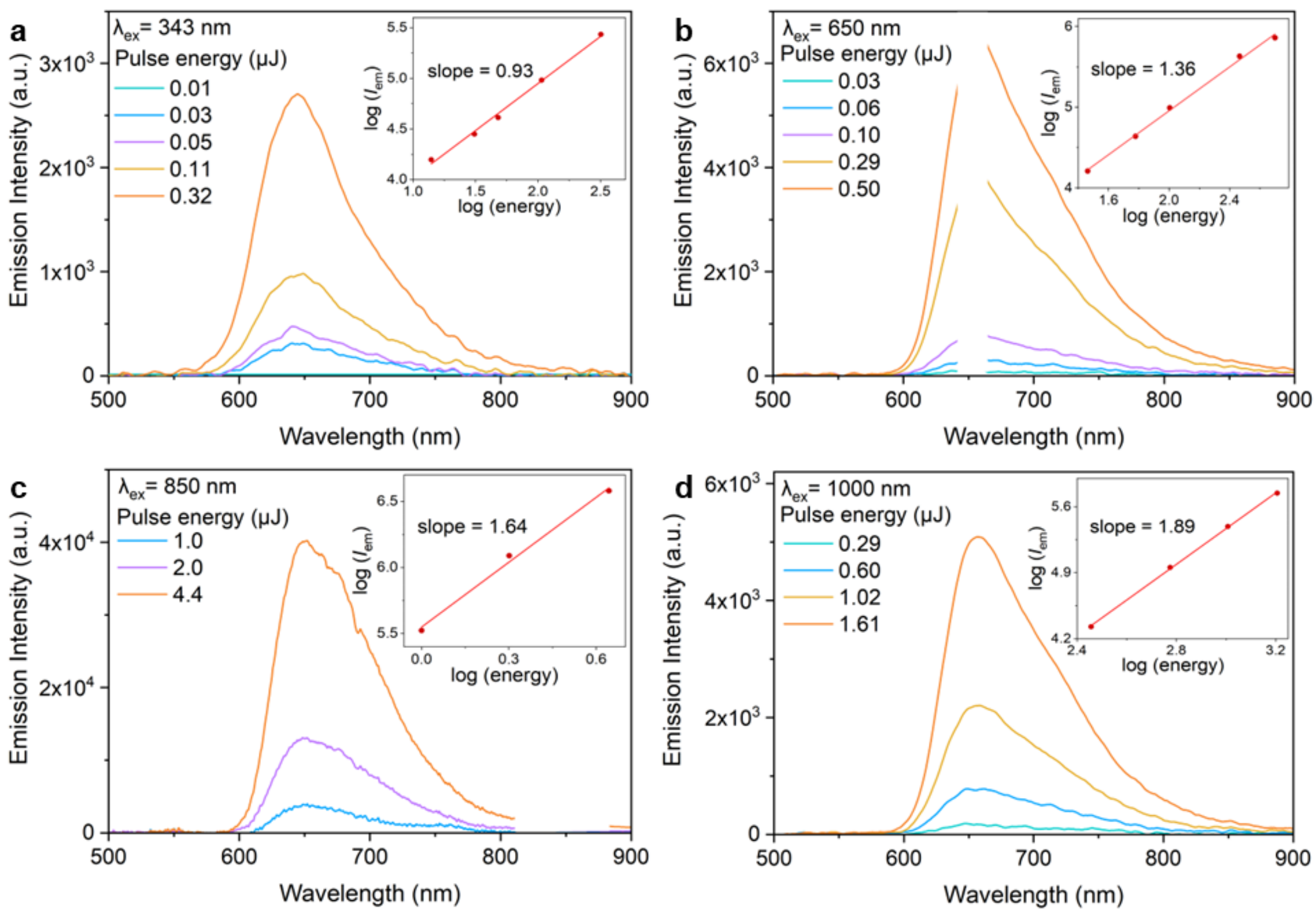

Figure S12. Power-dependent emission spectra of **Ru-bpyap-OMe** under laser excitation at (**a**) 343 nm, (**b**) 650 nm, (**c**) 850 nm, and (**d**) 1000 nm. Insets show linear fits of log(emission intensity) versus log(laser pulse energy). The pump-scattering regions at 650 nm and 850 nm were removed.

6. Photocatalytic Experiments

Light source used in this work

The 456 nm, 525 nm, 595 nm, 660 nm, and 740 nm LED lamps were purchased from Kessil. (For details, see: https://kessil.com/products/science_PR160L.php)

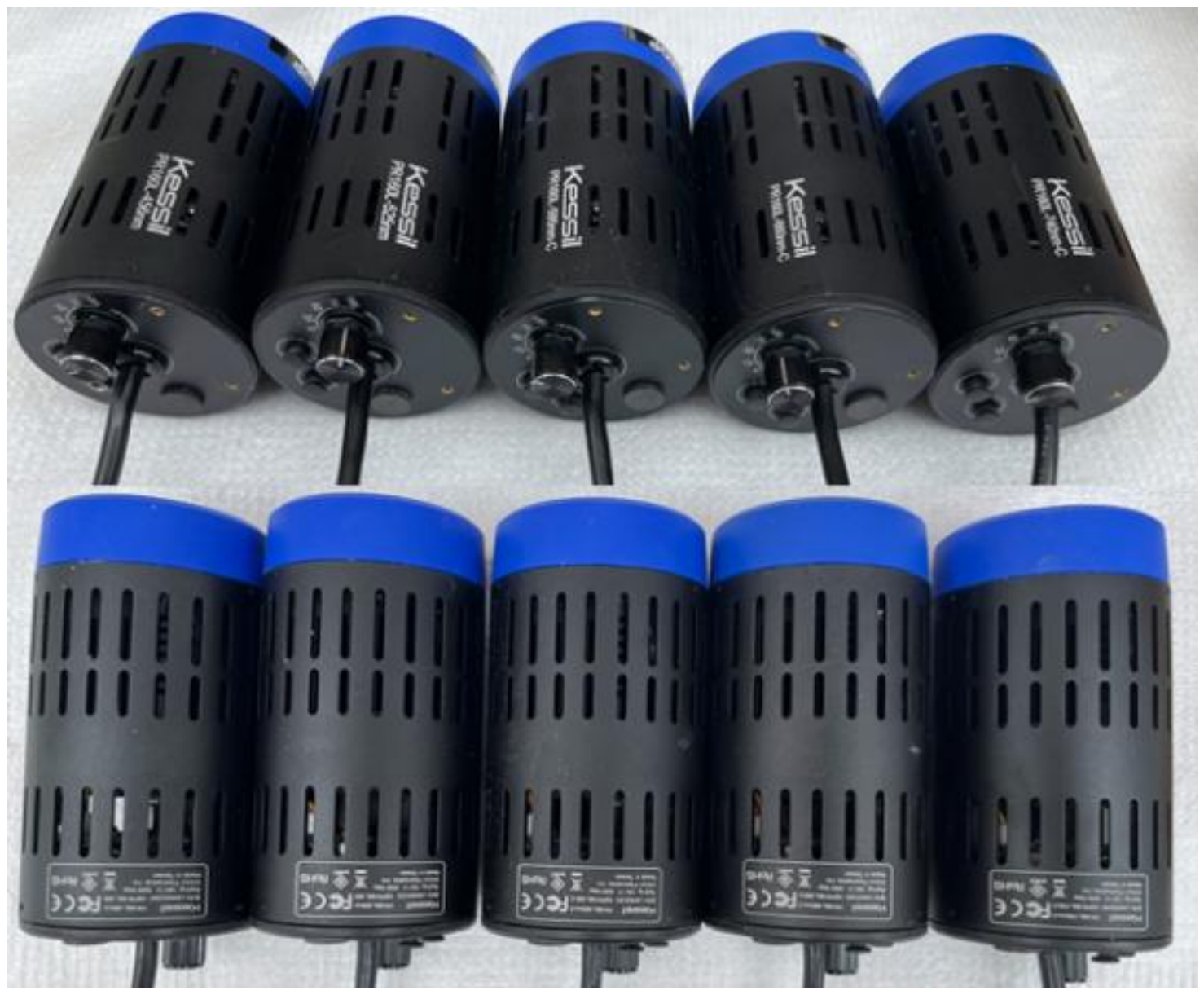

The 850 nm LED lamps used in photocatalytic reactions were purchased from Thorlabs. (For details, see: https://www.thorlabs.com/item/M850LP1)

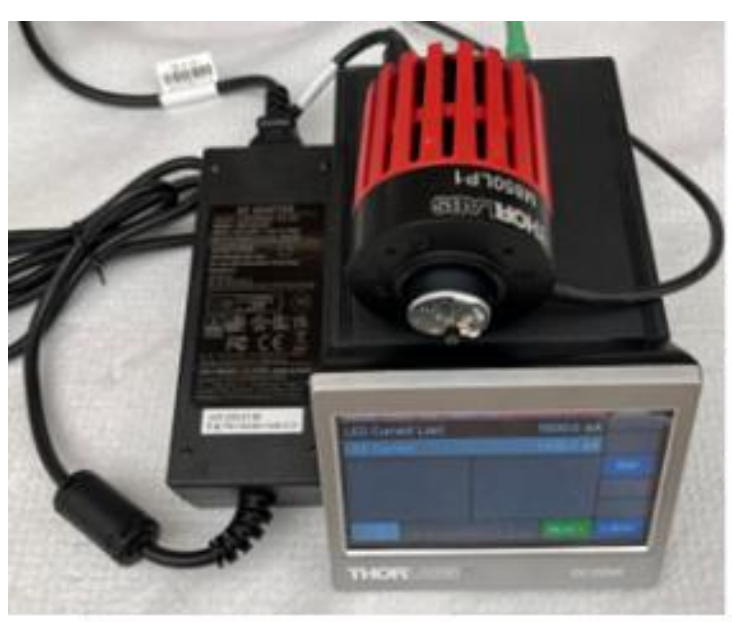

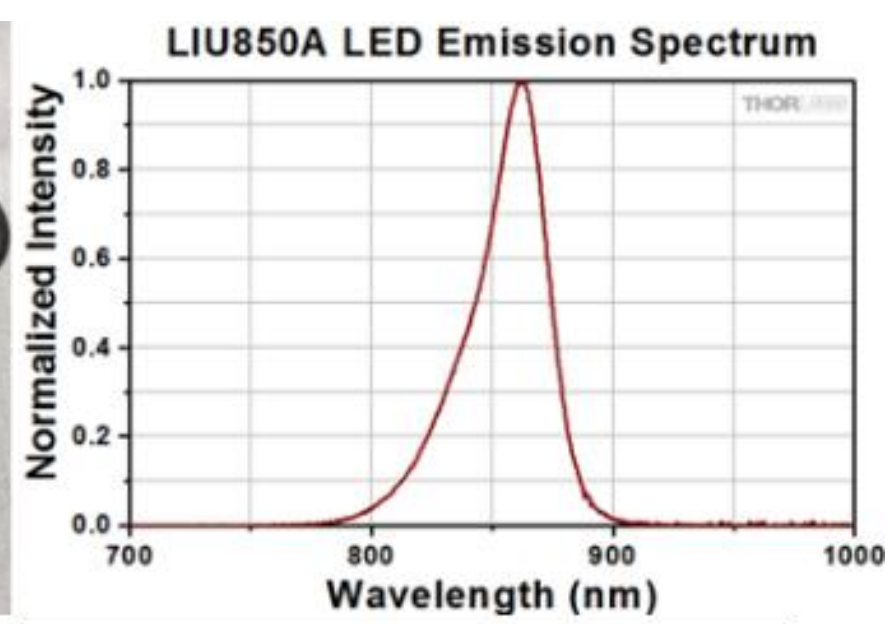

LIU850A	
Color	IR
Central Wavelength	850 nm
Intensity[a]	3.5 mW/cm^2
Total Output Power	322 mW
Supply Voltage[b]	24 V
Typical Max Current	80 mA
Operating Temperature (Non-Condensing)	0 to 40 °C
Storage Temperature	-40 to 70 °C
Weight	0.052 kg
Risk Group[c]	RG0 - Exempt Group

a. Measured at a distance of 100 mm from the LED along the emission axis.
b. The LEDs are controlled by IC drivers. The supply voltage of 24 V is required for proper operation.
c. According to the standard IEC 62471:2006, Photobiological Safety of Lamps and Lamp Systems.

The 850 nm LED lamps used in photopolymerization reactions were purchased from Shenzhen Chengzi Lighting Optoelectronics Co., Ltd. (For details, see: https://e.tb.cn/h.7wiePceq6ny1bk4?tk=XULQUOa5183)

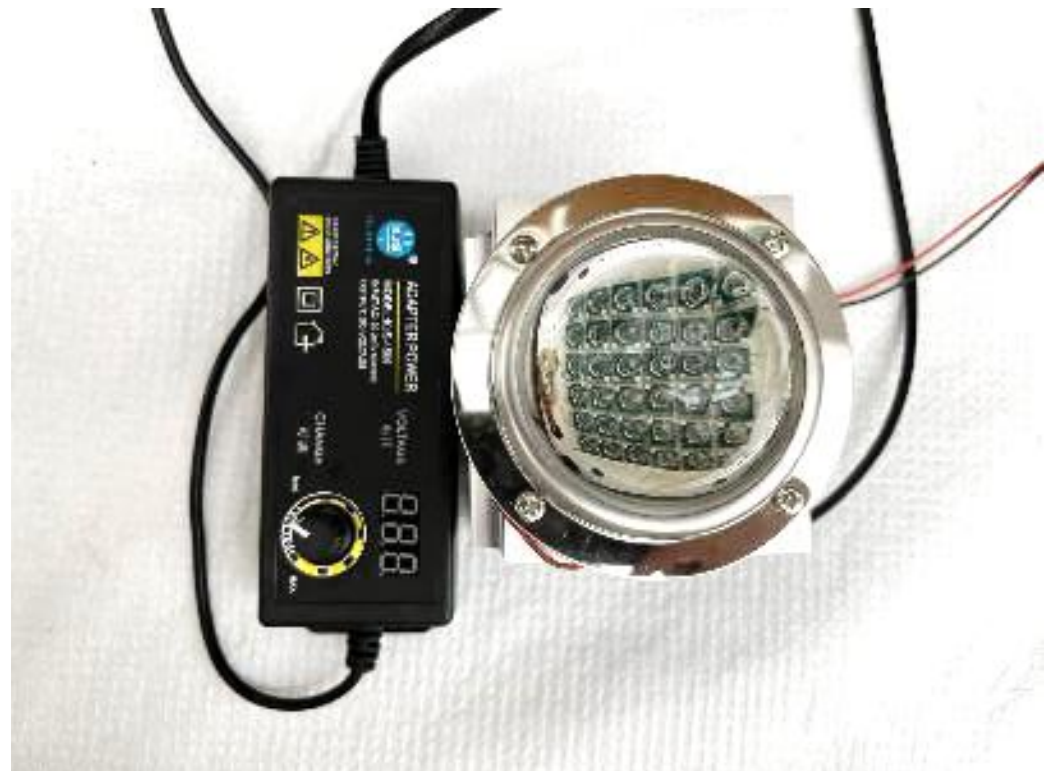

The power meters used to measure photon power density were purchased from Thorlabs. (For details, see: https://www.thorlabs.com/wireless-handheld-power-meters?tabName=Specs)

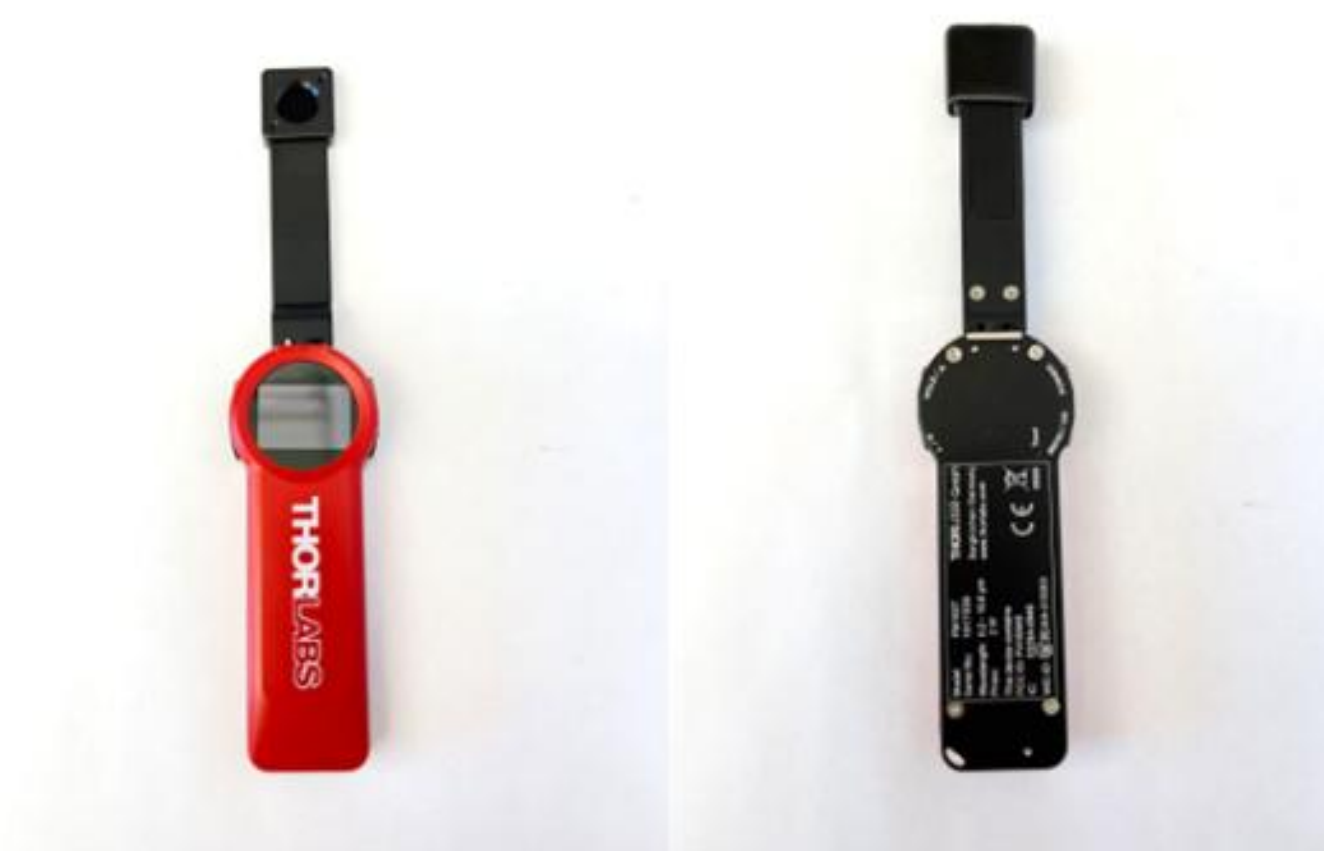

The 645 nm long-pass glass filter was purchased from Newport. (For details, see: https://www.newport.com/p/20CGA-645)

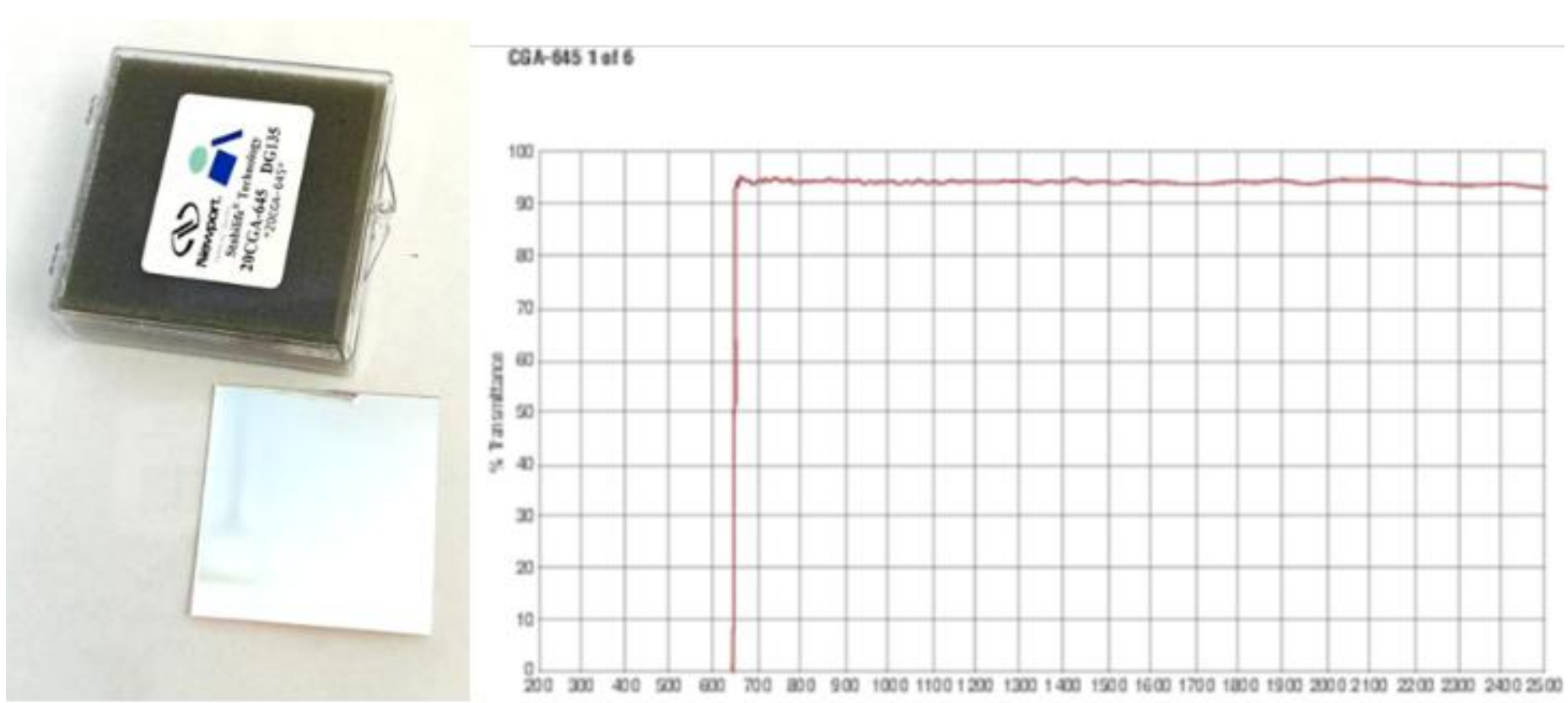

Photocatalysis set-up

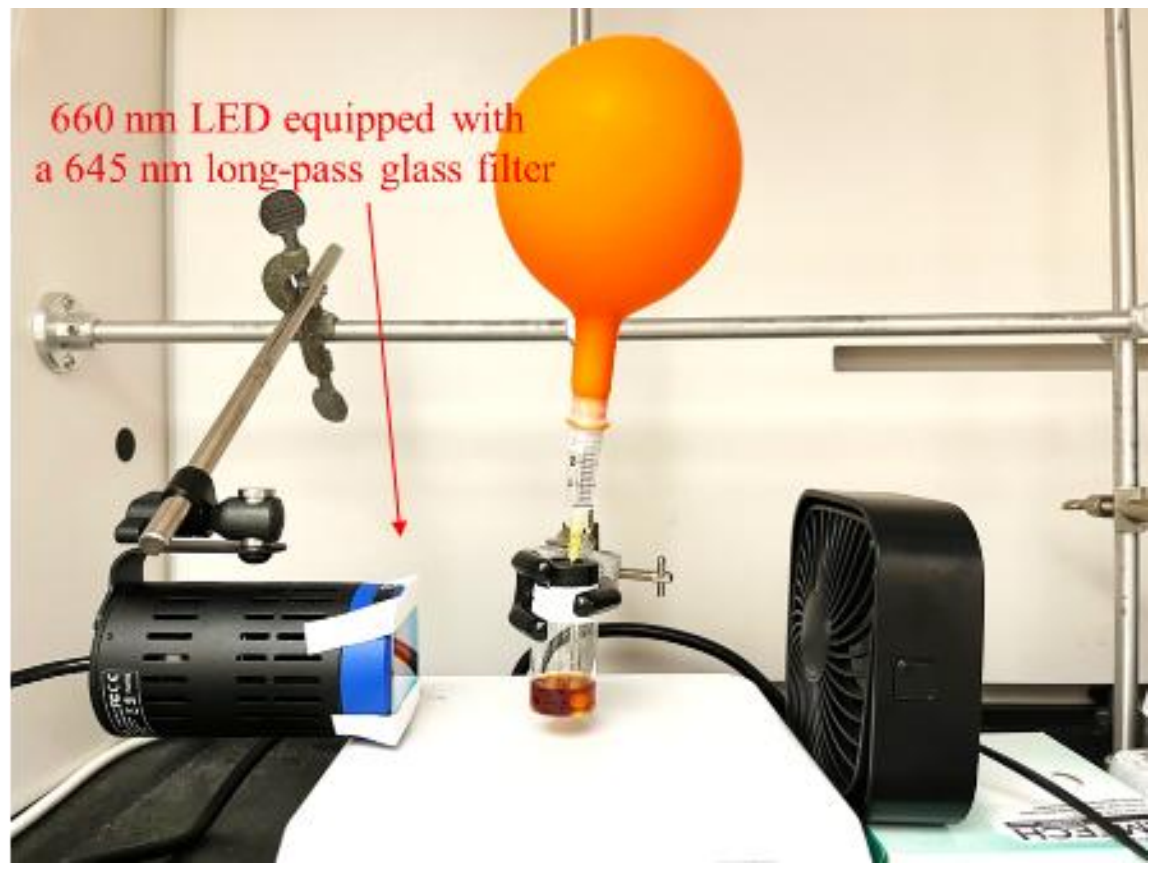

Figure S13. The experimental setup for photocatalytic hydrodehalogenation of 2-bromo-4′-fluoroacetophenone upon irradiation of 525 nm or 660 nm LED.

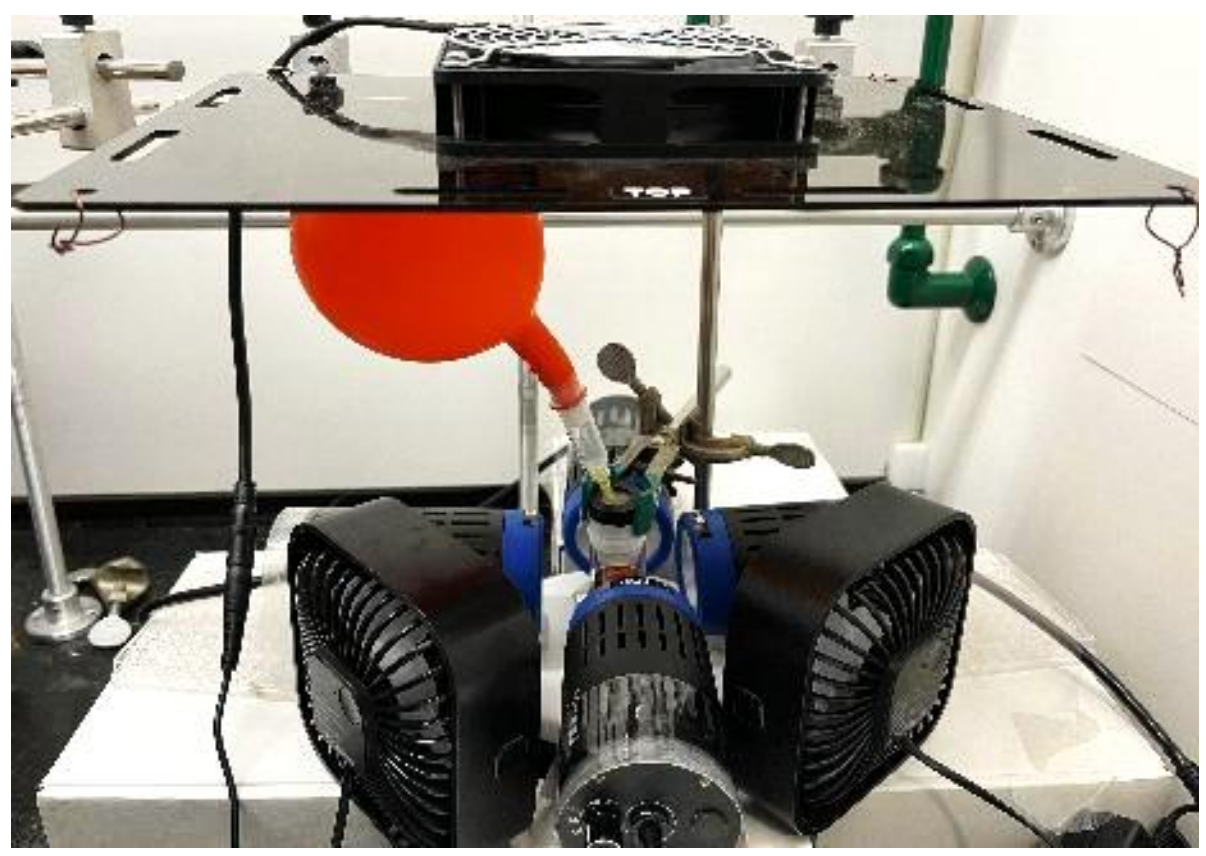

Figure S14. The experimental setup for photocatalytic hydrodehalogenation of 2-bromo-4′-fluoroacetophenone upon irradiation of 740 nm LEDs.

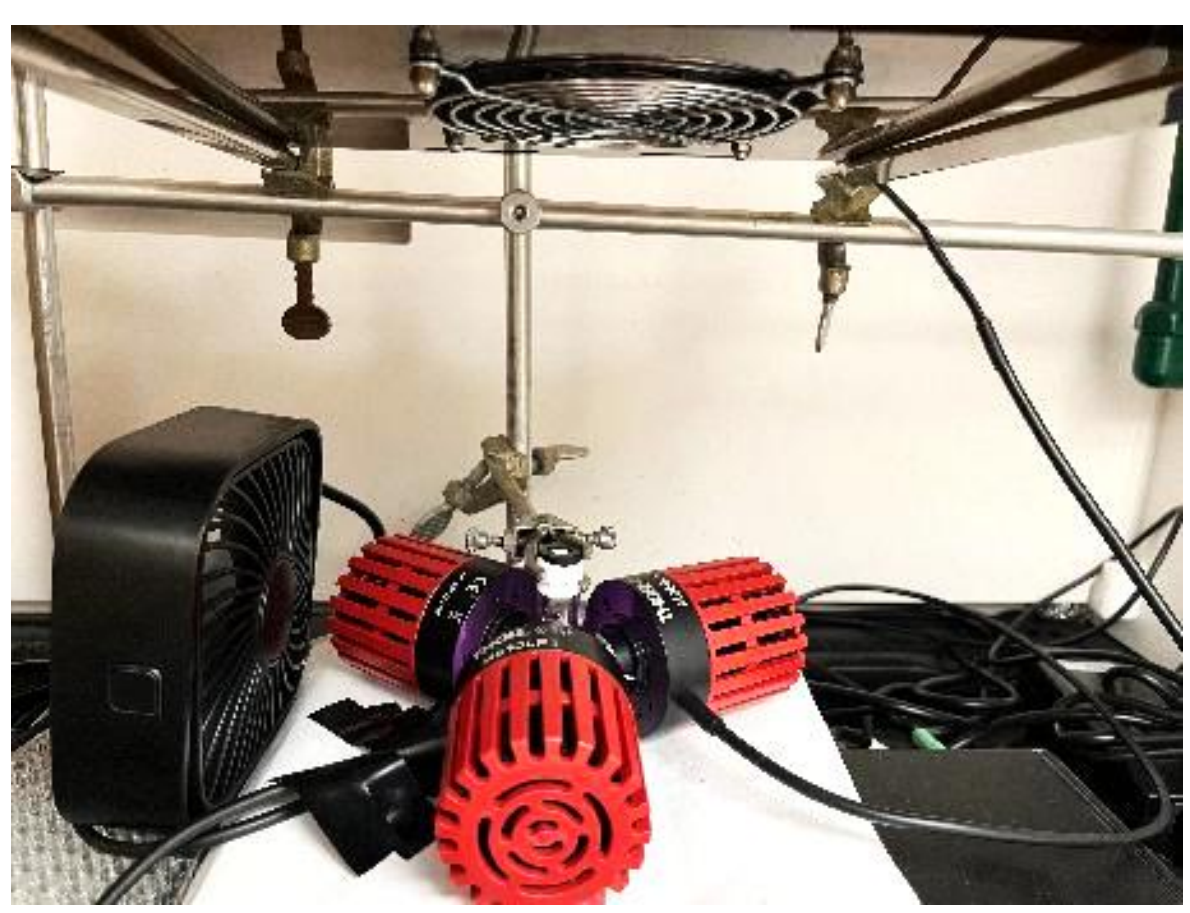

Figure S15. The experimental setup for photocatalytic reactions upon irradiation at 850 nm LEDs.

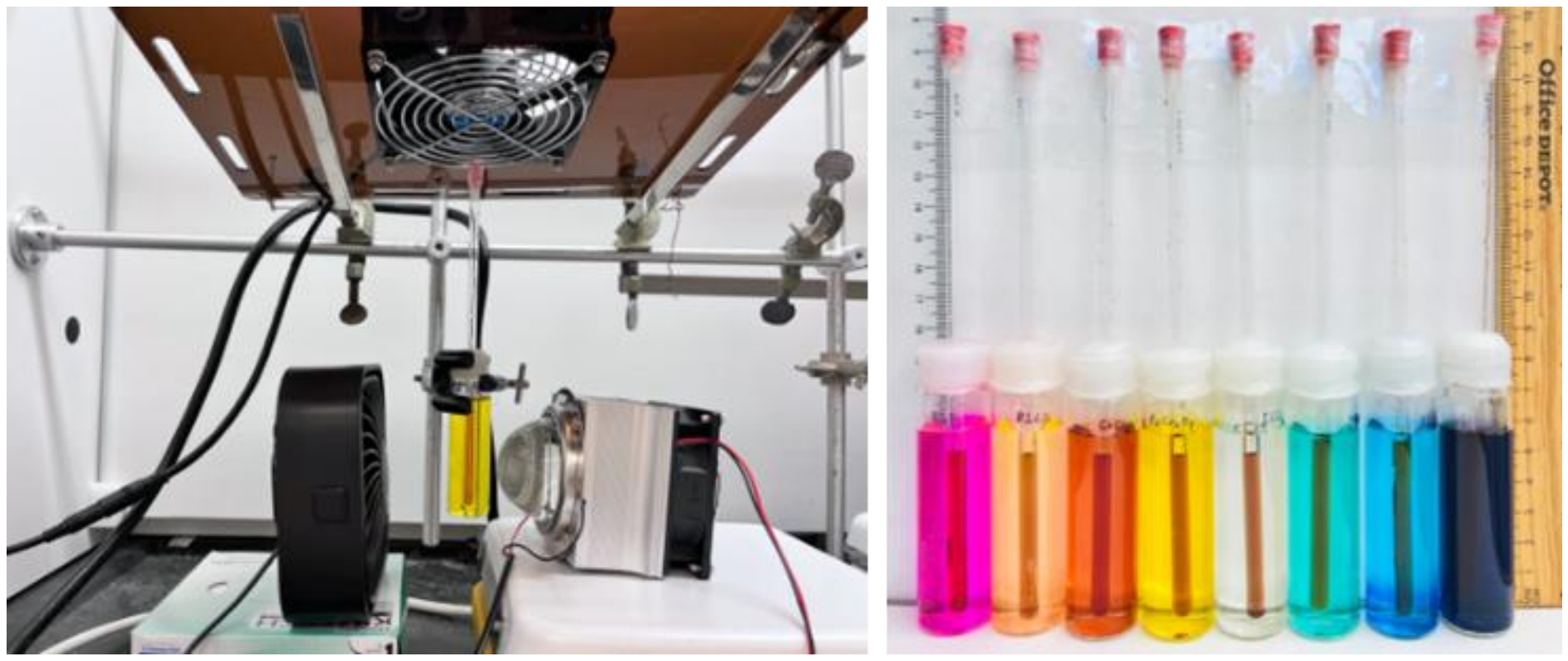

Figure S16. The experimental setup for polymerization reactions upon irradiation at 850 nm LED employing optical filter solutions.

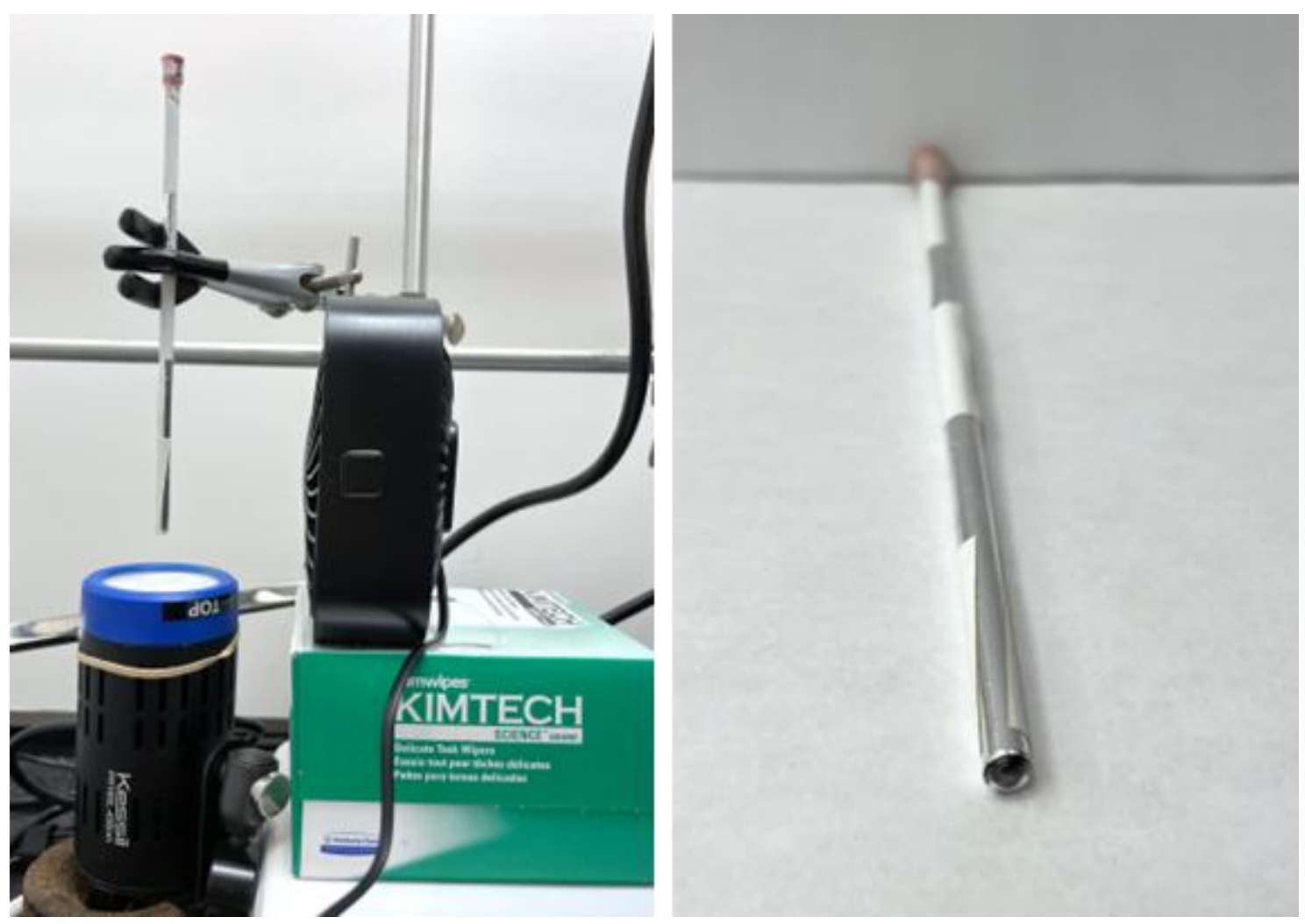

Figure S17. The experimental setup for polymerization reactions upon irradiation at different wavelengths.

7. Computational Details

All density functional theory (DFT) calculations were performed using Gaussian 16, Revision C.02.[3] Geometry optimizations were carried out at the B3LYP level of theory.[4-6] The LANL2DZ effective core potential was used for Ru,[7] while all other atoms were treated with the 6-311+G(d,p) basis set.[8-11] Solvent effects were included using the CPCM model with acetonitrile as the solvent. Frequency calculations confirmed all optimized structures to be local minimum. Time-dependent DFT calculations produced the lowest 50 singlet excited states, using the CPCM method with acetonitrile as the solvent. The calculated absorption spectra, electronic transition contributions, and electron density difference maps (EDDMs) were generated by GaussSum 3.075.[12] The electronic orbitals were visualized using VMD 1.9.3.[13]

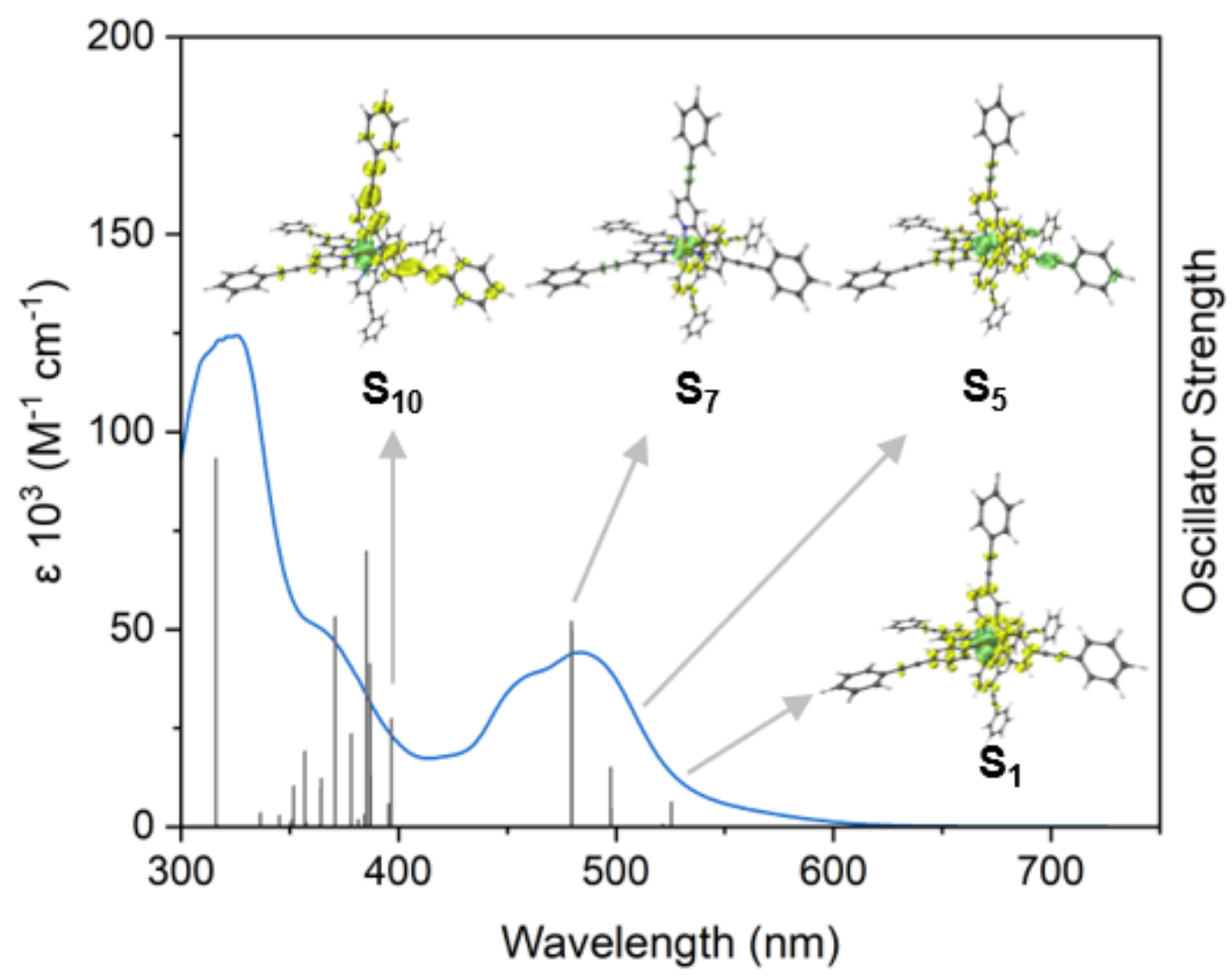

Figure S18. Comparison between the experimental absorption spectrum and the calculated singlet excited states of **Ru-bpyap** with the EDDMs of S_1, S_5, S_7 and S_{10} (isovalue = 0.04). Green and yellow indicate electron density decrease and increase, respectively.

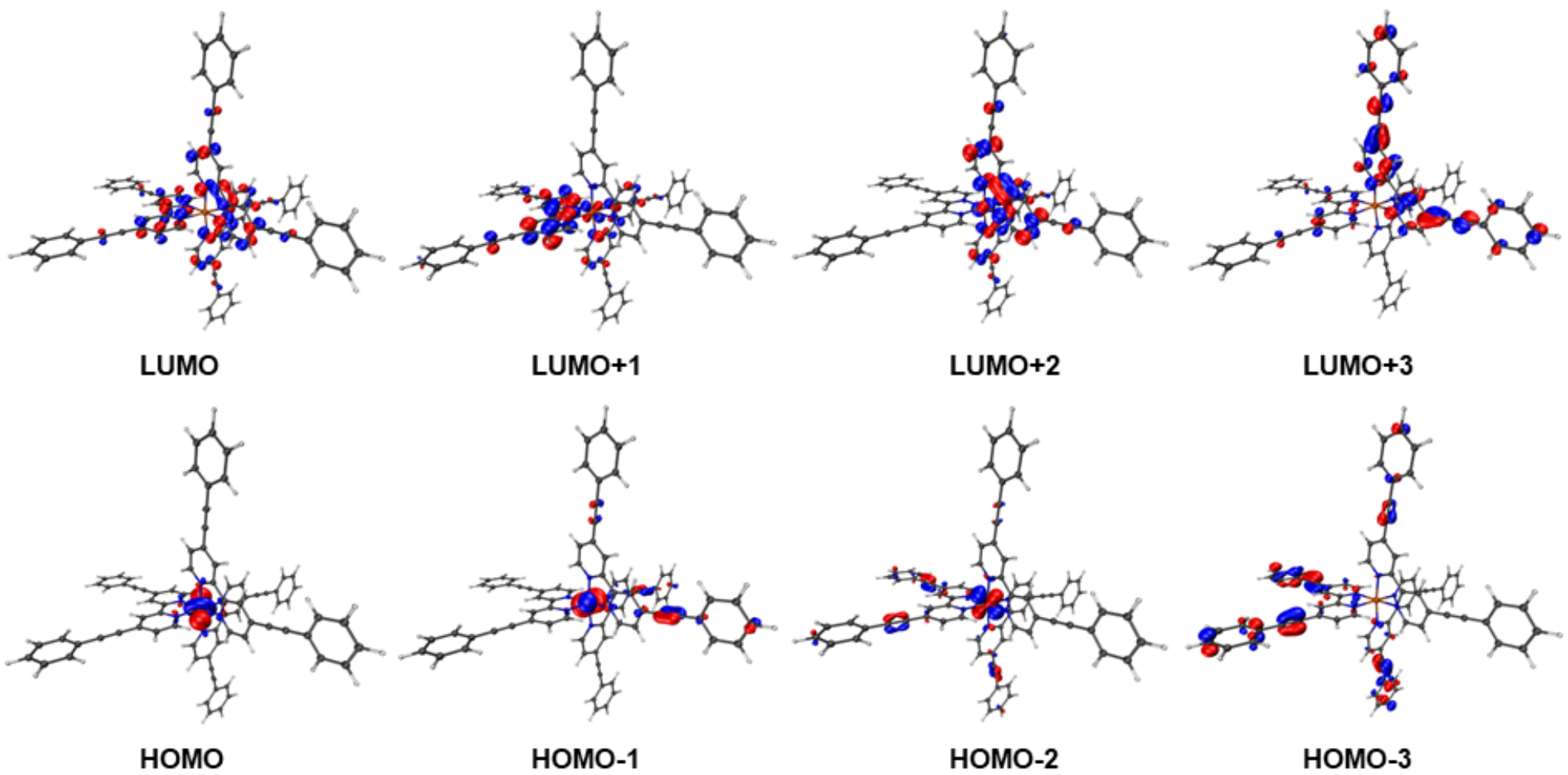

Figure S19. DFT calculated frontier molecular orbitals of **Ru-bpyap** (isovalue: 0.04).

Table S1. Calculated singlet electronic transitions of **Ru-bpyap** in CH_3CN (Isovalue = 0.04).

S_n [a]	Wavelength (nm)	f [b]	Major contribs	EDDM
S_1	525.6	0.0619	HOMO->LUMO (96%)	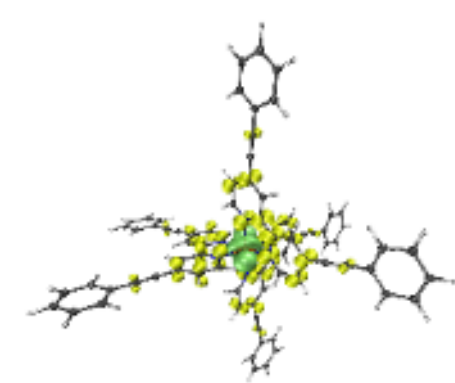
S_2	521.6	0.0058	HOMO->L+1 (94%)	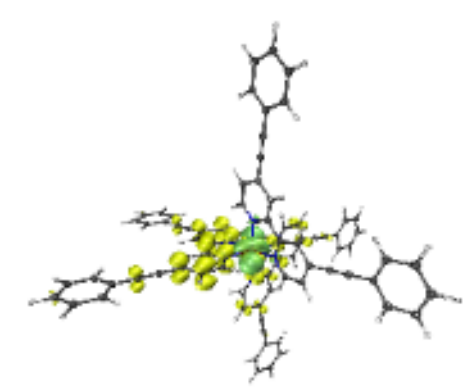
S_3	521.4	0.0052	HOMO->L+2 (94%)	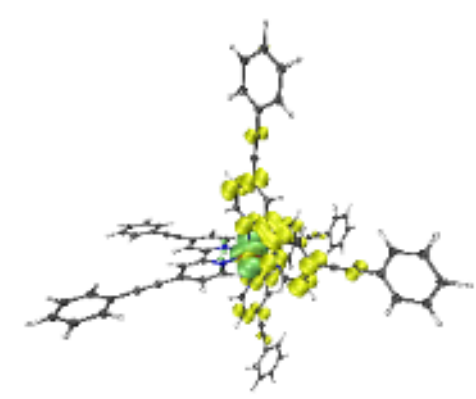

S_4	497.9	0.0464	H-2->LUMO (11%), H-2->L+2 (44%), H-1->L+1 (34%)	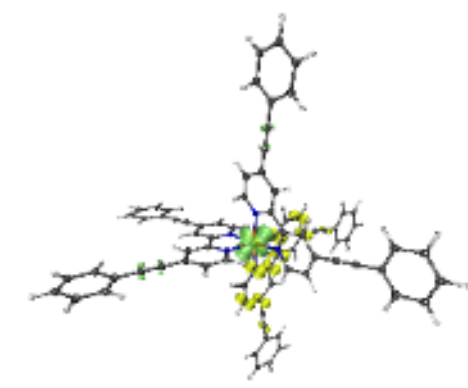
S_5	497.7	0.1499	H-1->LUMO (83%), H-1->L+1 (10%)	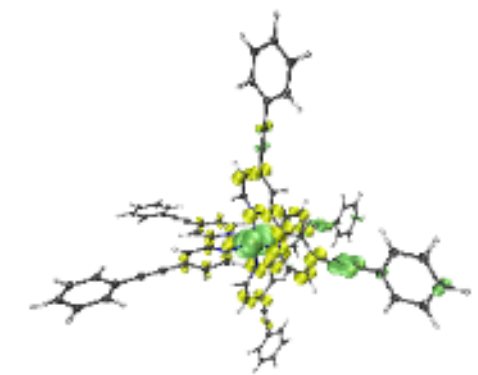
S_6	497.6	0.1434	H-2->LUMO (81%)	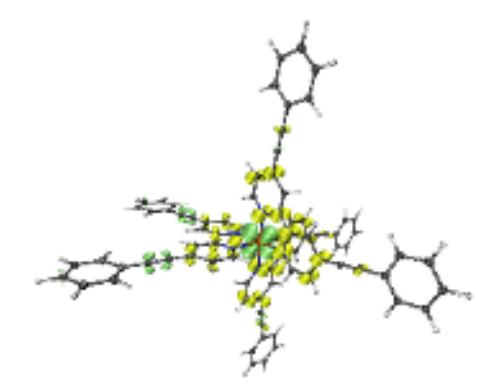
S_7	479.8	0.4965	H-2->L+1 (11%), H-2->L+2 (35%), H-1->L+1 (34%), H-1->L+2 (11%)	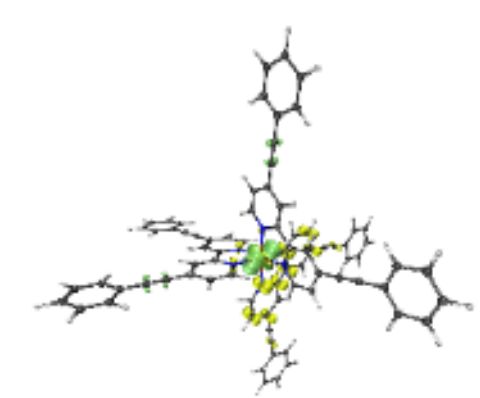
S_8	479.6	0.5194	H-2->L+1 (35%), H-2->L+2 (11%), H-1->L+1 (11%), H-1->L+2 (34%)	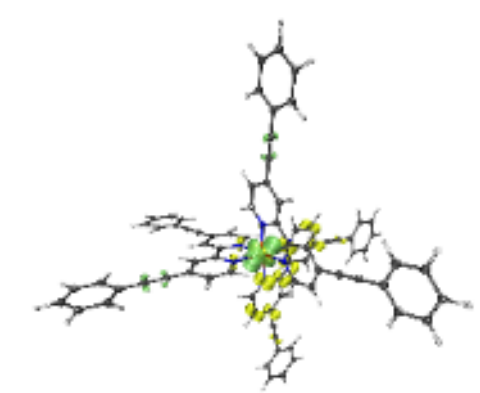
S_9	441.2	0.0000	H-2->L+1 (36%), H-1->L+2 (36%), HOMO->L+5 (21%)	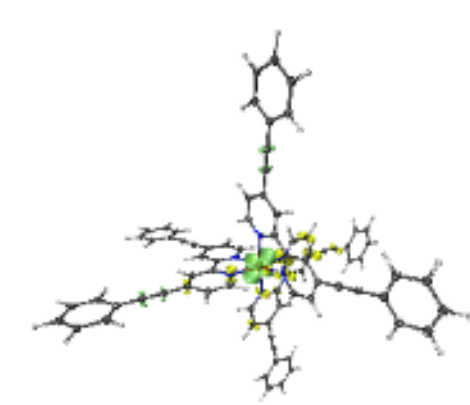
S_{10}	396.8	0.2717	HOMO->L+3 (95%)	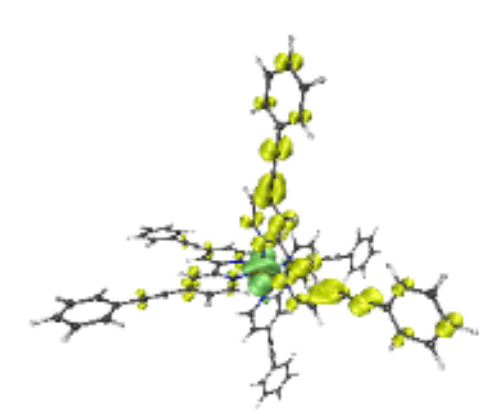

S_{11}	396.7	0.2731	HOMO->L+4 (95%)	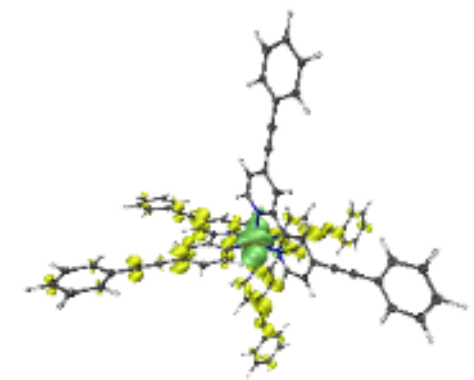
S_{12}	395.5	0.0576	HOMO->L+6 (97%)	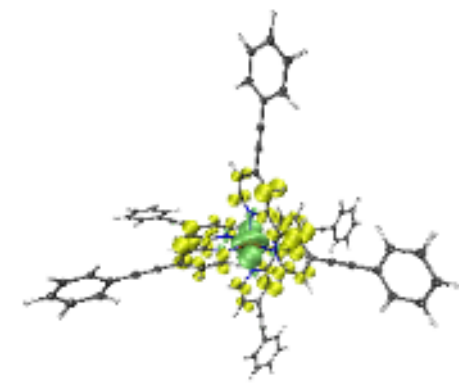
S_{13}	387.3	0.1281	H-2->L+3 (12%), H-1->L+4 (22%), H-1->L+5 (22%), HOMO->L+5 (26%)	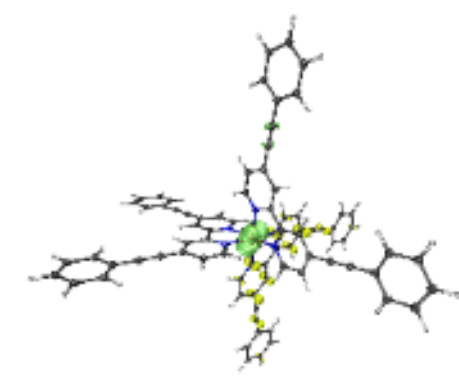
S_{14}	387.1	0.3088	H-2->L+3 (10%), H-1->L+5 (64%)	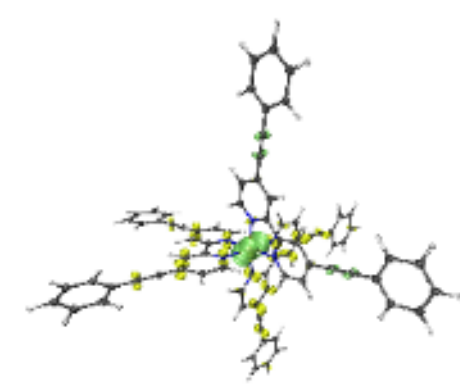
S_{15}	386.9	0.4130	H-2->L+5 (87%)	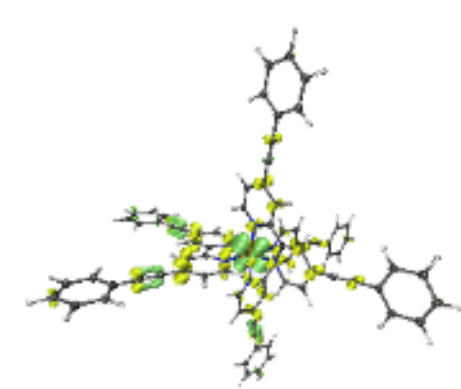
S_{16}	385.3	0.6975	H-2->L+4 (31%), H-1->L+3 (46%), H-1->L+4 (10%)	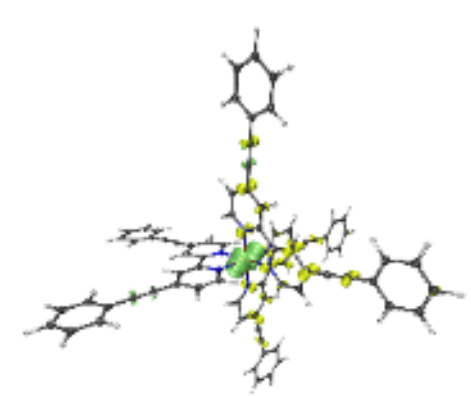
S_{17}	384.5	0.0287	H-2->L+3 (38%), H-2->L+4 (10%), H-1->L+4 (32%)	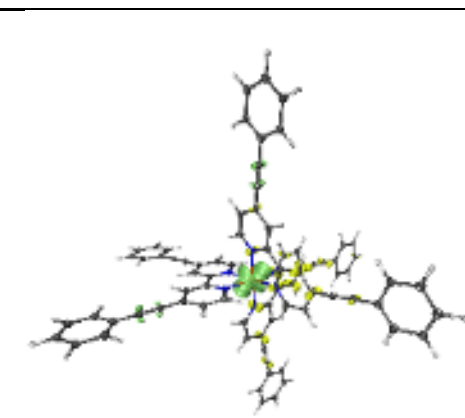

S_{18}	384.4	0.0298	H-2->L+4 (40%), H-1->L+3 (28%)	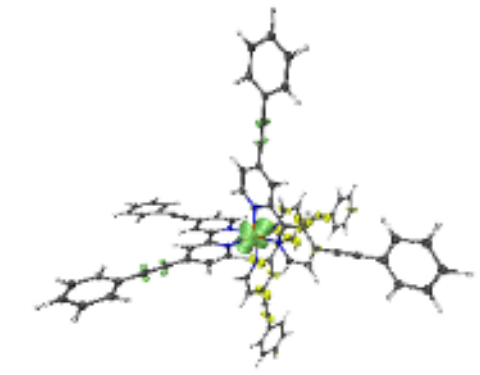
S_{19}	381.6	0.0160	H-1->L+6 (87%)	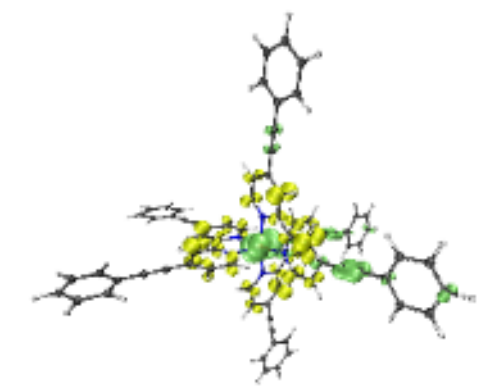
S_{20}	381.5	0.0181	H-2->L+6 (87%)	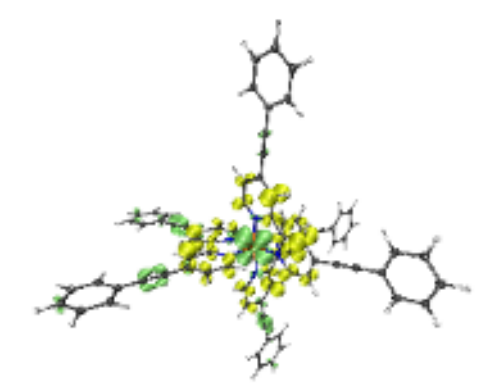

[a] Singlet excited state, [b] oscillating strength, ‘H’ and ‘L’ stand for HOMO and LUMO.

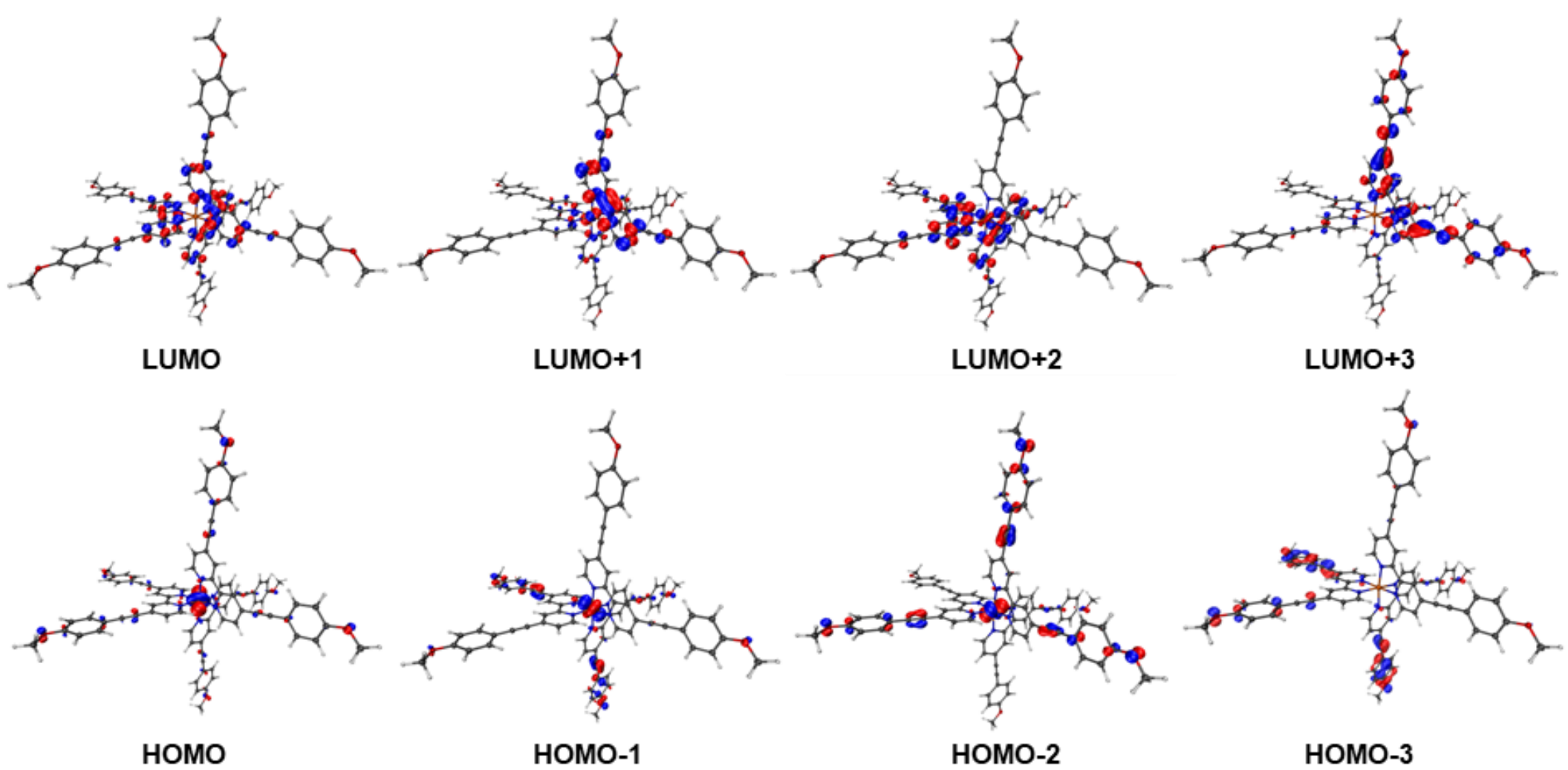

Figure S20. DFT calculated frontier molecular orbitals of **Ru-bpyap-OMe** (isovalue: 0.04).

Table S2. Calculated singlet electronic transitions of **Ru-bpyap-OMe** in CH_3CN (Isovalue = 0.04).

S_n [a]	Wavelength (nm)	f [b]	Major contribs	EDDM

S_1	531.7	0.1011	HOMO->LUMO (90%)	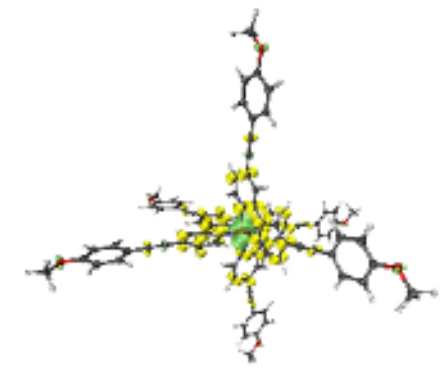
S_2	526.2	0.0073	H-6->L+1 (12%), HOMO->L+1 (86%)	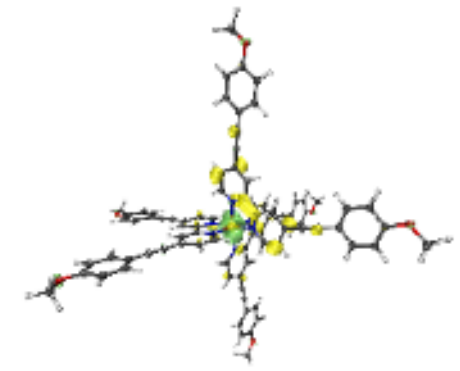
S_3	526.2	0.0071	H-6->L+2 (12%), HOMO->L+2 (86%)	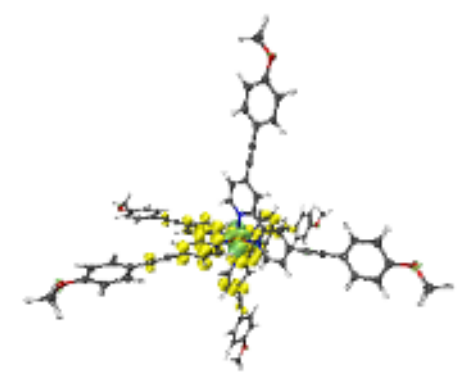
S_4	510.6	0.2681	H-1->LUMO (91%)	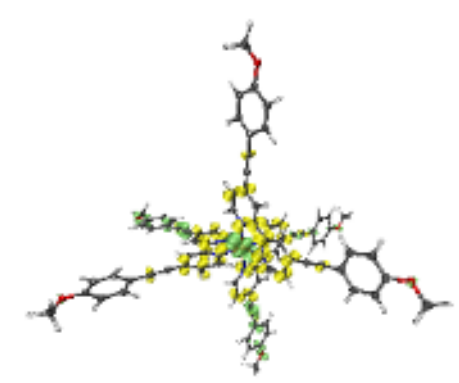
S_5	510.6	0.2705	H-2->LUMO (91%)	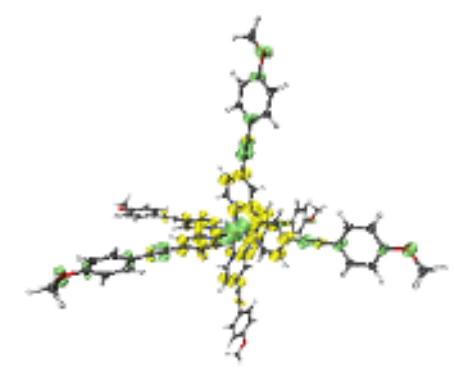
S_6	507.2	0.0333	H-2->L+2 (39%), H-1->L+1 (39%)	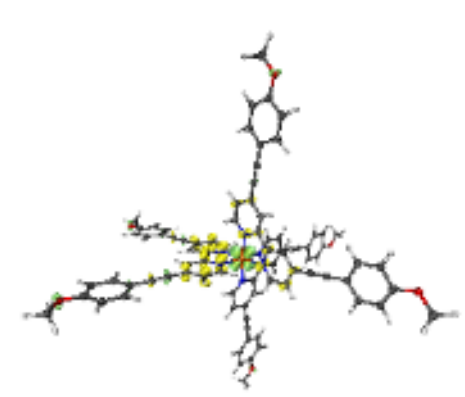
S_7	492.4	0.5545	H-2->L+1 (25%), H-2->L+2 (19%), H-1->L+1 (19%), H-1->L+2 (26%)	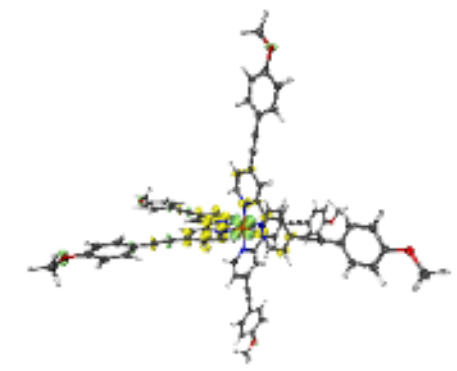

S_8	492.4	0.5573	H-2->L+1 (20%), H-2->L+2 (26%), H-1->L+1 (25%), H-1->L+2 (19%)	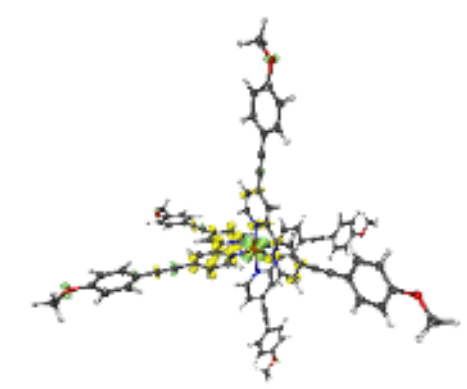
S_9	453.2	0.0000	H-2->L+1 (35%), H-1->L+2 (35%), HOMO->L+5 (12%)	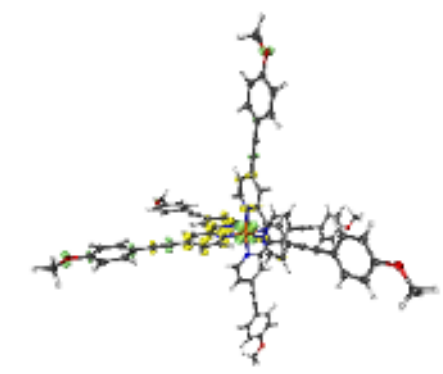
S_{10}	423.6	0.2952	H-3->LUMO (78%)	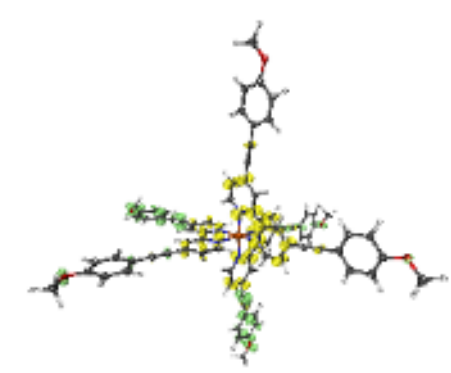
S_{11}	423.6	0.2950	H-4->LUMO (79%)	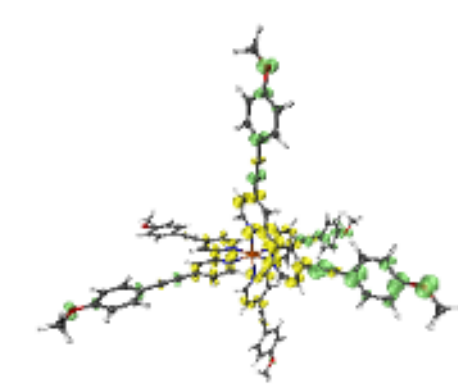
S_{12}	413.2	0.0002	H-5->LUMO (93%)	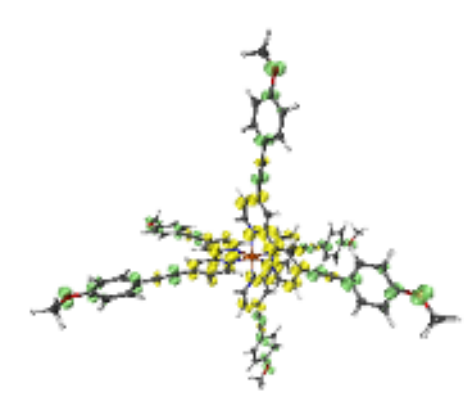
S_{13}	413.2	0.7635	H-6->LUMO (20%), H-4->L+1 (37%), H-3->L+2 (37%)	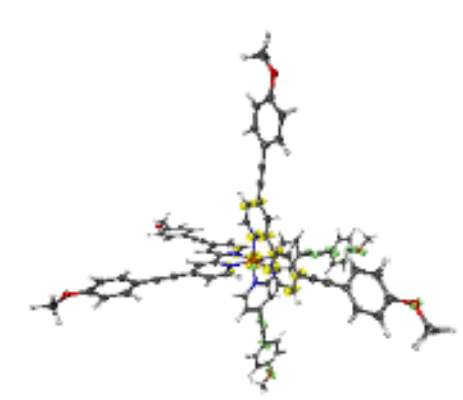
S_{14}	406.2	0.0445	H-5->L+1 (17%), H-4->L+2 (31%), H-3->L+1 (32%)	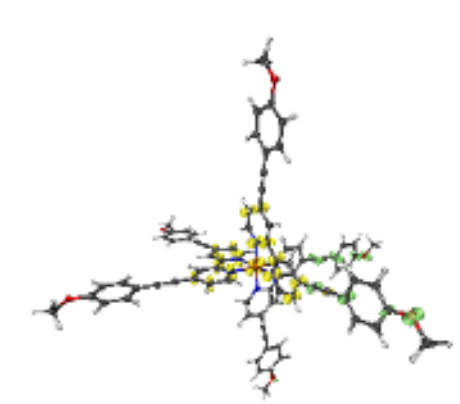

S_{15}	406.1	0.0454	H-5->L+2 (17%), H-4->L+1 (32%), H-3->L+2 (32%)	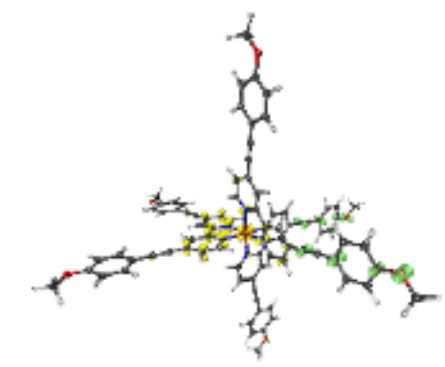
S_{16}	403.0	0.1609	HOMO->L+6 (86%)	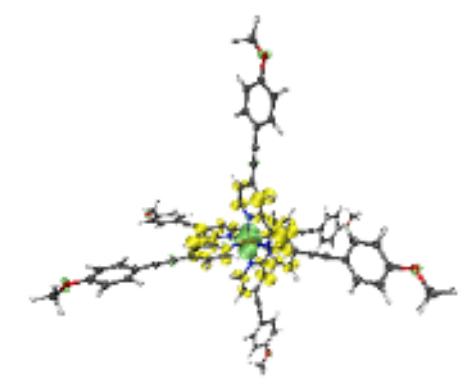
S_{17}	400.5	0.0013	H-4->L+2 (44%), H-3->L+1 (46%)	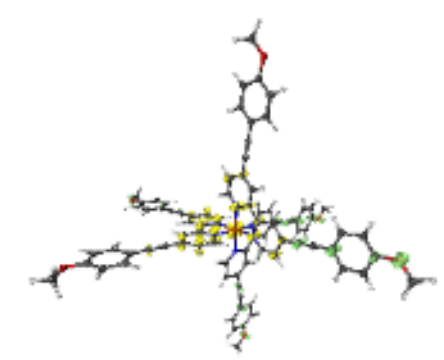
S_{18}	399.9	0.5547	H-5->L+1 (28%), HOMO->L+3 (54%)	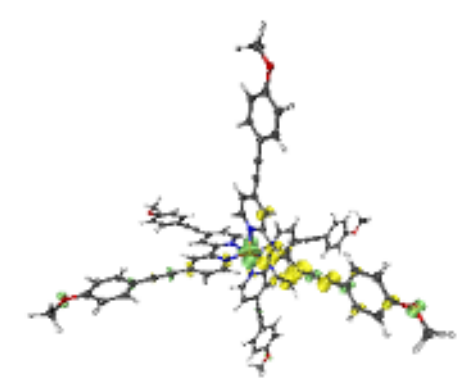
S_{19}	399.8	0.5541	H-5->L+2 (29%), HOMO->L+4 (54%)	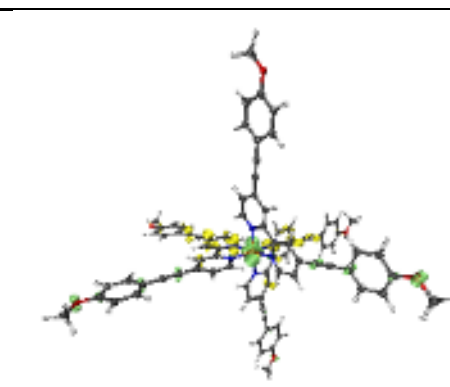
S_{20}	398.8	0.0038	H-6->LUMO (64%), H-4->L+1 (11%), H-3->L+2 (12%)	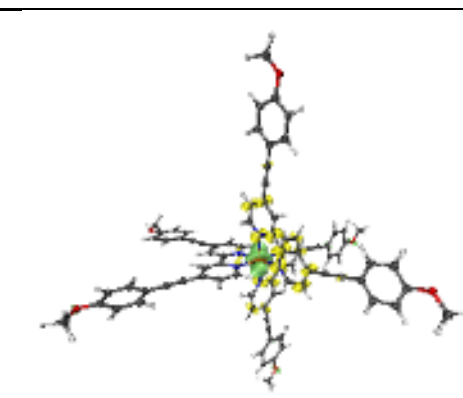

[a] Singlet excited state, [b] oscillating strength, 'H' and 'L' stand for HOMO and LUMO.

8. Photocatalysis using Ru-bpyap-OMe

The yield change over time upon irradiation at different wavelengths

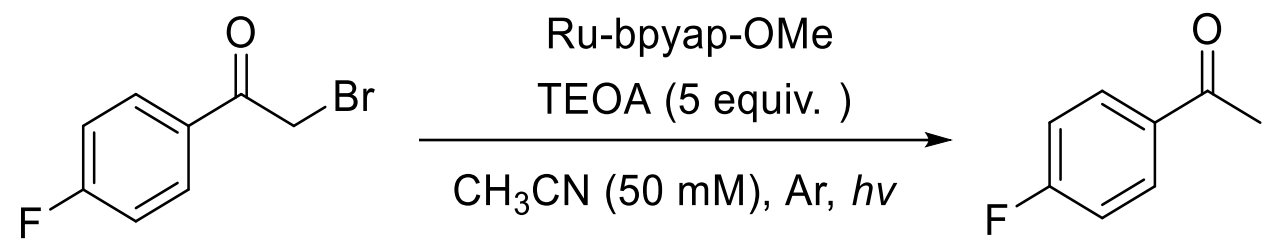

To a 20-mL vial equipped with a magnetic stir bar were added 2-bromo-4′-fluoroacetophenone (0.20 mmol, 1.0 equiv), triethanolamine (1.0 mmol, 5.0 equiv), Ru-bpyap-OMe (0.01, 2, or 10 mol% for irradiation at 525, 660, or 740 nm, respectively), and acetonitrile (4.0 mL). The resulting solution was degassed by bubbling argon for 15 min in dark and then maintained under an argon atmosphere via an argon-filled balloon. The reaction mixture was stirred at room temperature under irradiation at the indicated wavelengths. At specified time intervals, an aliquot (200 μL) of the reaction mixture was withdrawn and transferred to an NMR tube containing acetonitrile.

The product yield determined by ^{19}F NMR was calculated from the relative integrations of characteristic peaks as equation:

$$\text{Yield (\%)} = \left(\frac{I_{\text{prd.}}}{I_{\text{prd.}} + I_{\text{SM}}}\right) \times 100\%$$

The entire procedure was repeated with the LED power set to 75%, 50%, and 25% (for 525 and 660 nm irradiation) and with different numbers of lamps for 740 nm irradiation.

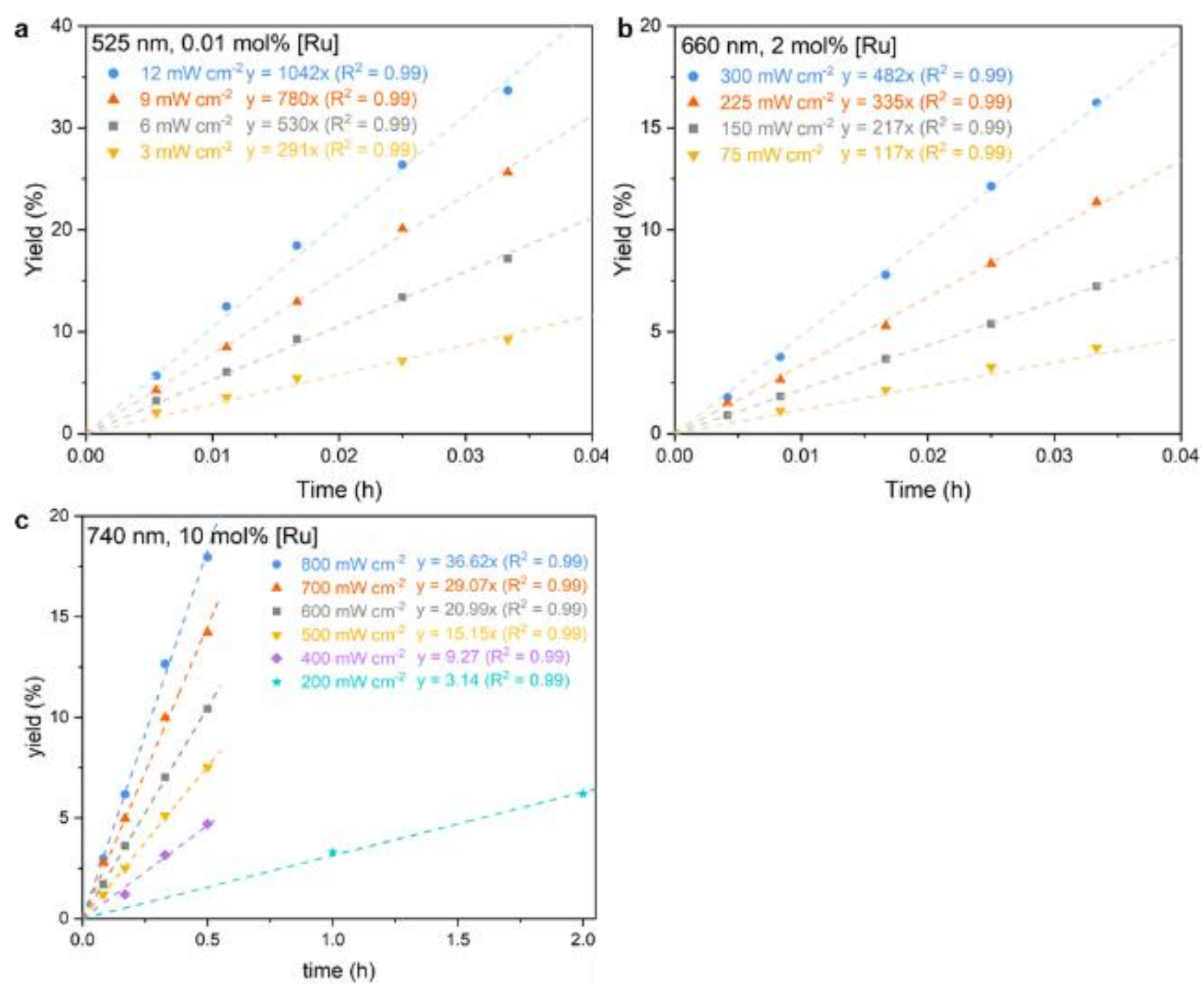

Figure S21. The change in yield over time upon irradiation of different power.

For the reaction employing a solution of 2-bromo-4´-fluoroacetophenone (43.4 mg, 0.20 mmol), triethanolamine (149 mg, 1.0 mmol), Ru-bpyap-OMe (0.01 mol%, 40 μL of 0.5 mM stock solution) in CH_3CN (4 mL) under irradiation with 525 nm light for 21 min. After completion of the reaction, the solvent was removed under reduced pressure. The residue was purified by short silica gel column chromatography using hexane/ethyl acetate (50:1) as the eluent to afford 4-fluoroacetophenone as colorless liquid (21.5 mg, 78% isolated yield).

^{1}H NMR (500 MHz, $CDCl_3$) δ 8.01 – 7.95 (m, 2H), 7.12 (t, J = 8.6 Hz, 2H), 2.58 (s, 3H) ppm.

^{13}C{^{1}H} NMR (126 MHz, $CDCl_3$) δ 196.61, 165.88 (d, J = 254.6 Hz), 133.69 (d, J = 3.1 Hz), 131.06 (d, J = 9.4 Hz), 115.77 (d, J = 21.8 Hz), 26.66 ppm.

^{19}F NMR (376 MHz, $CDCl_3$) δ -107.93 (ddd, J = 13.9, 8.7, 5.6 Hz) ppm.

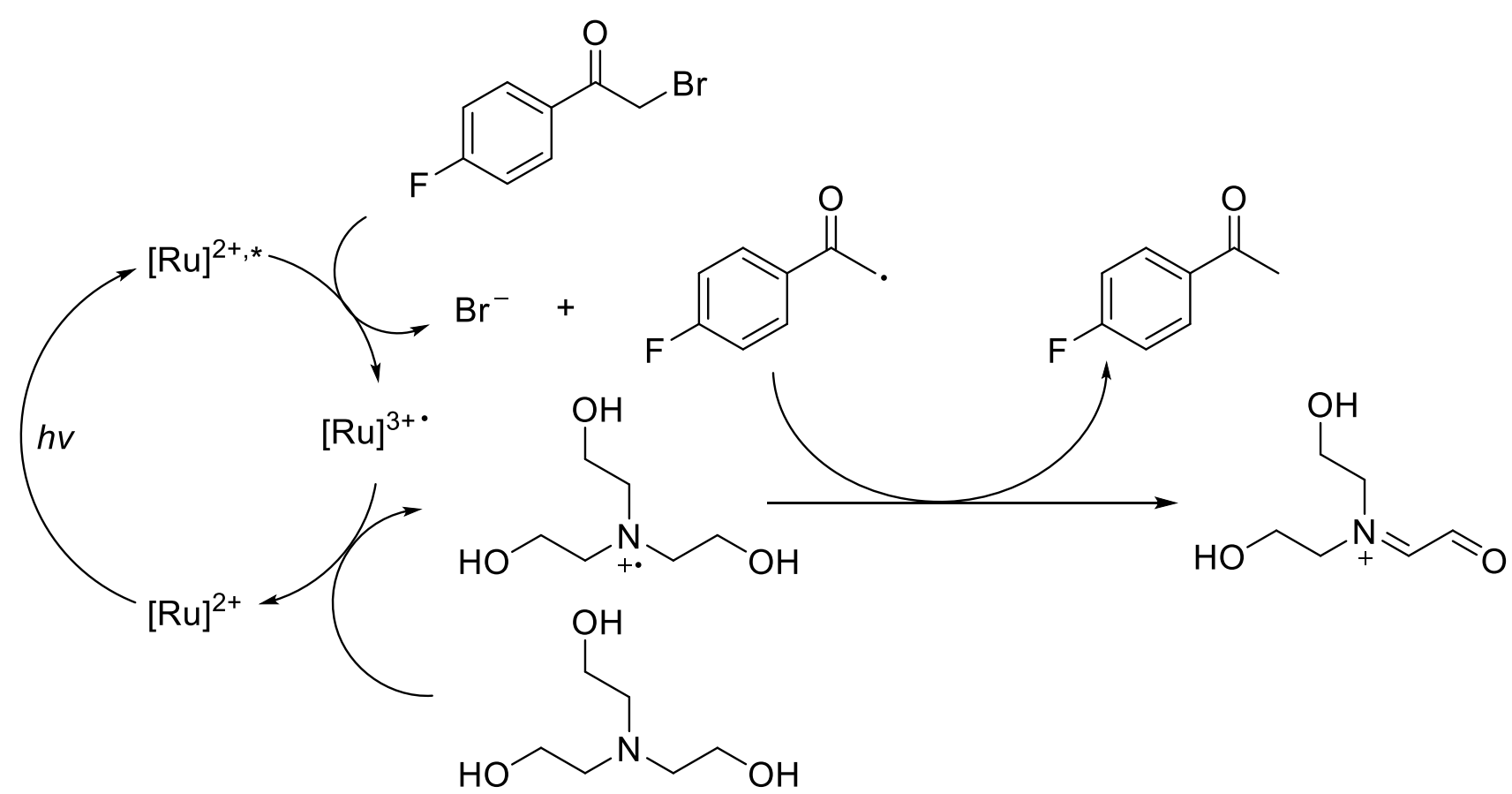

Scheme S1. Proposed mechanism of photocatalytic hydrodebromination.

Photocatalytic trifluoromethylation of 2-methylfuran

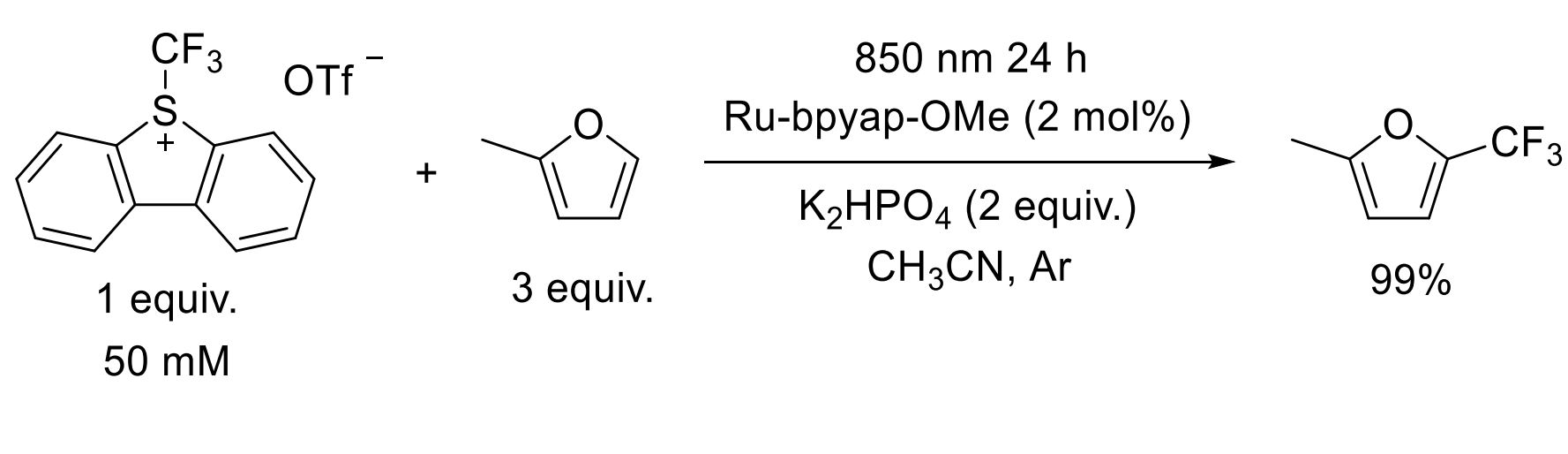

A solution of 5-(trifluoromethyl)dibenzothiophenium trifluoromethanesulfonate (40.2 mg, 0.1 mmol), 2-methylfuran (26.5 μL, 0.3 mmol), dipotassium phosphate (34.8 mg, 0.2 mmol), and Ru-bpyap-OMe (3.3 mg, 2 mol%) in CD_3CN (2 mL) was degassed by bubbling with Ar for 15 min in the dark. The resulting mixture was stirred at room temperature under irradiation with 850 nm light for 24 h. After completion of the reaction, an aliquot (500 μL)

of the reaction mixture was withdrawn and transferred to an NMR tube, and full conversion was confirmed by ^{1}H NMR and ^{19}F NMR analyses of the crude reaction mixture (**Fig. S45–S46**). Due to the low boiling point of the product (81 °C), isolation and purification proved challenging. Nevertheless, the formation of the 5-trifluoromethylation-2-methylfuran was confirmed by high-resolution mass spectrometry (HRMS) of the crude reaction mixture.

LC-MS (ESI-positive mode) calc. for $[(C_6H_5F_3O)H]^+$ m/z: 151.0365. Found: 151.0368.

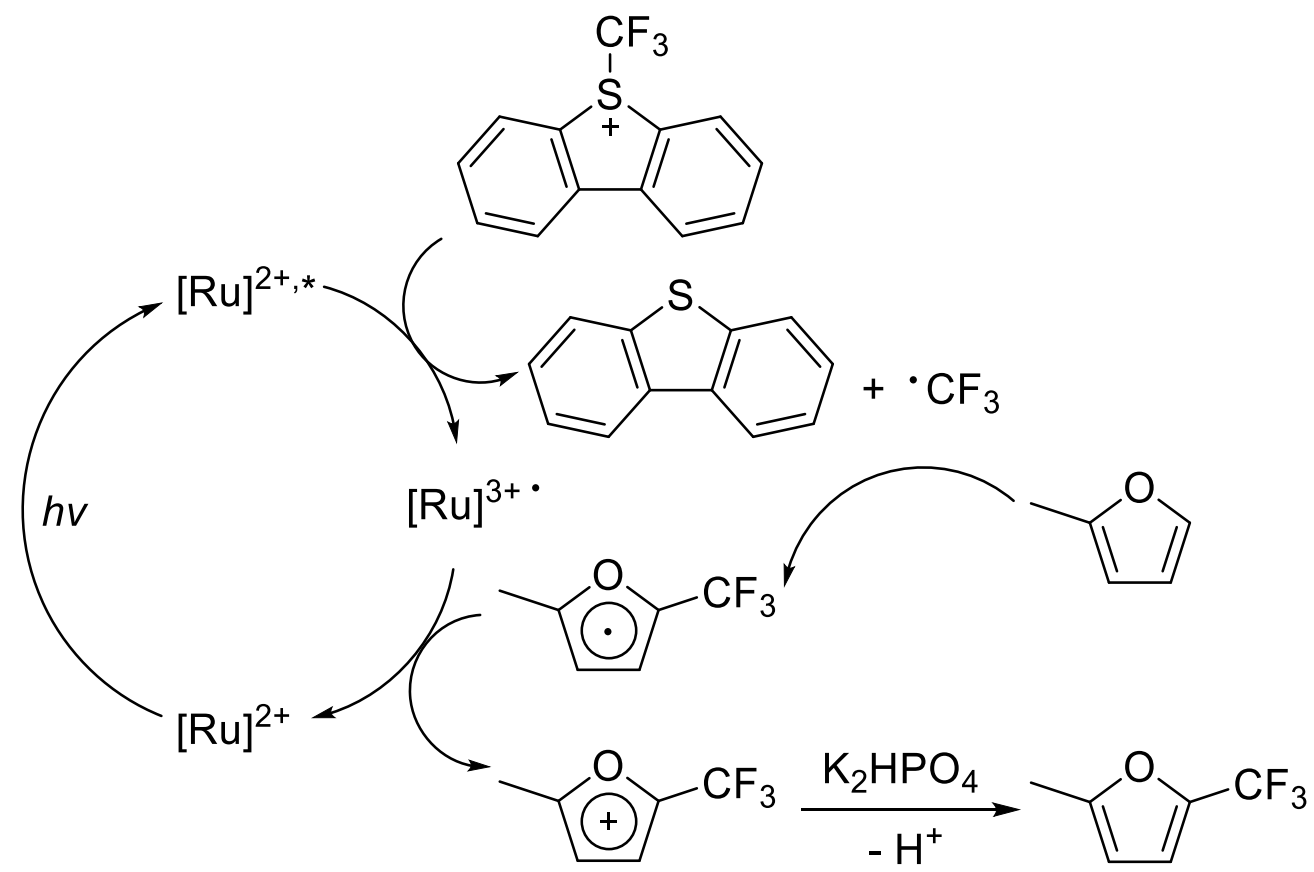

Scheme S2. Proposed mechanism of photocatalytic trifluoromethylation.

Photocatalytic phenylation of 1-methyl-1H-pyrrole

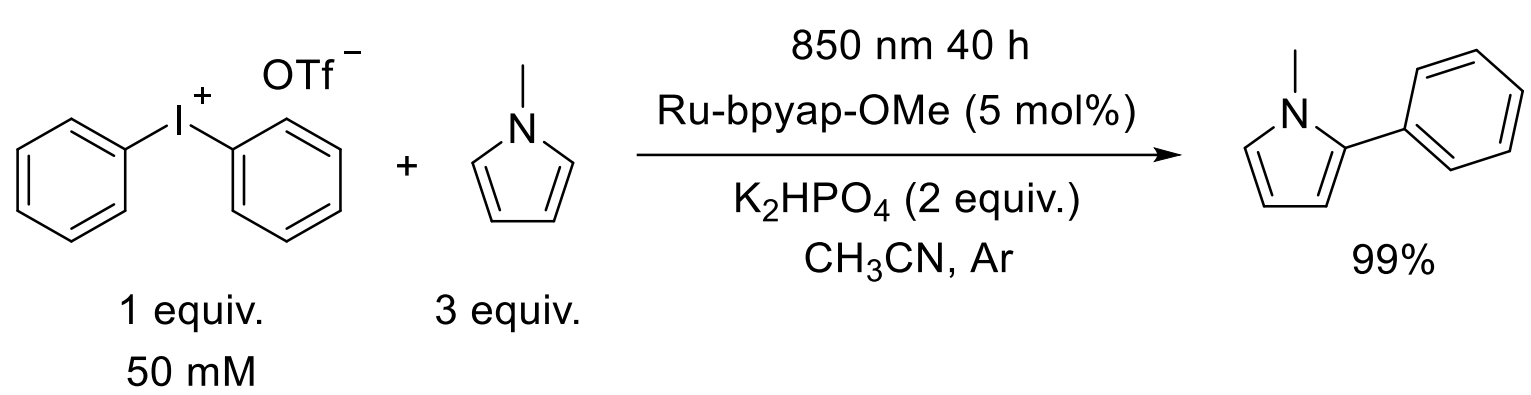

A solution of diphenyliodonium triflate (43.0 mg, 0.1 mmol), 1-methyl-1H-pyrrole (25.8 μL, 0.3 mmol), dipotassium phosphate (34.8 mg, 0.2 mmol), and Ru-bpyap-OMe (8.2 mg, 5 mol%) in CH_3CN (2 mL) was degassed by bubbling with Ar for 15 min in the dark. The resulting mixture was stirred at room temperature under irradiation with 850 nm light for 40 h. After completion of the reaction, the solvent was removed under reduced pressure, and full conversion was confirmed by ^{1}H NMR analysis of the crude reaction mixture. The residue was purified by short silica gel column chromatography using hexane with 3% triethylamine as the eluent to afford 1-methyl-2-phenyl-1H-pyrrole as a colorless solid (9 mg, 57% isolated yield).

^{1}H NMR (500 MHz, $CDCl_3$) δ 7.47 – 7.39 (m, 4H), 7.31 (dt, J = 6.0, 2.9 Hz, 1H), 6.73 (t, J = 2.2 Hz, 1H), 6.28 – 6.17 (m, 2H), 3.68 (s, 3H) ppm.

^{13}C{^{1}H} NMR (126 MHz, $CDCl_3$) δ 134.75, 133.48, 128.79, 128.48, 126.86, 123.77,

108.76, 107.89, 35.20 ppm.

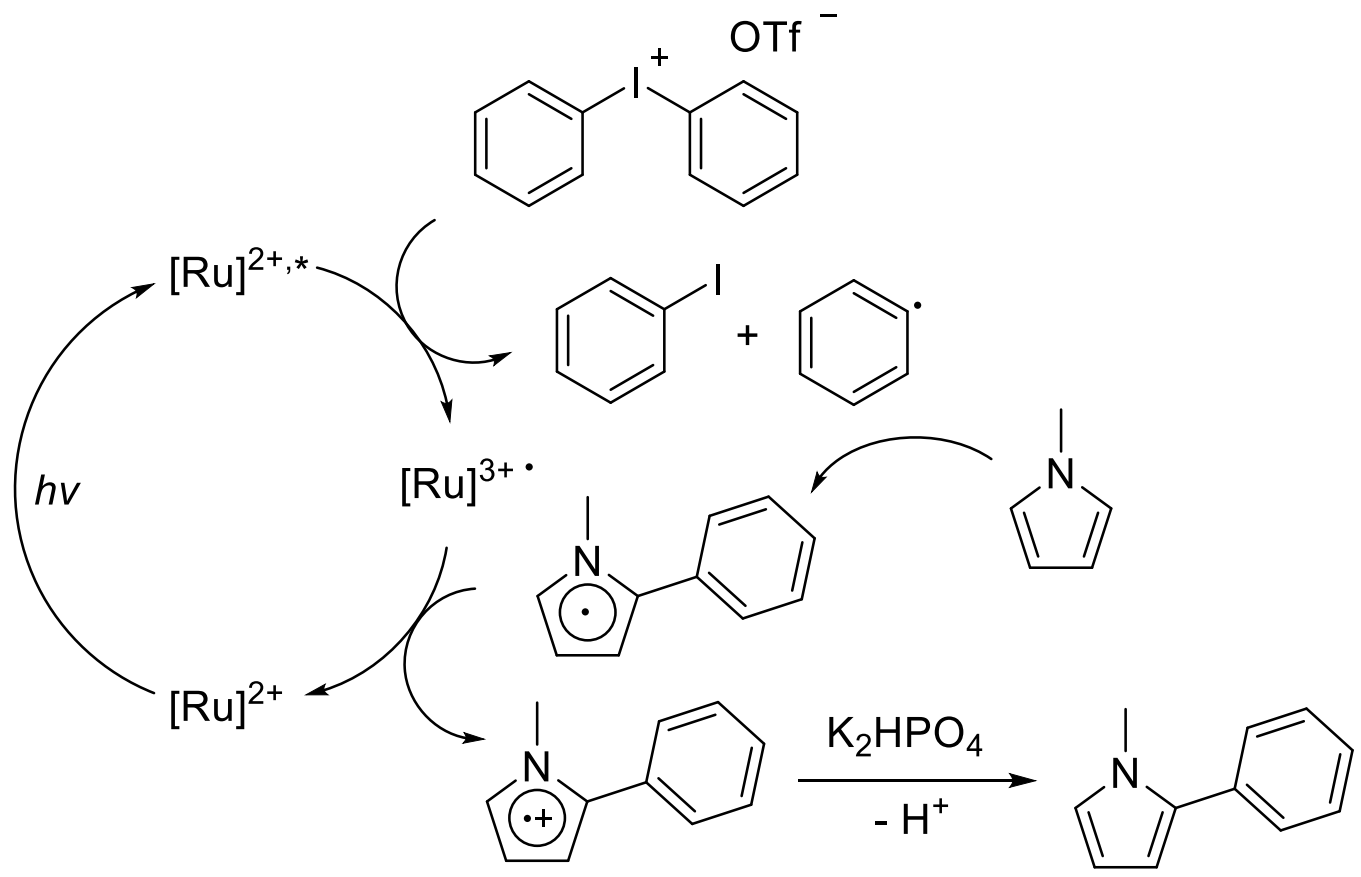

Scheme S3. Proposed mechanism of photocatalytic phenylation.

Synthesis of 2-(4-methoxyphenyl)-1,2,3,4-tetrahydroisoquinoline

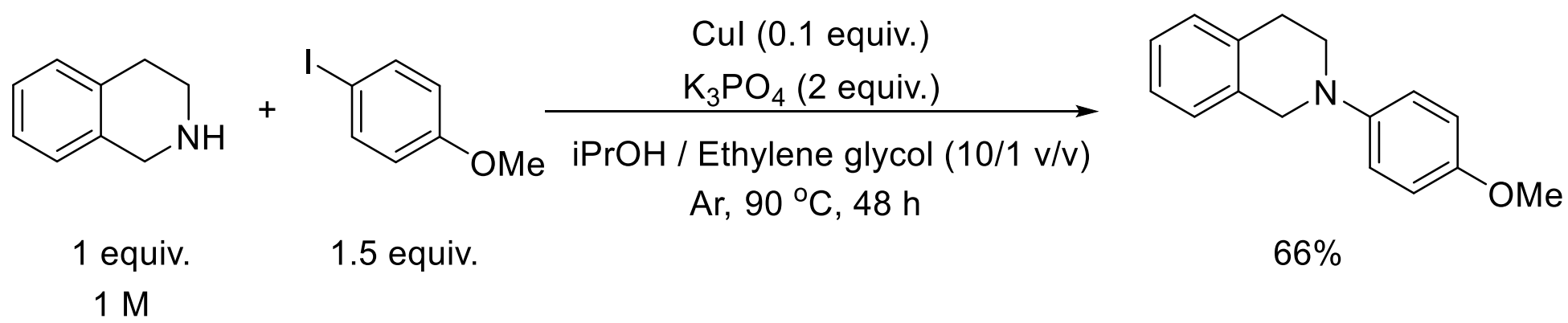

A mixture of 1,2,3,4-tetrahydro-isoquinoline (1.25 mL, 10.0 mmol), 4-iodoanisole (3.51 g, 15.0 mmol), copper(I) iodide (0.19 g, 1.0 mmol) and potassium phosphate (4.24 g, 20.0 mmol) in 2-propanol (10 mL) and ethylene glycol (1 mL) was heated at 90 °C in argon atmosphere for 48 h and then allowed to cool to room temperature. Diethyl ether (30 mL) and water (30 mL) were then added to the reaction mixture. The organic layer was extracted by diethyl ether (3 × 30 mL). The combined organic phases were washed with brine and dried over magnesium sulfate. The product was purified by chromatography on a silica gel column using hexane/ethyl acetate (20:1) as eluent to give 2-(4-methoxyphenyl)-1,2,3,4-tetrahydroisoquinoline as a white solid (1.58 g, 66% yield).

^{1}H NMR (500 MHz, $CDCl_3$) δ 7.19 – 7.11 (m, 4H), 7.01 – 6.96 (m, 2H), 6.90 – 6.84 (m, 2H), 4.30 (s, 2H), 3.78 (s, 3H), 3.45 (t, J = 5.9 Hz, 2H), 2.99 (t, J = 5.9 Hz, 2H) ppm.

^{13}C{^{1}H} NMR (126 MHz, $CDCl_3$) δ 153.62, 145.49, 134.75, 134.68, 128.83, 126.65, 126.39, 126.04, 118.17, 114.69, 55.78, 52.82, 48.59, 29.25 ppm.

Photocatalytic cross-dehydrogenative coupling (CDC) reaction

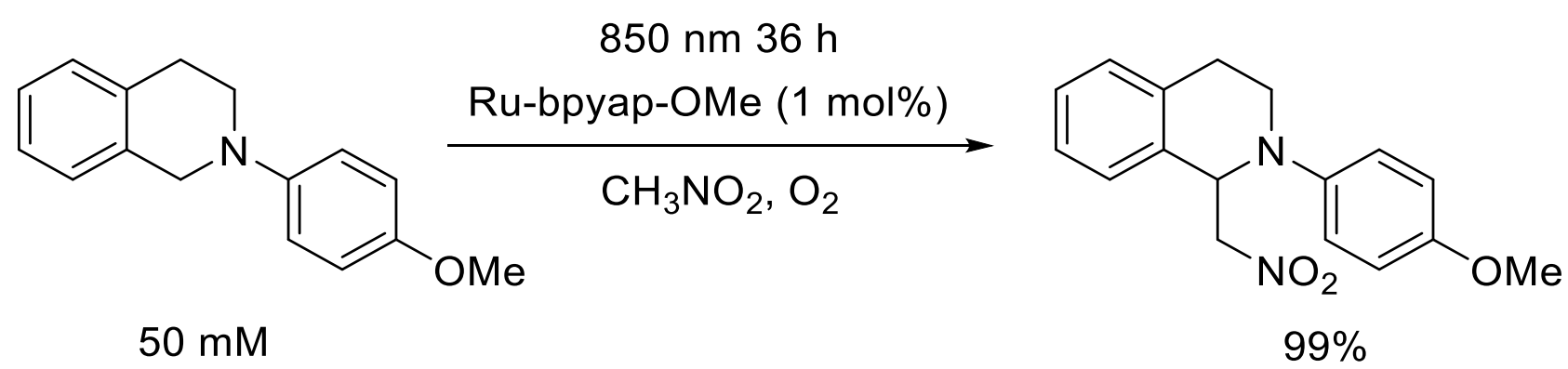

A solution of 2-(4-methoxyphenyl)-1,2,3,4-tetrahydroisoquinoline (23.9 mg, 0.1 mmol) and Ru-bpyap-OMe (0.2 mL of a 5 mM stock solution, 1 mol%) in CH_3NO_2 (2 mL) was bubbled with O_2 for 5 min in the dark. The resulting mixture was stirred at room temperature under irradiation with 850 nm light for 36 h. After completion of the reaction, the solvent was removed under reduced pressure, and full conversion was confirmed by ^{1}H NMR analysis of the crude reaction mixture. The residue was purified by short silica gel column chromatography using hexane/ethyl acetate (1:1, v/v) as the eluent to afford 1-(nitromethyl)-2-(4-methoxyphenyl)-1,2,3,4-tetrahydroisoquinoline as a yellow oil (23 mg, 76% isolated yield).

^{1}H NMR (500 MHz, $CDCl_3$) δ 7.25 – 7.19 (m, 2H), 7.18 – 7.13 (m, 2H), 6.95 – 6.89 (m, 2H), 6.85 – 6.79 (m, 2H), 5.39 (dd, *J* = 8.7, 5.8 Hz, 1H), 4.83 (dd, *J* = 11.9, 8.6 Hz, 1H), 4.56 (dd, *J* = 11.9, 5.8 Hz, 1H), 3.75 (s, 3H), 3.57 (dt, *J* = 10.0, 4.0 Hz, 2H), 3.02 (ddd, *J* = 16.3, 9.4, 6.3 Hz, 1H), 2.70 (dt, *J* = 16.5, 4.0 Hz, 1H) ppm.

^{13}C{^{1}H} NMR (126 MHz, $CDCl_3$) δ 154.09, 143.18, 135.57, 132.99, 129.59, 128.02, 127.04, 126.75, 118.98, 114.81, 79.08, 59.04, 55.71, 43.25, 25.91 ppm.

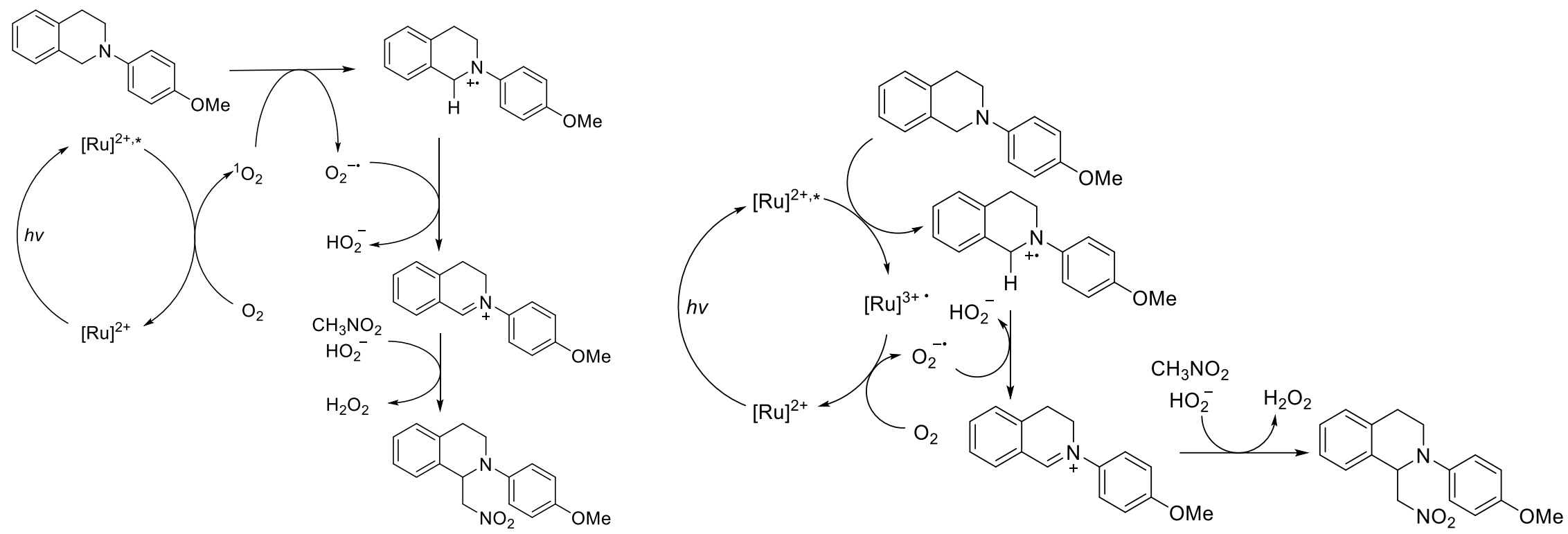

Scheme S4. Proposed mechanism of photocatalytic cross-dehydrogenative coupling (CDC) reaction via energy transfer or electron transfer.

Photocatalytic cyclocondensation to form 2-(5-thiazolyl)-1H-benzimidazole

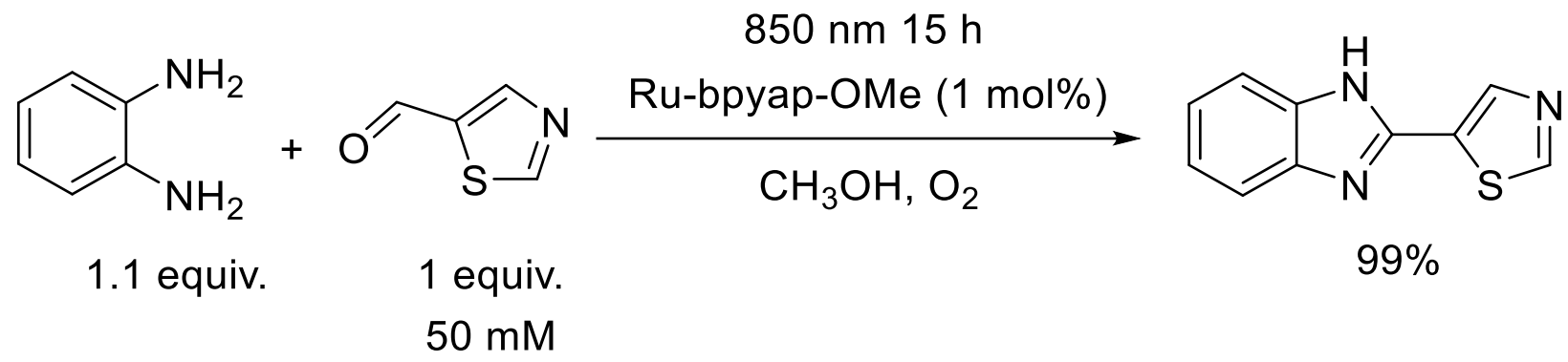

A solution of *o*-phenylenediamine (23.7 mg, 0.22 mmol), 5-thiazolecarboxaldehyde (17.3 μL, 0.2 mmol), and Ru-bpyap-OMe (3.3 mg, 1 mol%) in CH_3OH (4 mL) was stirred in the dark for 15 min and bubbled with O_2 for 5 min in the dark. The resulting mixture was stirred at room temperature under irradiation with 850 nm light for 15 h. After completion of the reaction, the solvent was removed under reduced pressure, and full conversion was confirmed by ^{1}H NMR analysis of the crude reaction mixture. The residue was purified by short silica gel column chromatography using hexane/ethyl acetate (1:3) as the eluent to afford 2-(5-thiazolyl)-1H-benzimidazole as a pale-yellow solid (24 mg, 60% isolated yield).

^{1}H NMR (500 MHz, DMSO) δ 13.16 (s, 1H), 9.23 (s, 1H), 8.58 (s, 1H), 7.64 (d, *J* = 7.9 Hz, 1H), 7.55 (d, *J* = 7.9 Hz, 1H), 7.23 (dd, *J* = 17.2, 7.7 Hz, 2H) ppm.

^{13}C{^{1}H} NMR (126 MHz, DMSO) δ 156.20, 144.55, 143.57, 141.98, 134.63, 129.64, 123.18, 122.03, 118.79, 111.41 ppm.

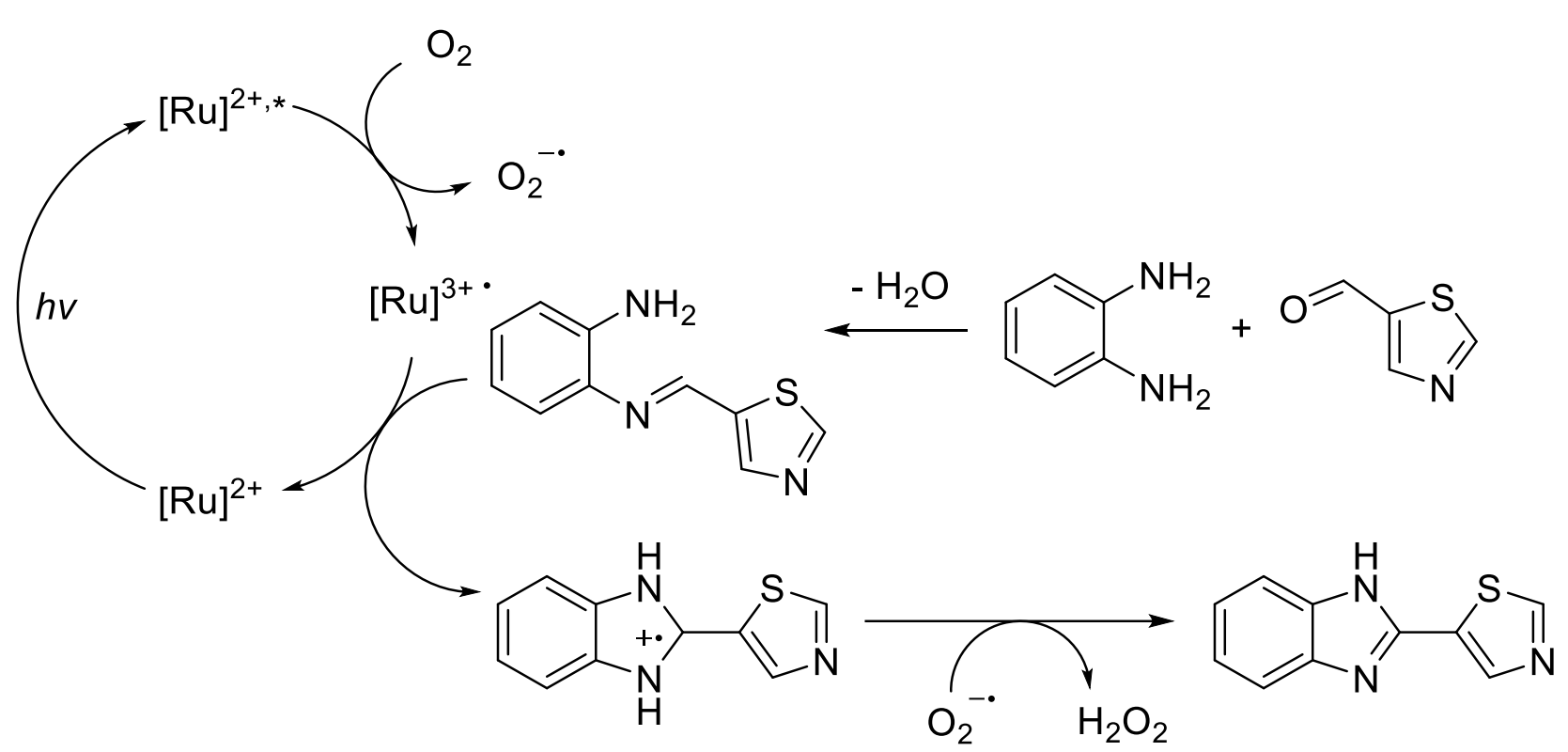

Scheme S5. Proposed mechanism of photocatalytic cyclocondensation reaction.

Photopolymerization of pentaerythritol triacrylate (PETA)

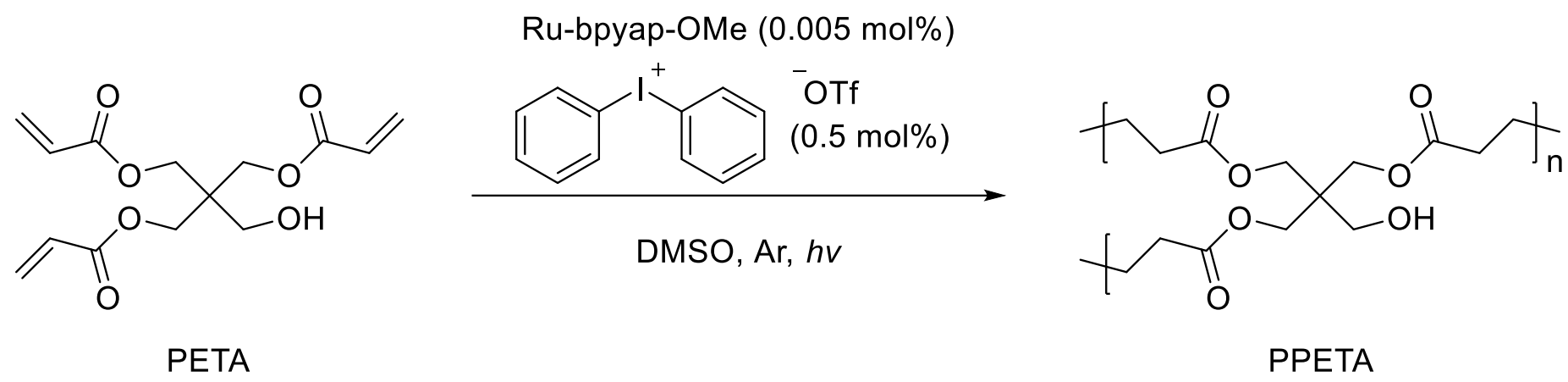

A solution of pentaerythritol triacrylate (PETA, 10 mL, 40 mmol), diphenyliodonium triflate (DPI, 86 mg, 0.5 mol%), and Ru-bpyap-OMe (0.4 mL of a 5 mM stock solution, 0.005 mol%) in DMSO (10 mL) was degassed by bubbling with Ar under stirring for 15 min in dark.

Polymerization with different optical filter solutions.

Aliquots (0.5 mL) of the reaction mixture were transferred into NMR tubes to a solution height of 50 mm and sealed under Ar. In separate 20 mL vials (22 mm diameter), selected inorganic salts ($CoCl_2$, $K_2Cr_2O_7$, $NiSO_4$, and $Cr(NO_3)_3$) and organic dyes (Rhodamine 6G, Rhodamine B, methylene blue, and indocyanine green) were dissolved in 18 mL of H_2O or DMSO, as appropriate, to serve as optical filter solutions. The tubes were then immersed in the corresponding filter solutions and irradiated with an 850 nm LED for a specified time. Upon completion, the reaction mixture formed a freestanding gel.

Table S3. Irradiation time required for polymerization with different optical filter solutions.

optical filter	$t_{irr.}$ (h)	optical filter	$t_{irr.}$ (h)
$CoCl_2$	2	Rhodamine 6G	0.67
$K_2Cr_2O_7$	2	Rhodamine B	1.5
$NiSO_4$	1	methylene blue	8
$Cr(NO_3)_3$	2	indocyanine green	2

Polymerization under irradiation at different wavelengths.

Aliquots (1.5 mL) of the reaction mixture were transferred into NMR tubes to a solution height of 150 mm and sealed under Ar. The tubes were wrapped with aluminum foil to prevent side irradiation, ensuring that light entered exclusively from the bottom. The samples were then irradiated at the desired wavelengths for 2 h.

a

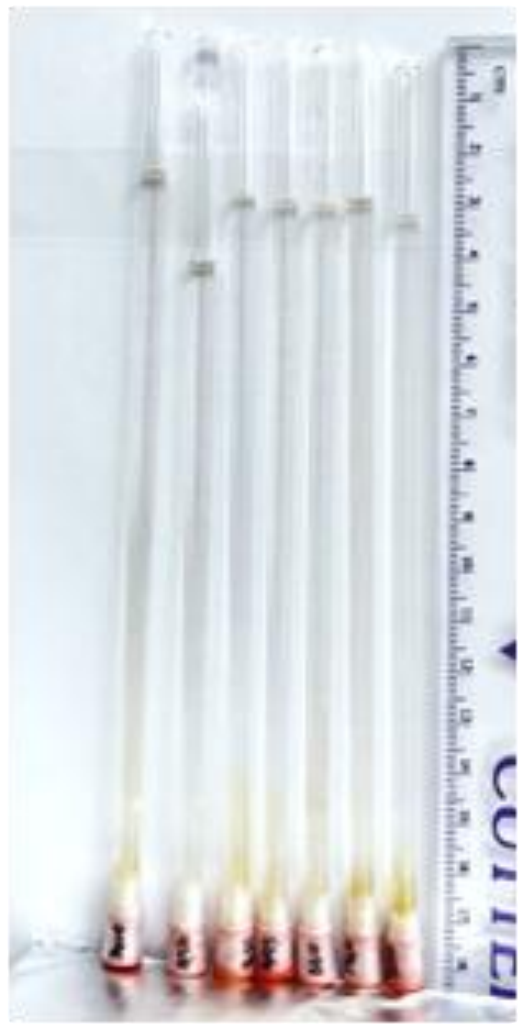

b

λ_{ex} (nm)	Length (mm)
dark	0
456	9
525	0
595	0
660[a]	0
740	0
850	0

Figure S22. (a) Photograph of the polymer gels formed after 2 h irradiation at different wavelengths. From left to right: dark control and excitation at 456, 525, 595, 660, 740, and 850 nm. Reaction condition: $[PETA]_0$:$[DPI]_0$ = 200:1 in 50% (v/v) DMSO, Ar atmosphere. (b) Polymer gel lengths obtained after 2 h irradiation at different wavelengths. [a] The 660 nm LED was equipped with a 645 nm long-pass optical filter.

10 g-scale experiment.

For the scale-up experiment, 10 mL of the reaction mixture was transferred into a 20 mL vial and irradiated with an 850 nm LED for 2 h. After irradiation, a freestanding gel was obtained.

9. NMR Spectra and mass spectra

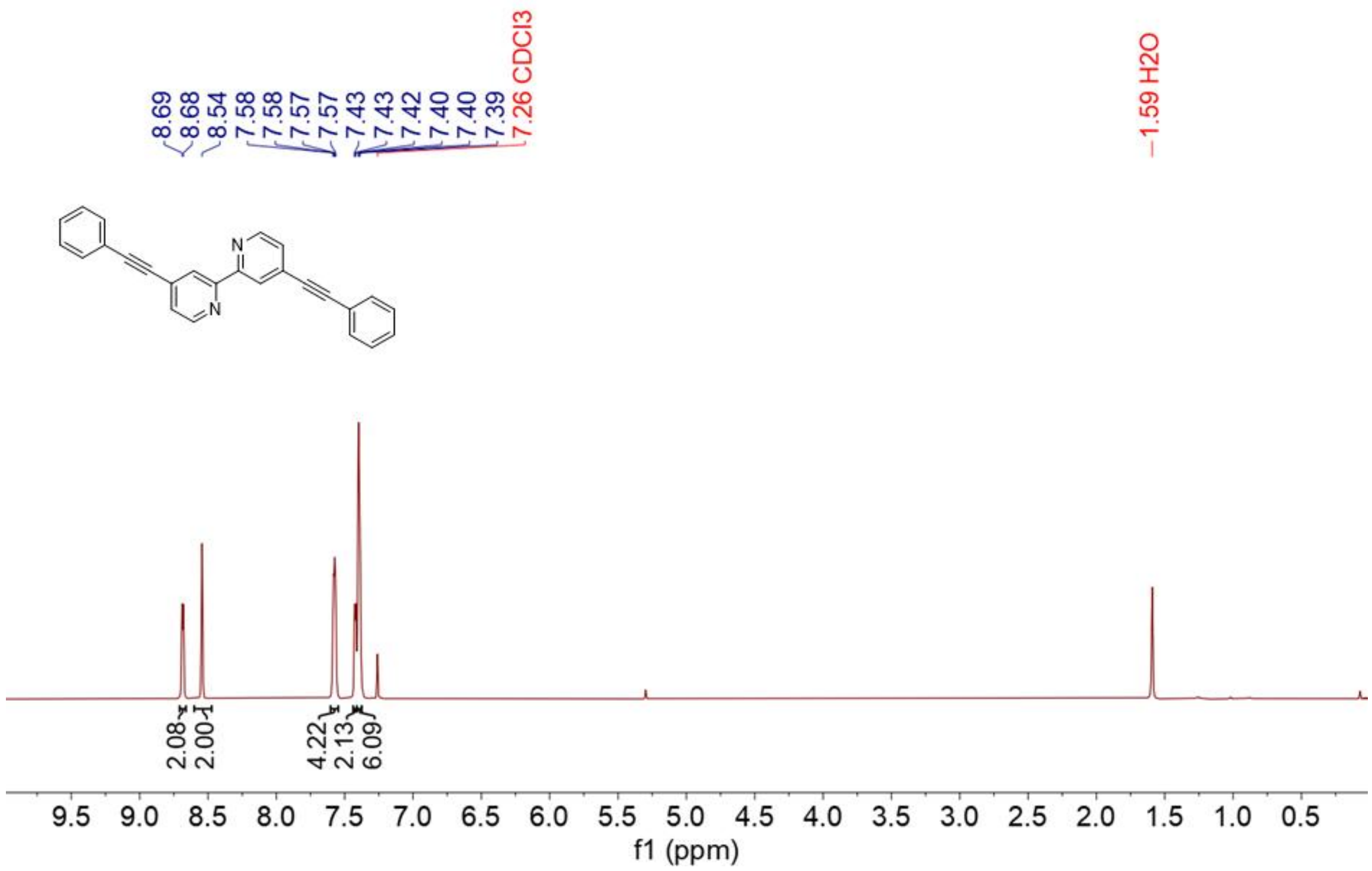

Figure S23. ^{1}H NMR spectrum of bpyap (500 MHz, $CDCl_3$).

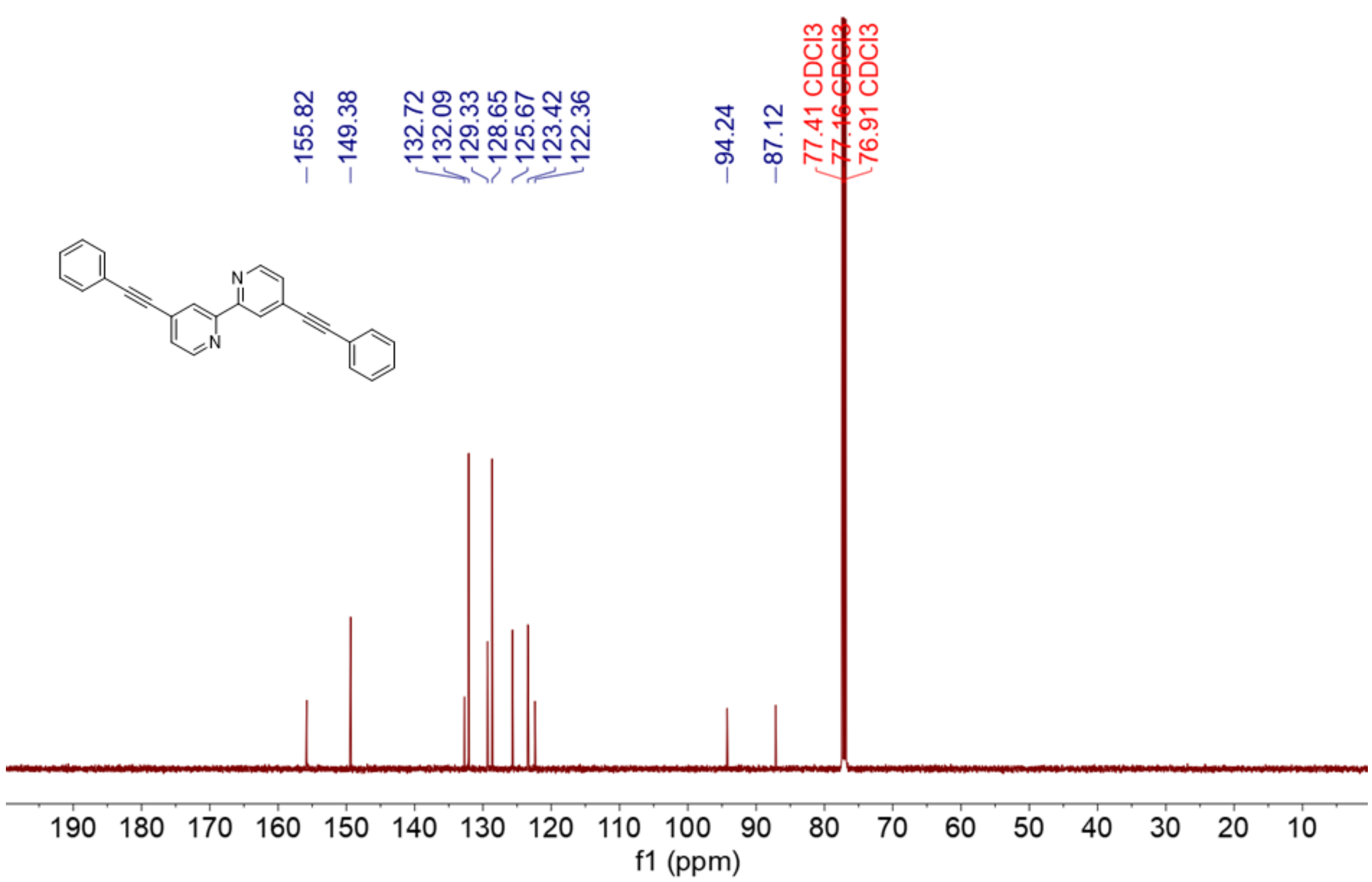

Figure S24. $^{13}C\{^{1}H\}$ NMR spectrum of bpyap (126 MHz, $CDCl_3$).

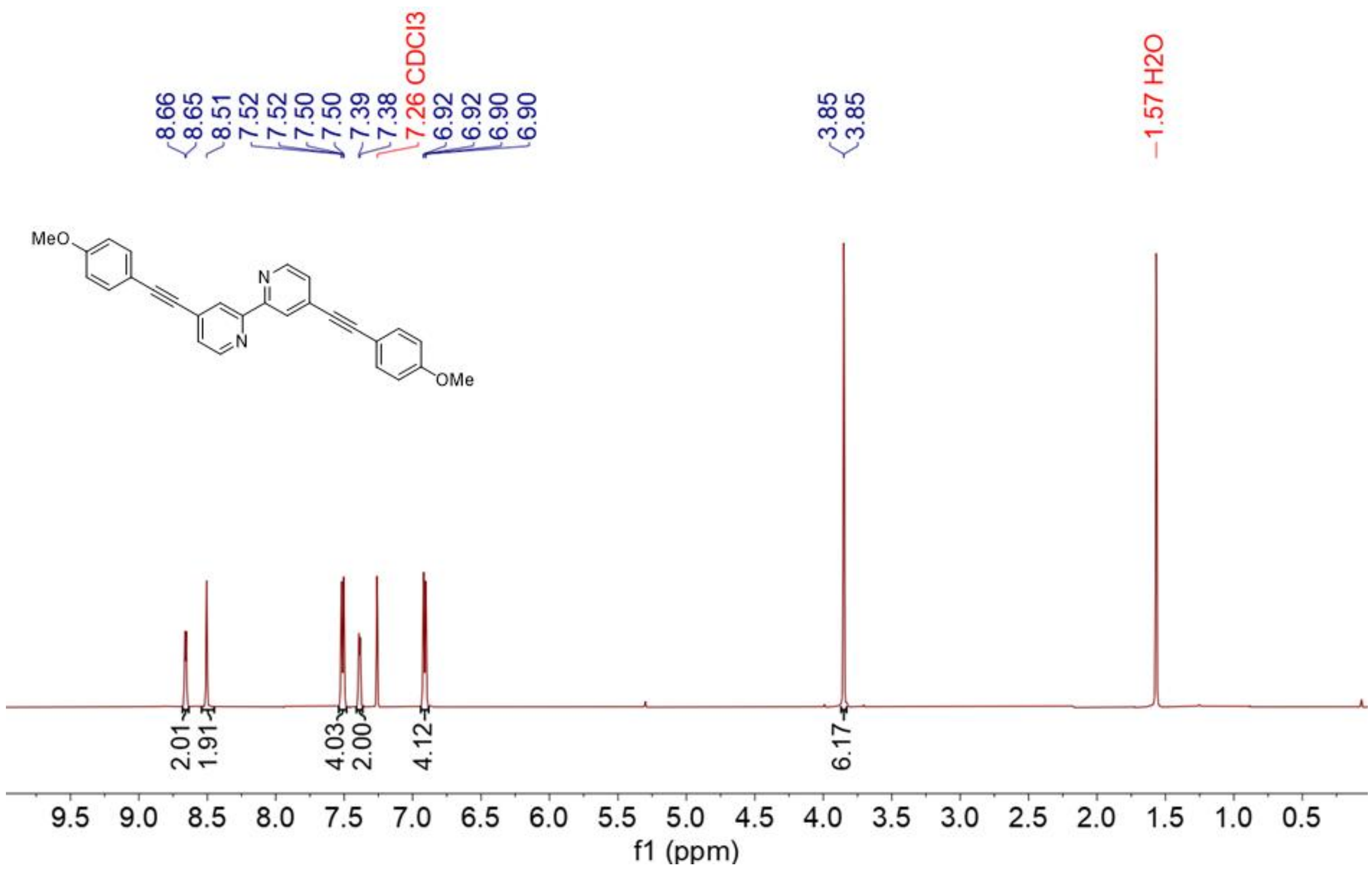

Figure S25. ^{1}H NMR spectrum of bpyap-OMe (500 MHz, $CDCl_3$).

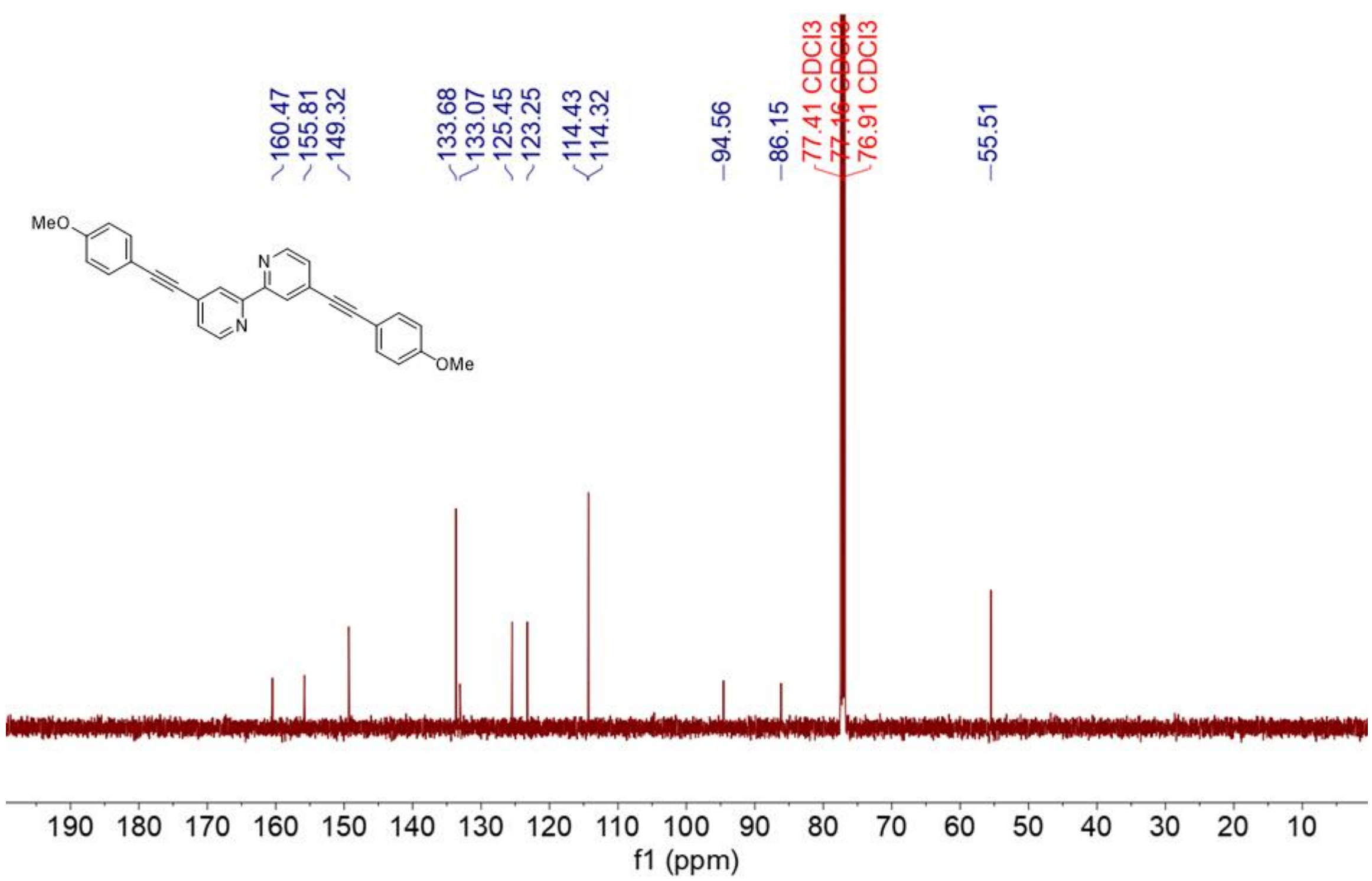

Figure S26. $^{13}C\{^{1}H\}$ NMR spectrum of bpyap-OMe (126 MHz, $CDCl_3$).

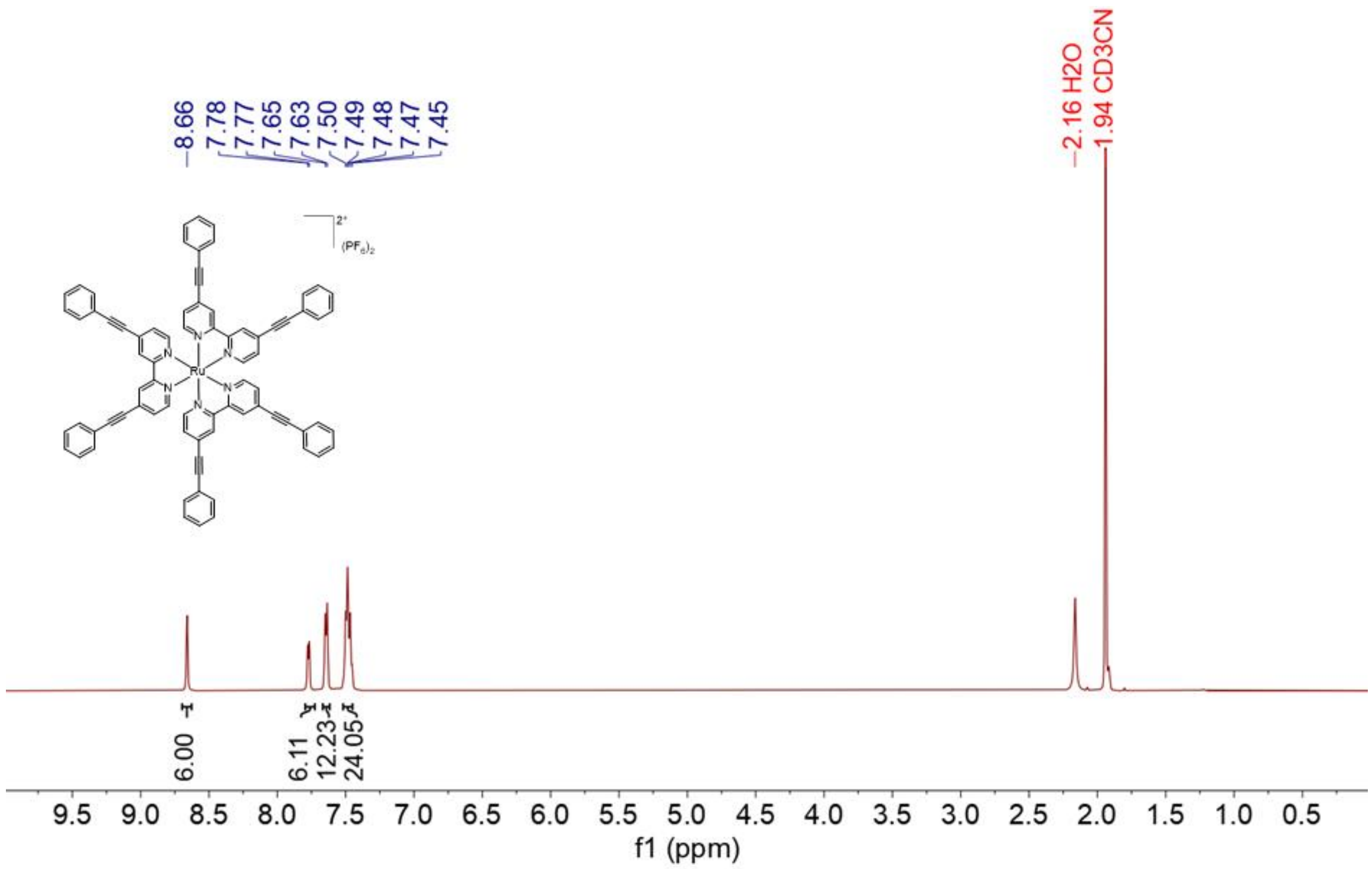

Figure S27. ^{1}H NMR spectrum of Ru-bpyap (500 MHz, CD_3CN).

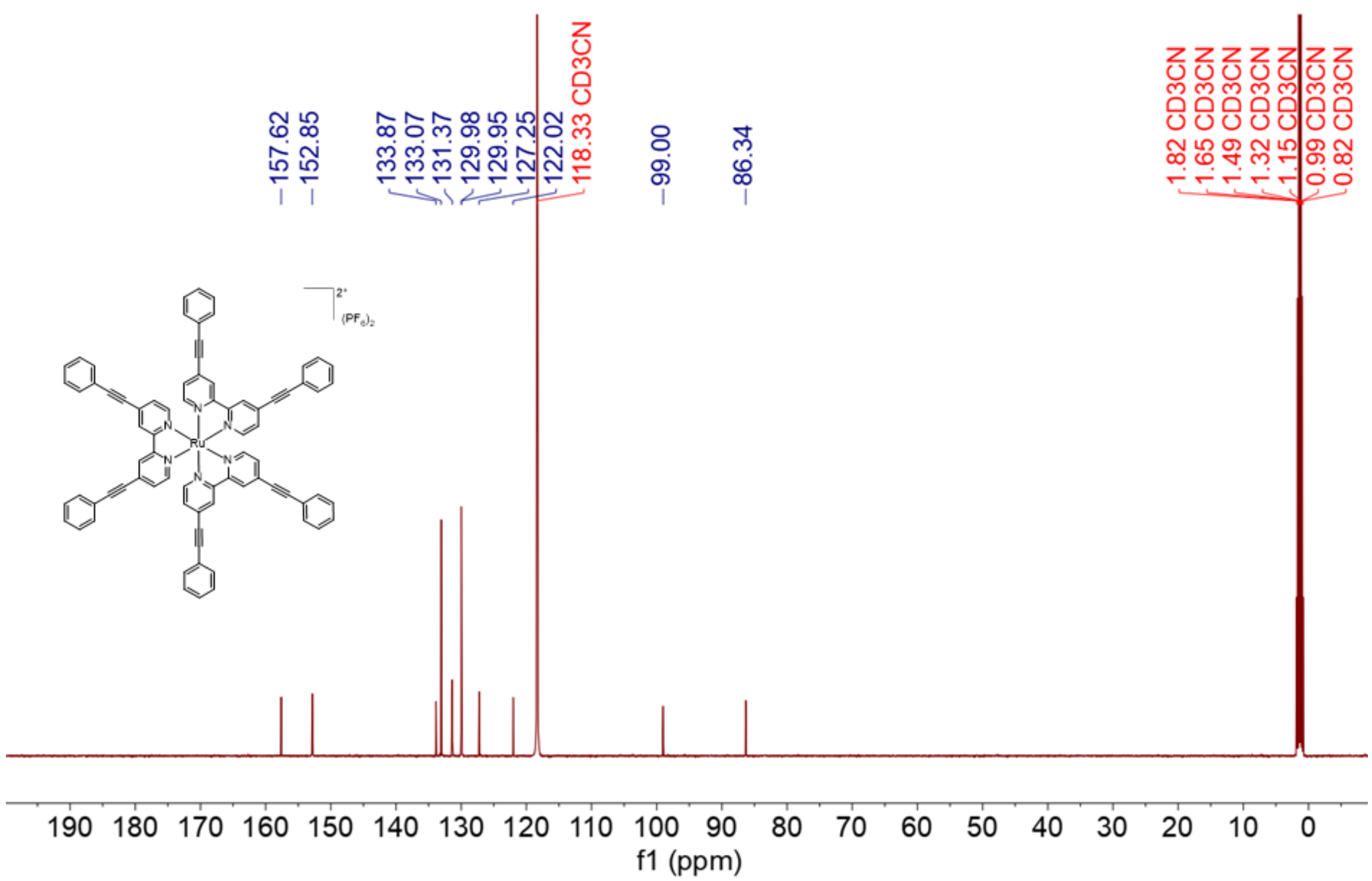

Figure S28. $^{13}C\{^{1}H\}$ NMR spectrum of Ru-bpyap (126 MHz, CD_3CN).

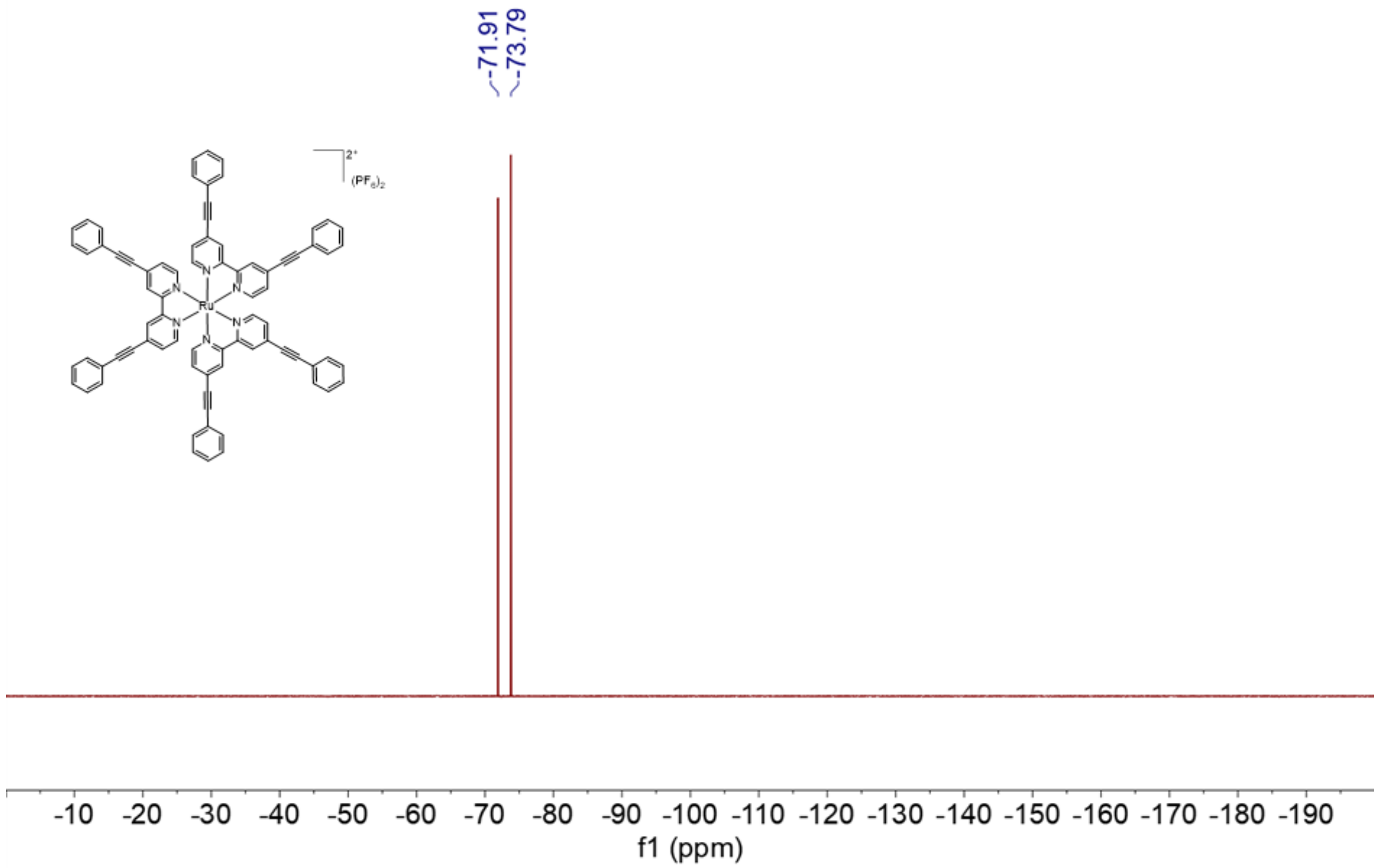

Figure S29. ^{19}F NMR spectrum of Ru-bpyap (376 MHz, CD_3CN).

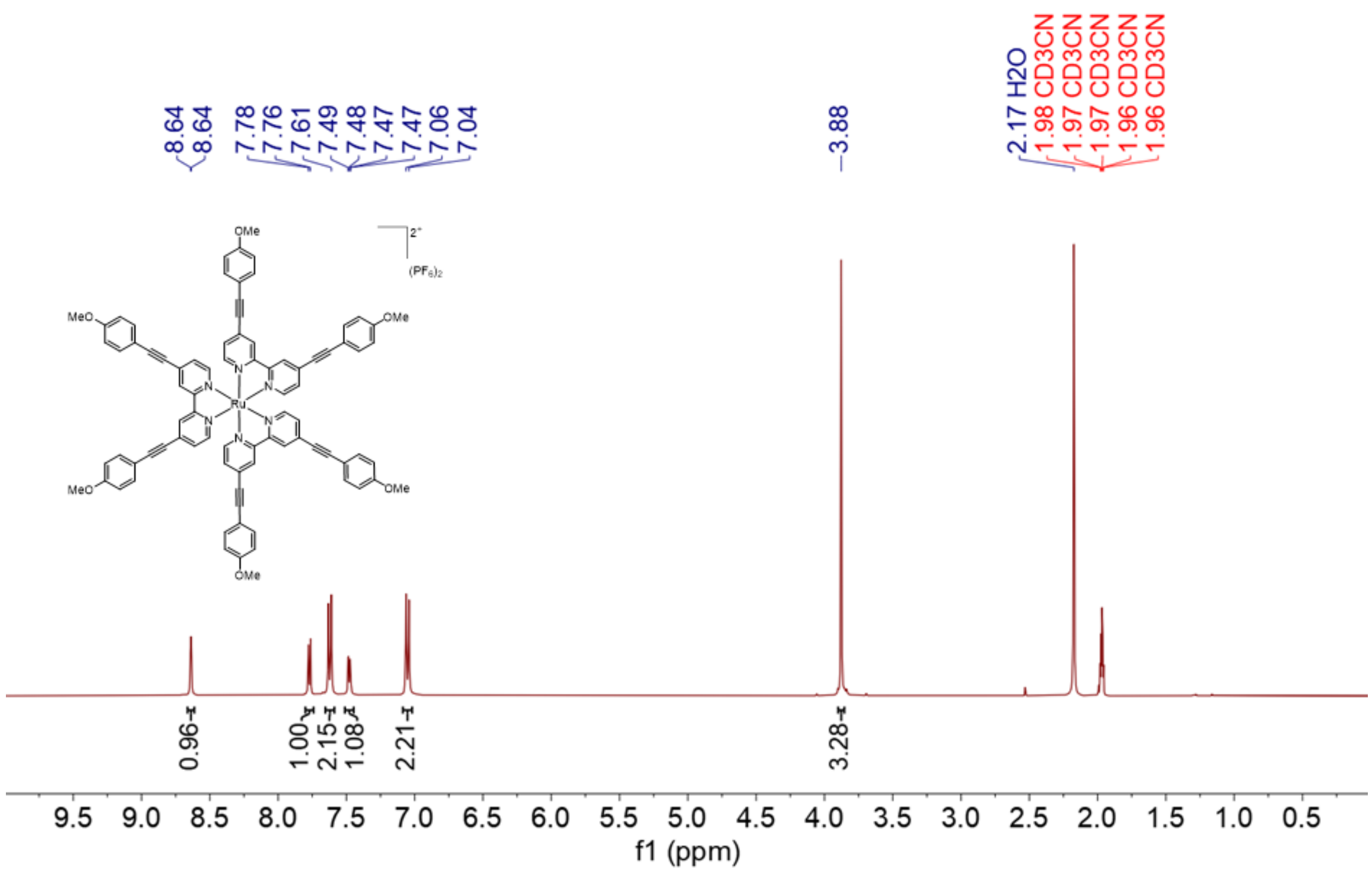

Figure S30. ^{1}H NMR spectrum of Ru-bpyap-OMe (500 MHz, CD_3CN).

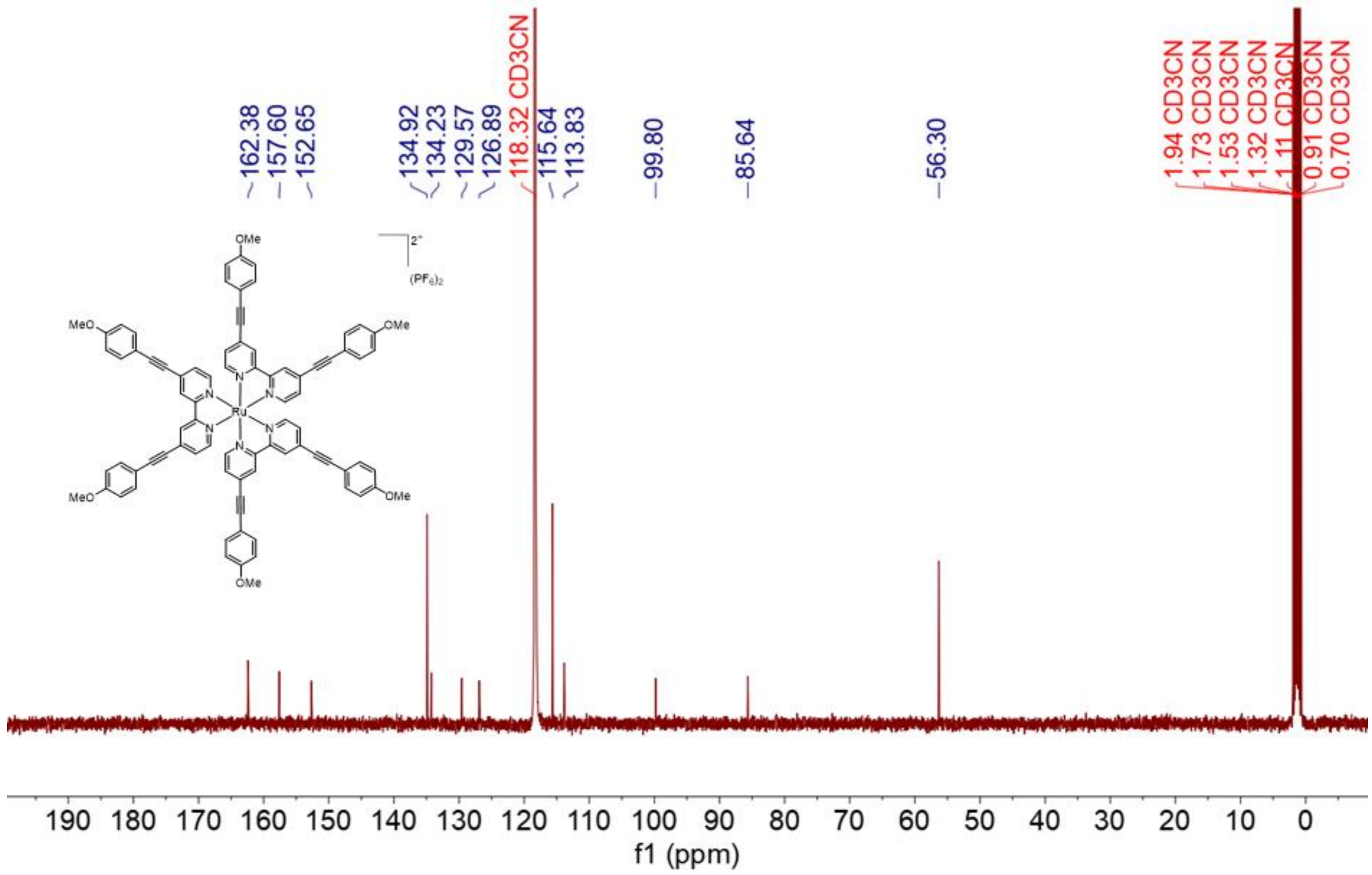

Figure S31. $^{13}C\{^{1}H\}$ NMR spectrum of Ru-bpyap-OMe (126 MHz, CD_3CN).

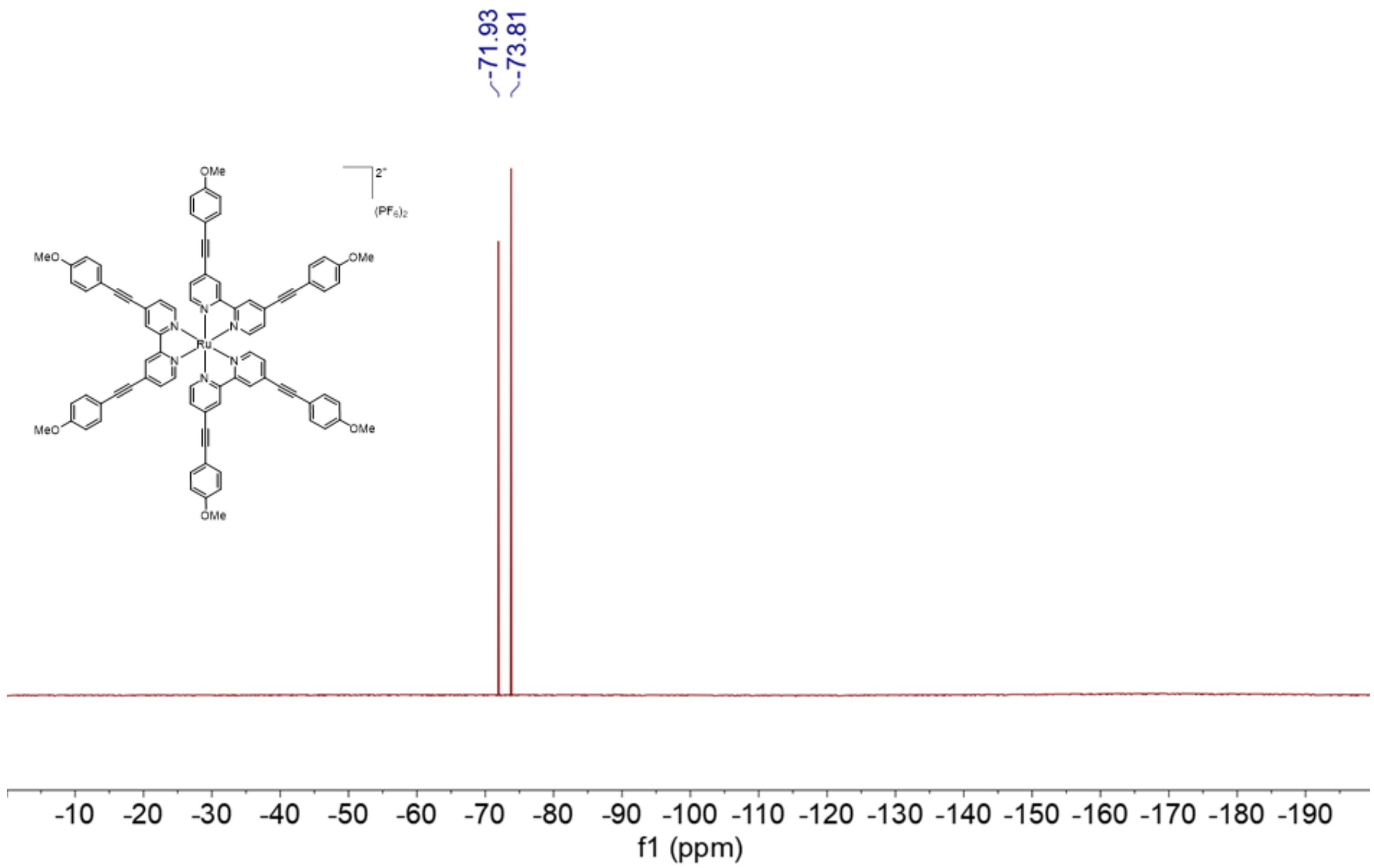

Figure S32. ^{19}F NMR spectrum of Ru-bpyap-OMe (376 MHz, CD_3CN).

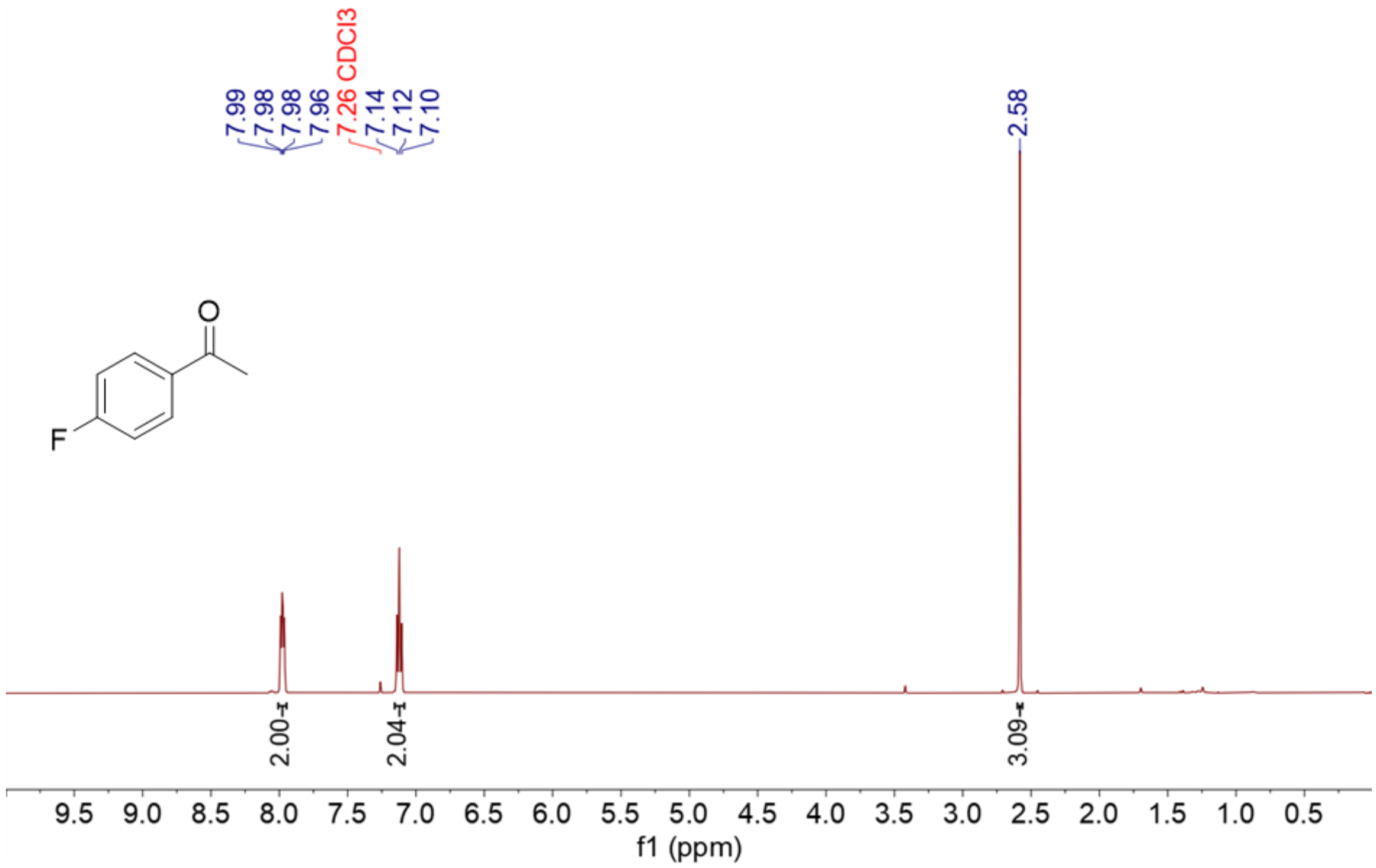

Figure S33. ^{1}H NMR spectrum of 4′-fluoroacetophenone (500 MHz, $CDCl_3$).

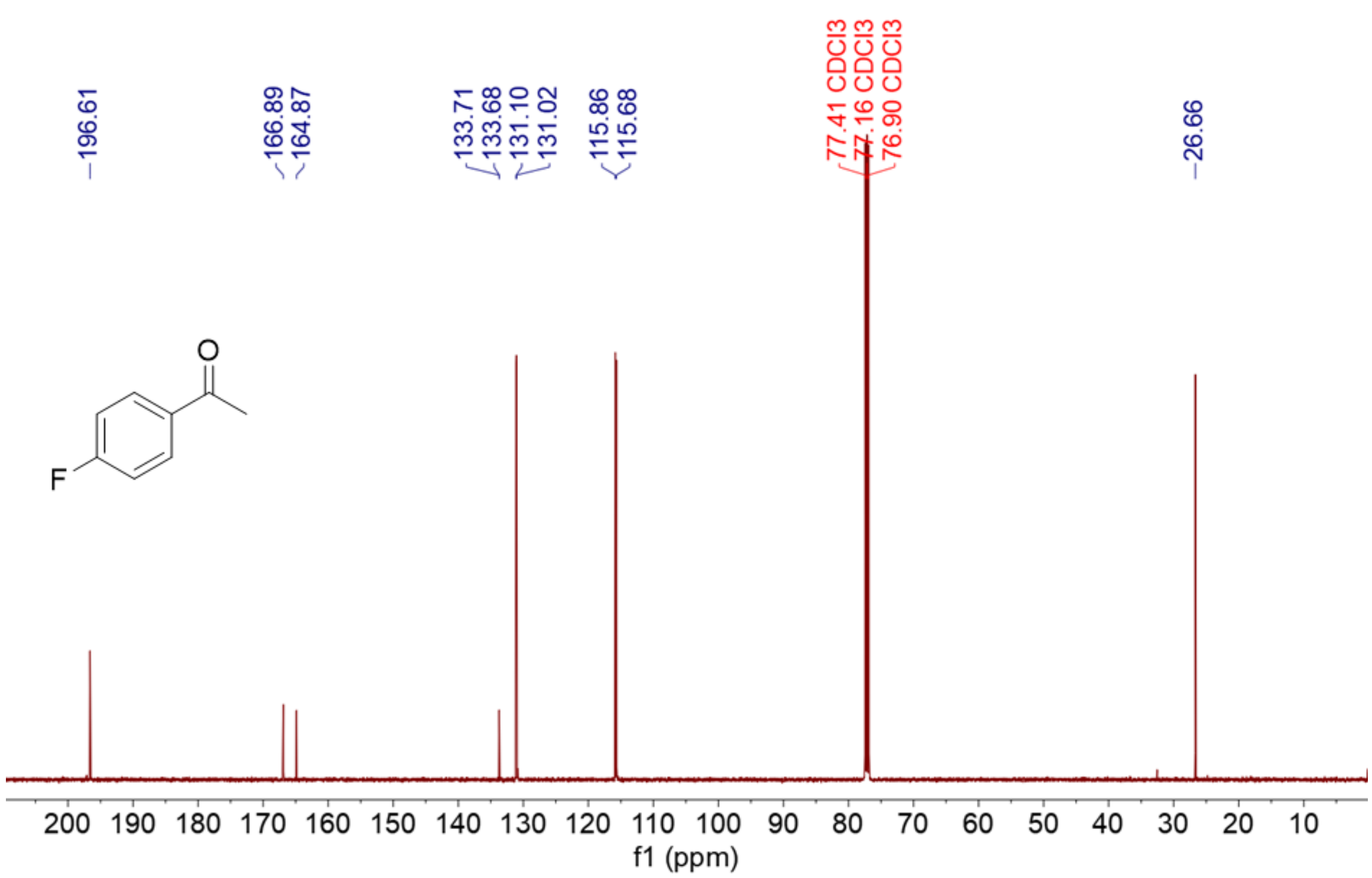

Figure S34. $^{13}C\{^1H\}$ NMR spectrum of 4′-fluoroacetophenone (126 MHz, $CDCl_3$).

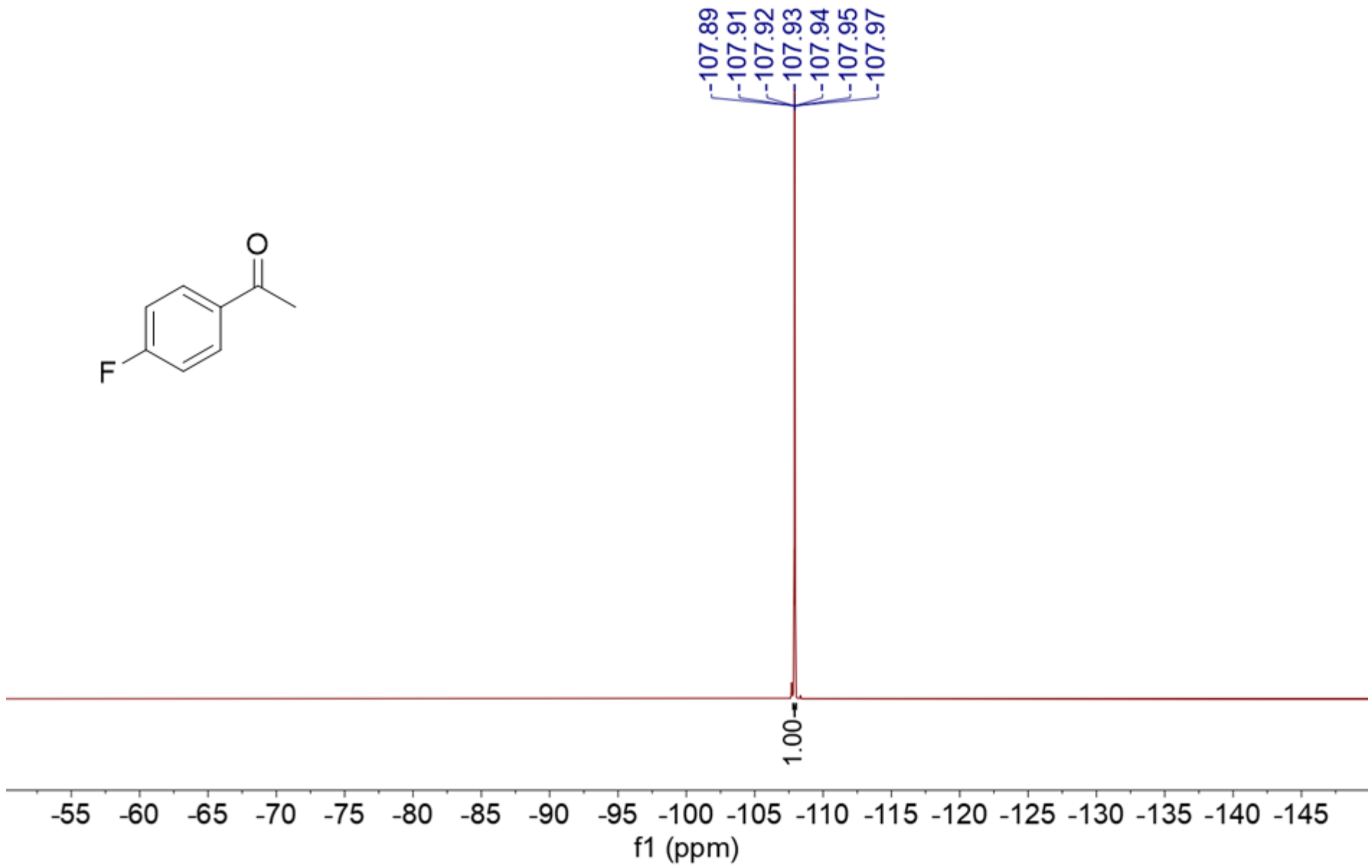

Figure S35. ^{19}F NMR spectrum of 4′-fluoroacetophenone (376 MHz, $CDCl_3$).

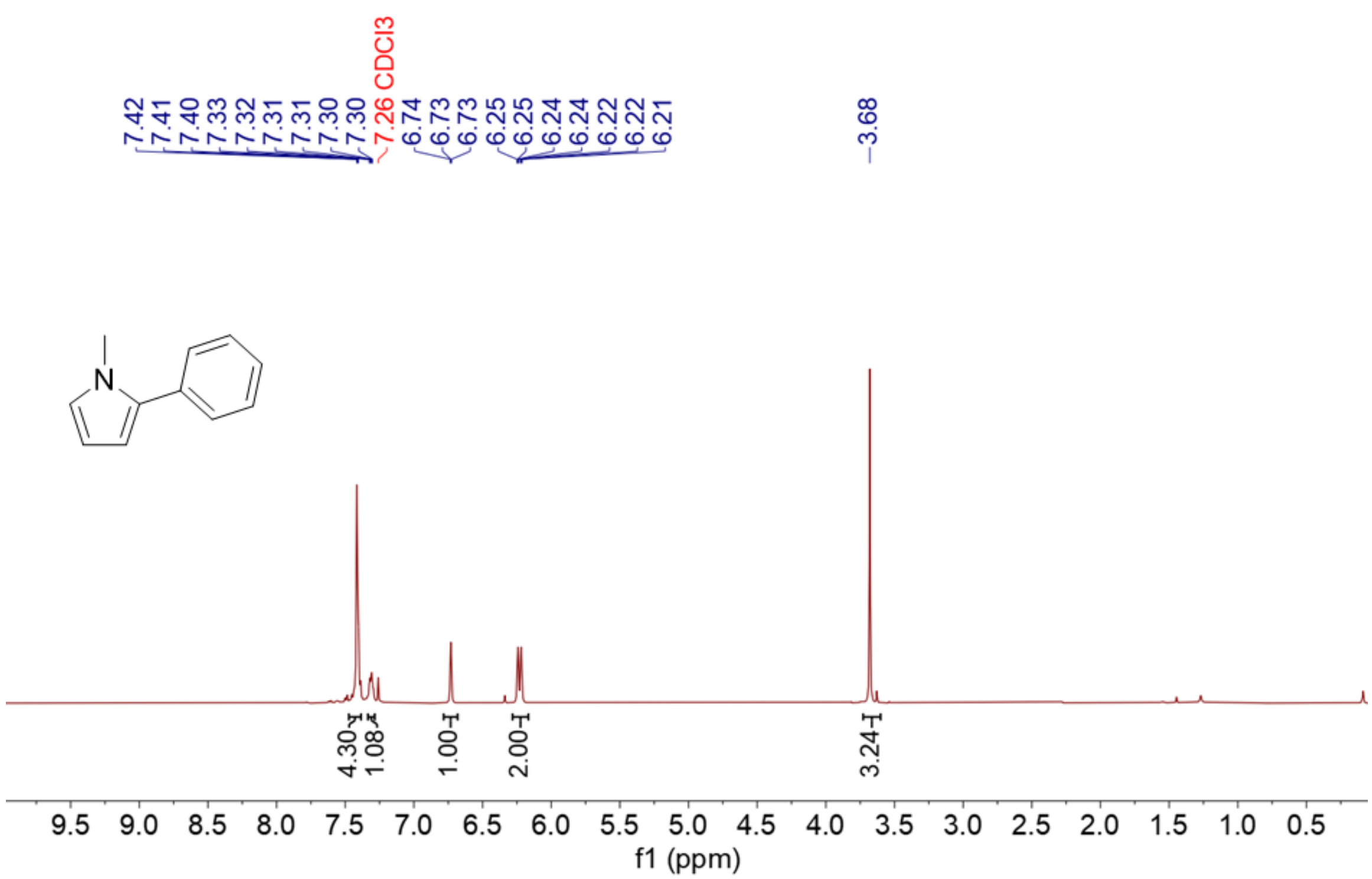

Figure S36. ^{1}H NMR spectrum of 1-methyl-2-phenyl-1H-pyrrole (500 MHz, $CDCl_3$).

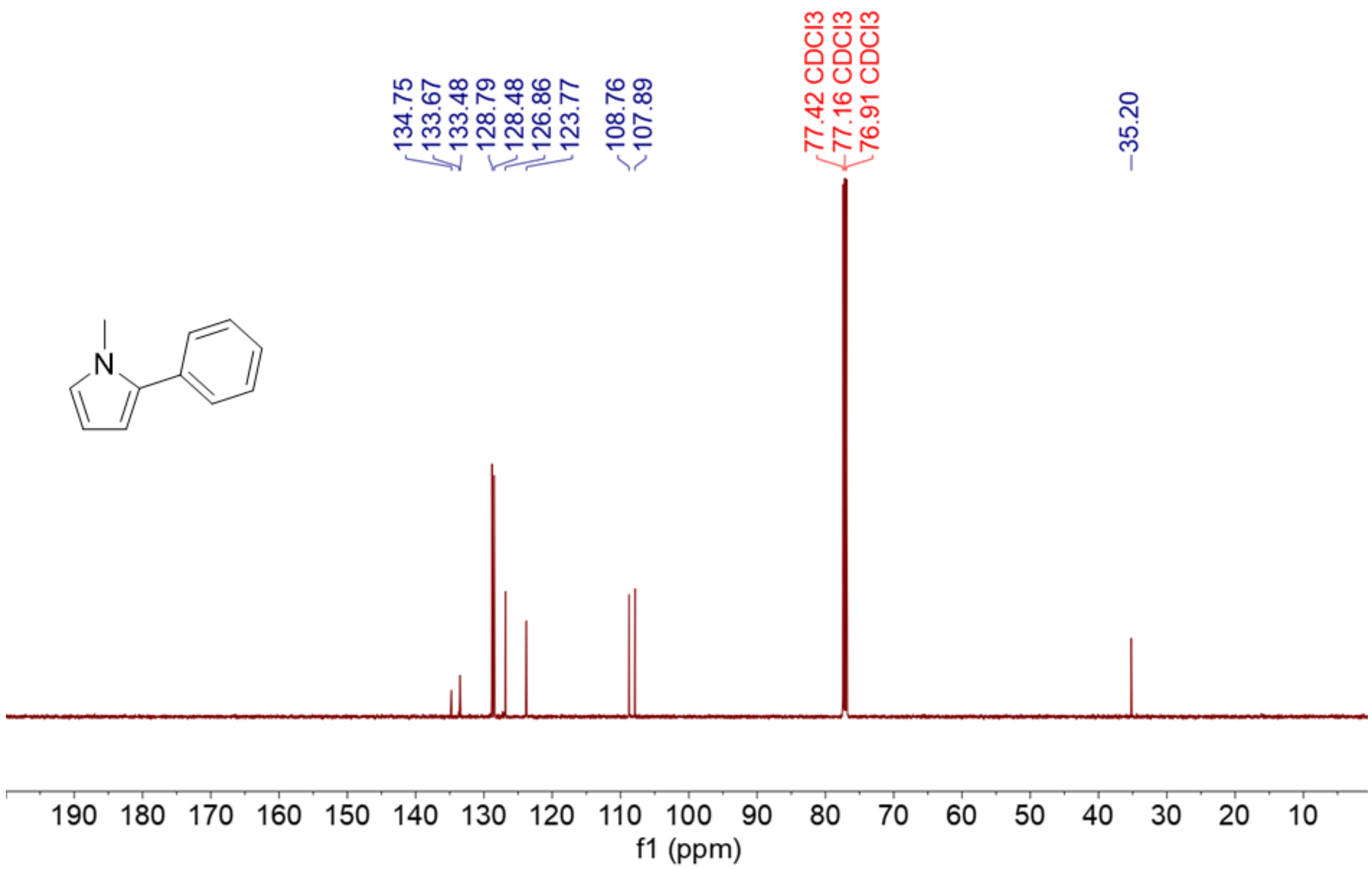

Figure S37. $^{13}C\{^{1}H\}$ NMR spectrum of 1-methyl-2-phenyl-1H-pyrrole (126 MHz, $CDCl_3$).

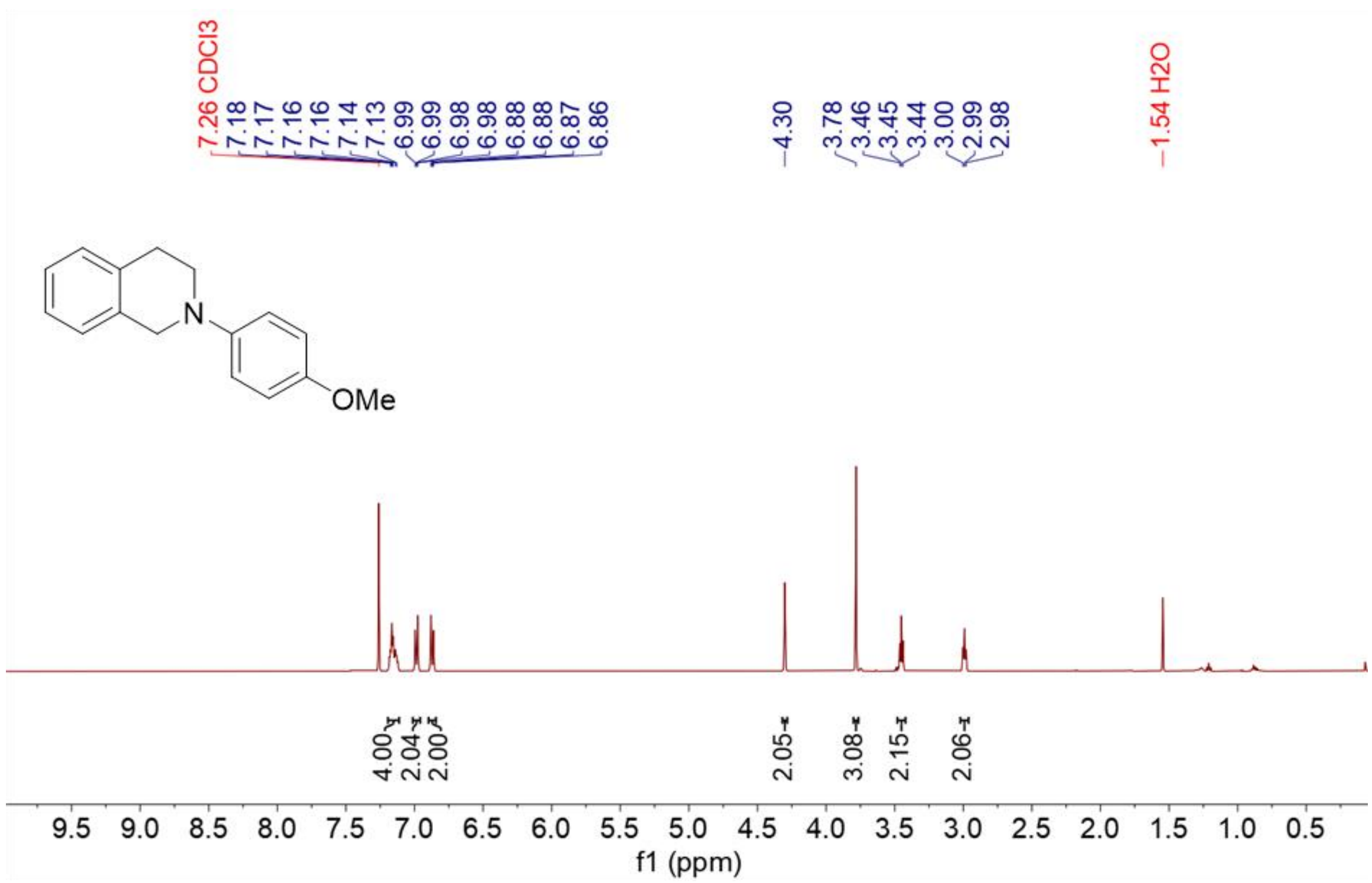

Figure S38. ^{1}H NMR spectrum of 2-(4-methoxyphenyl)-1,2,3,4-tetrahydroisoquinoline (500 MHz, $CDCl_3$).

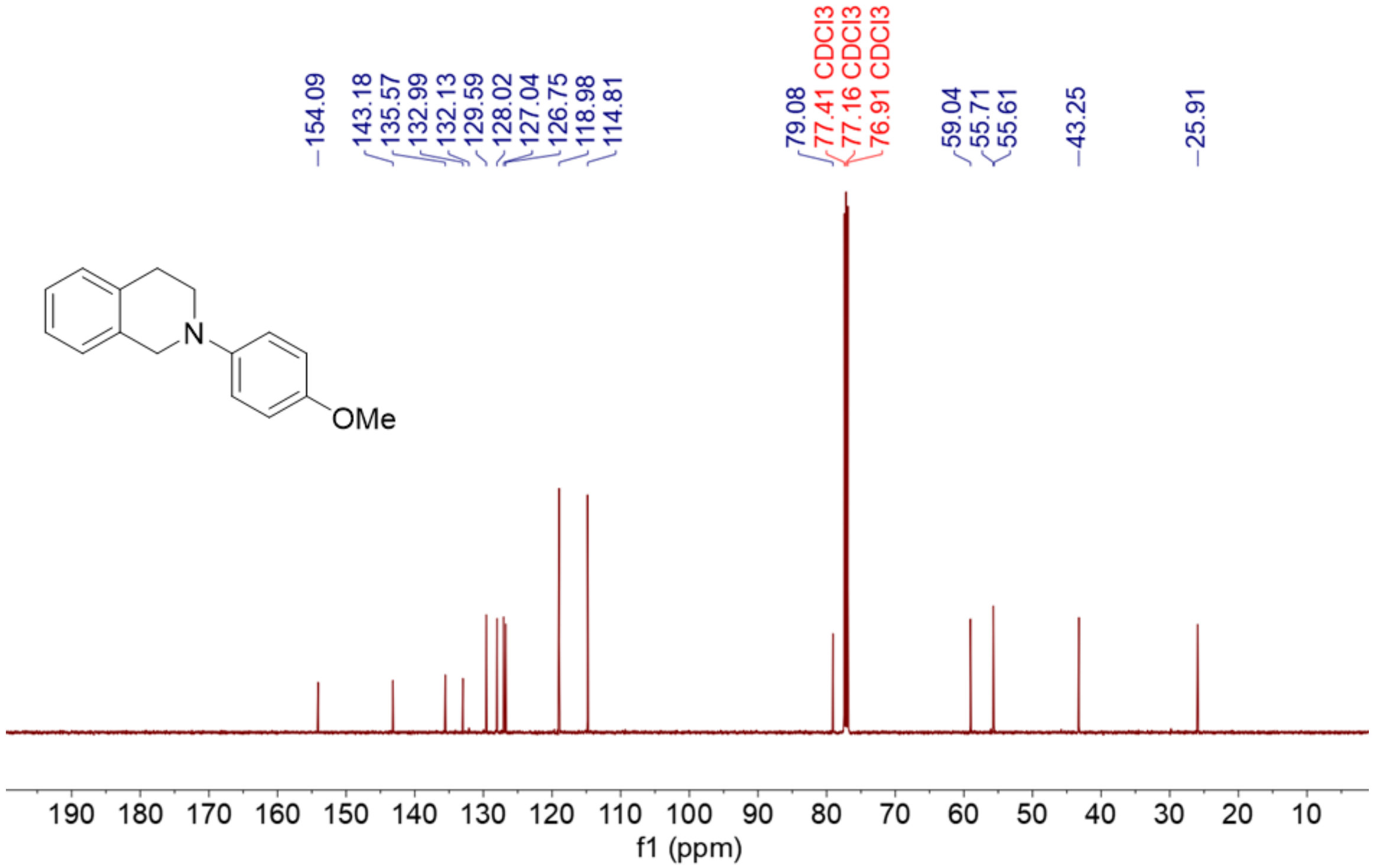

Figure S39. $^{13}C\{^{1}H\}$ NMR spectrum of 2-(4-methoxyphenyl)-1,2,3,4-tetrahydroisoquinoline (126 MHz, $CDCl_3$).

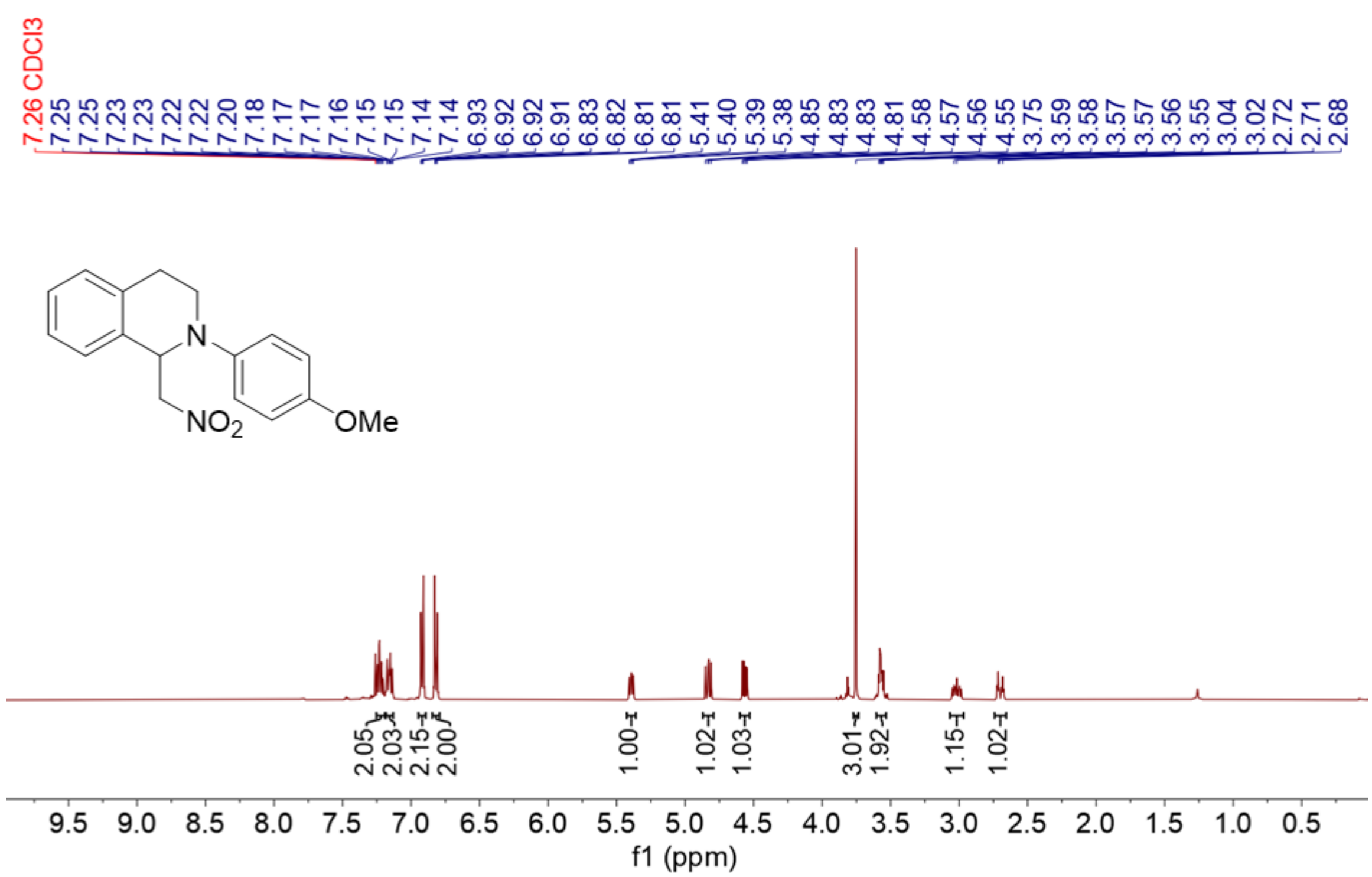

Figure S40. ^{1}H NMR spectrum of 1-(nitromethyl)-2-(4-methoxyphenyl)-1,2,3,4-tetrahydroisoquinoline (500 MHz, $CDCl_3$).

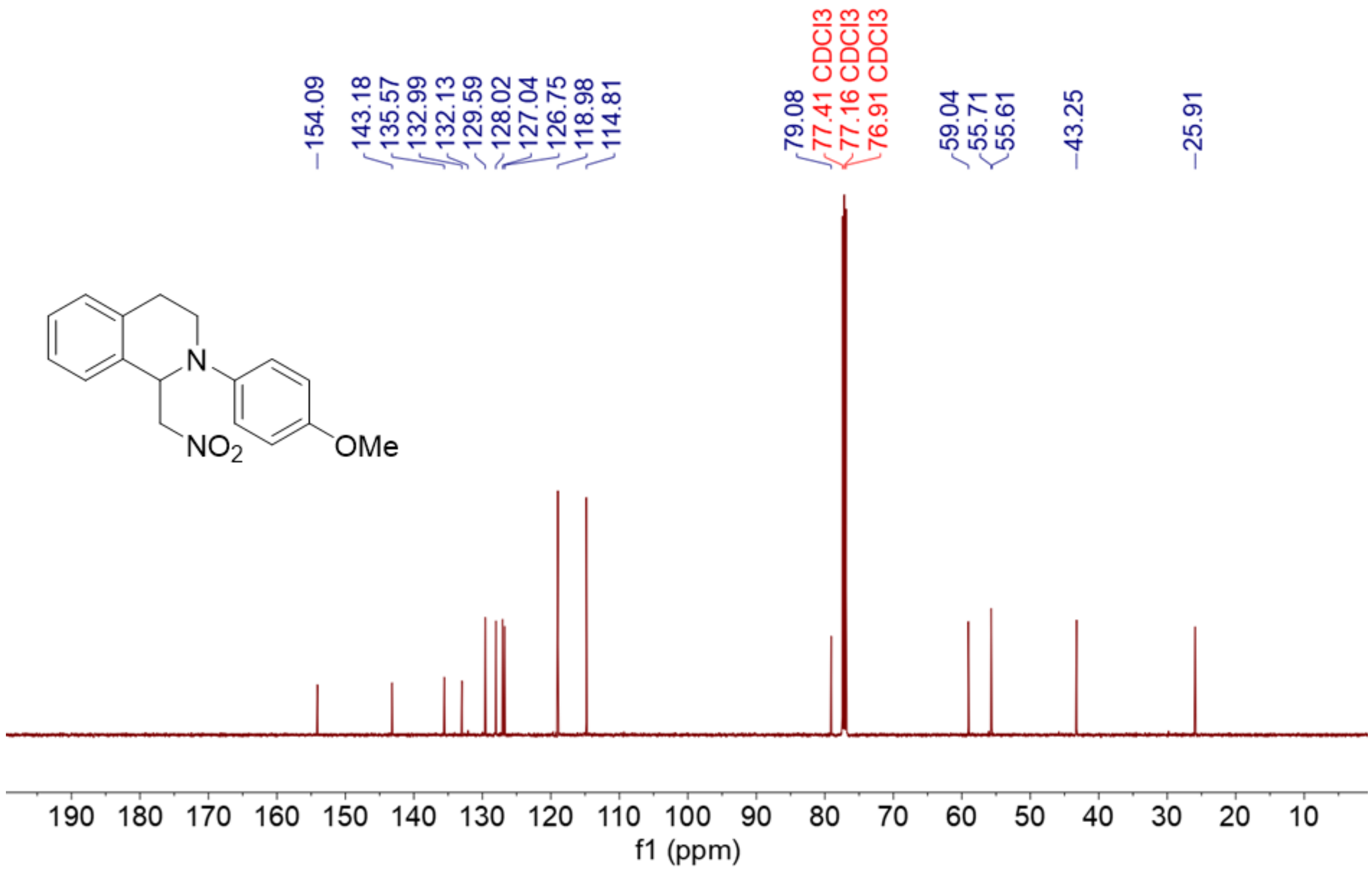

Figure S41. $^{13}C\{^{1}H\}$ NMR spectrum of 1-(nitromethyl)-2-(4-methoxyphenyl)-1,2,3,4-tetrahydroisoquinoline (126 MHz, $CDCl_3$).

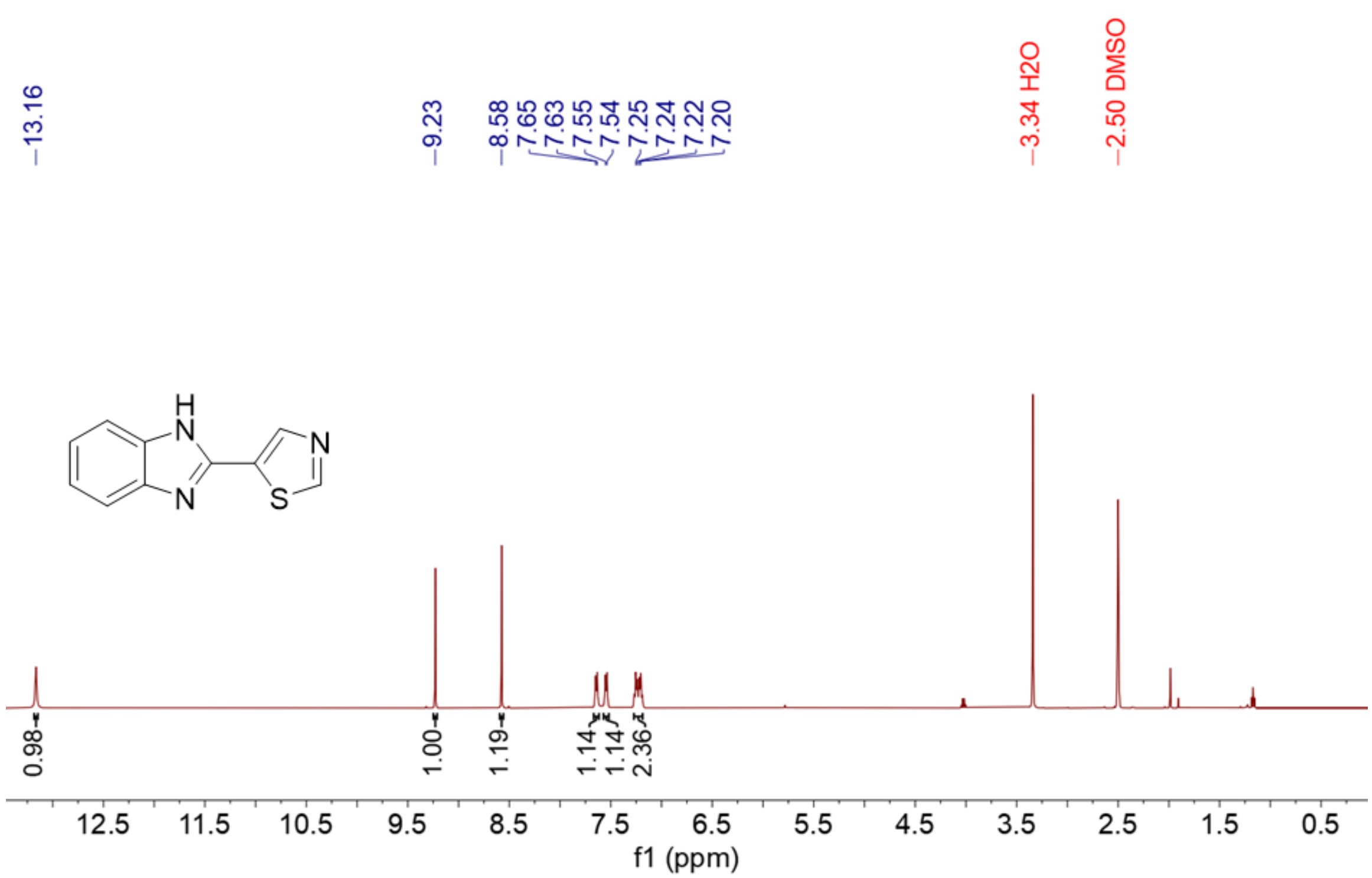

Figure S42. ^{1}H NMR spectrum of 2-(5-thiazolyl)-1H-benzimidazole (500 MHz, DMSO).

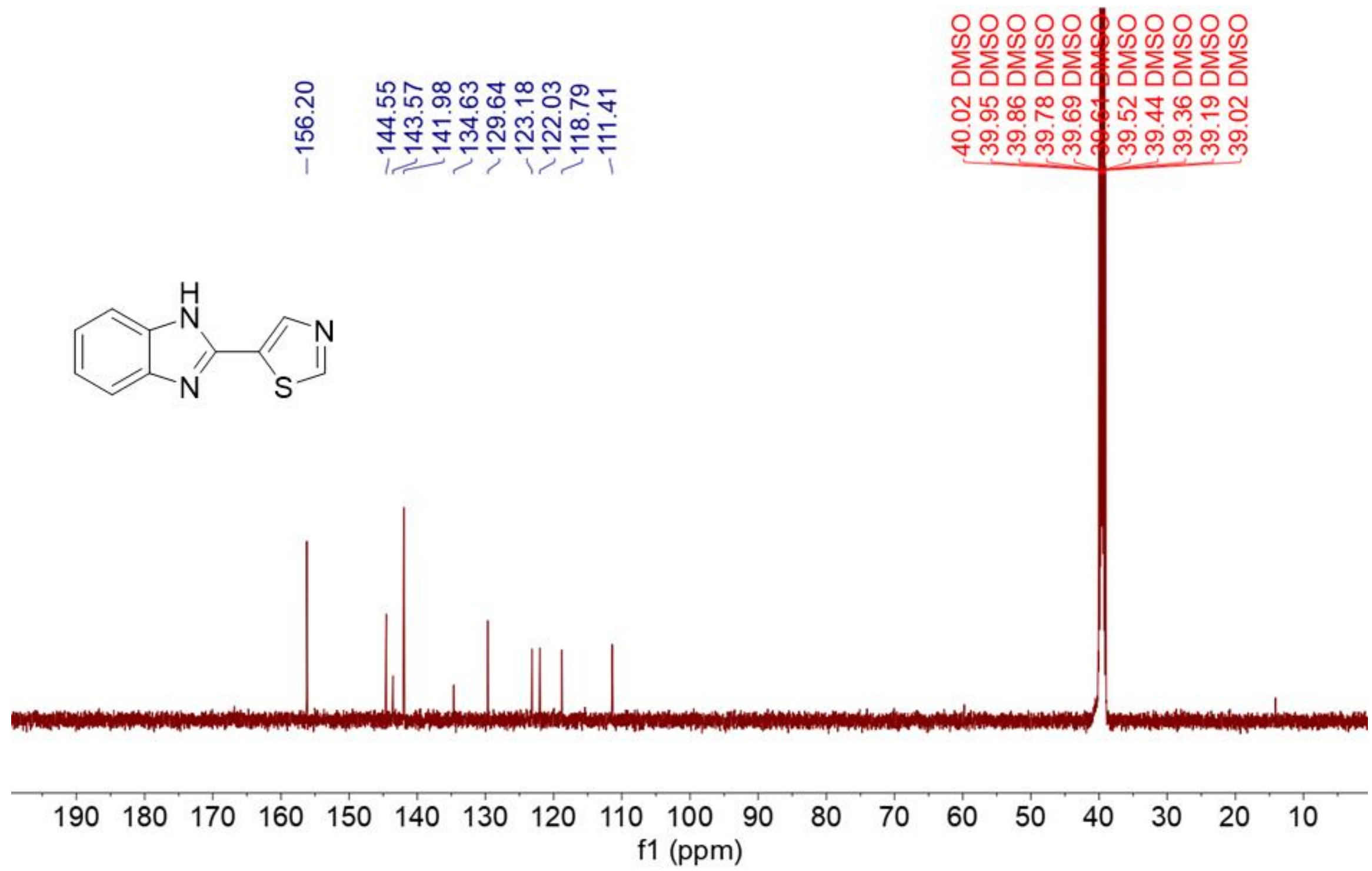

Figure S43. $^{13}C\{^{1}H\}$ NMR spectrum of 2-(5-thiazolyl)-1H-benzimidazole (126 MHz, DMSO).

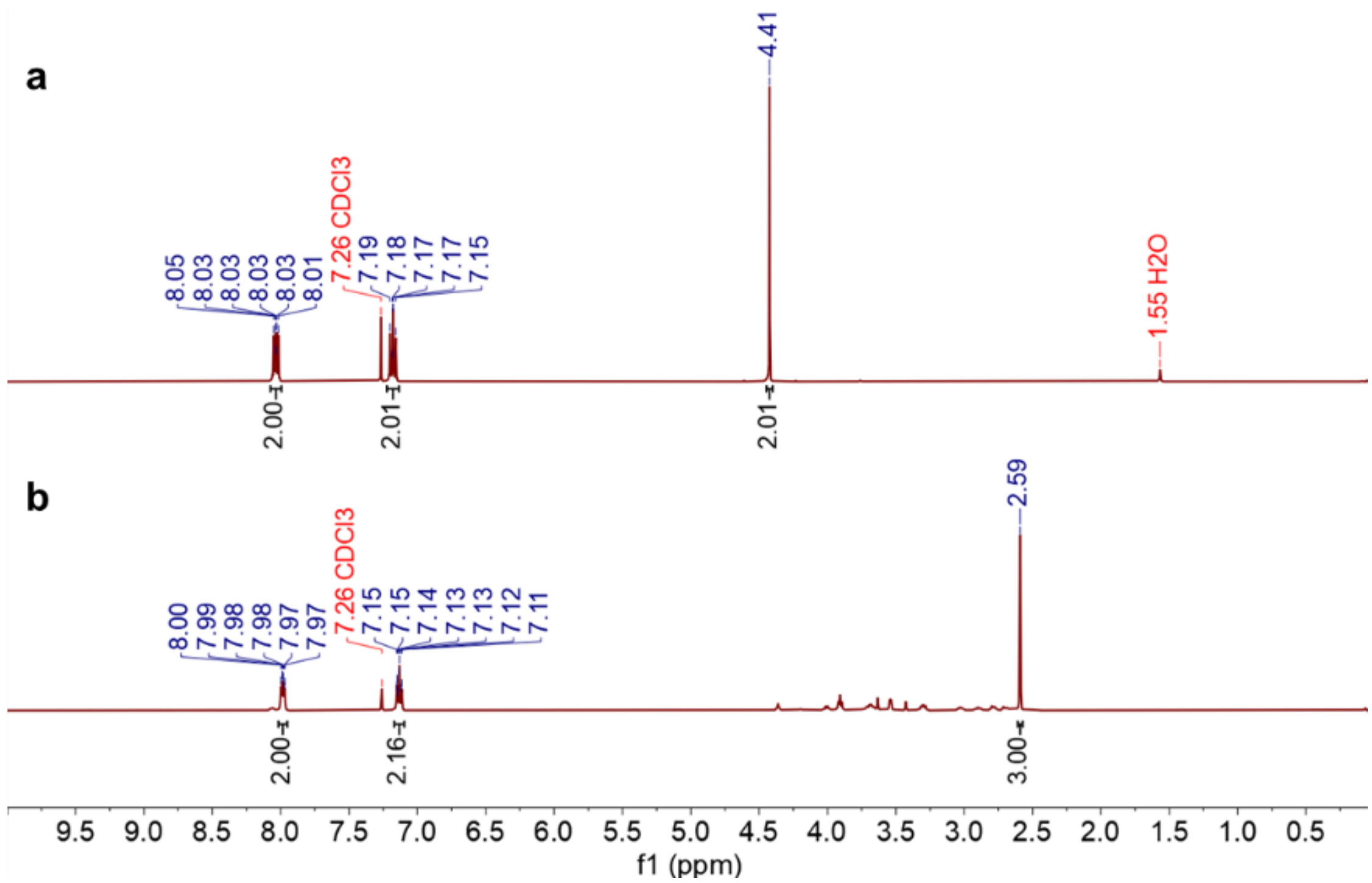

Figure S44. ^{1}H NMR spectra of the reaction mixture in $CDCl_3$ before (**a**) and after (**b**) irradiation from the photocatalytic hydrodebromination reaction. Conditions: 50 mM 2-bromo-4′-fluoroacetophenone, Ru-bpyap-OMe (2 mol%), CH_3CN (2 mL), argon atmosphere, irradiation at 740 nm for 15 h at room temperature.

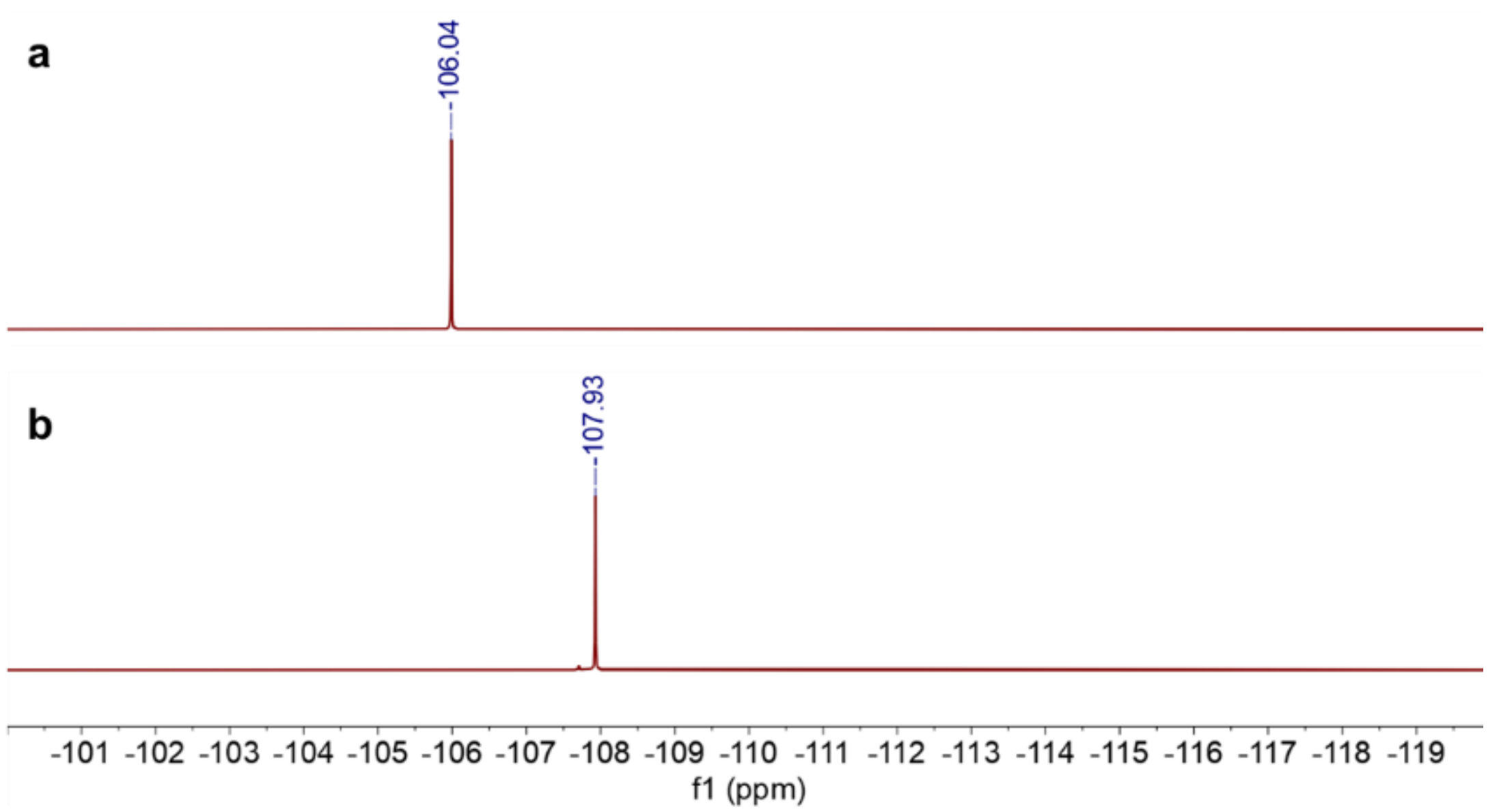

Figure S45. $^{19}F\{^{1}H\}$ NMR spectra of the reaction mixture in $CDCl_3$ before (**a**) and after (**b**) irradiation from the photocatalytic hydrodebromination reaction. Conditions: 50 mM 2-bromo-4′-fluoroacetophenone, Ru-bpyap-OMe (2 mol%), CH_3CN (2 mL), argon atmosphere, irradiation at 740 nm for 15 h at room temperature.

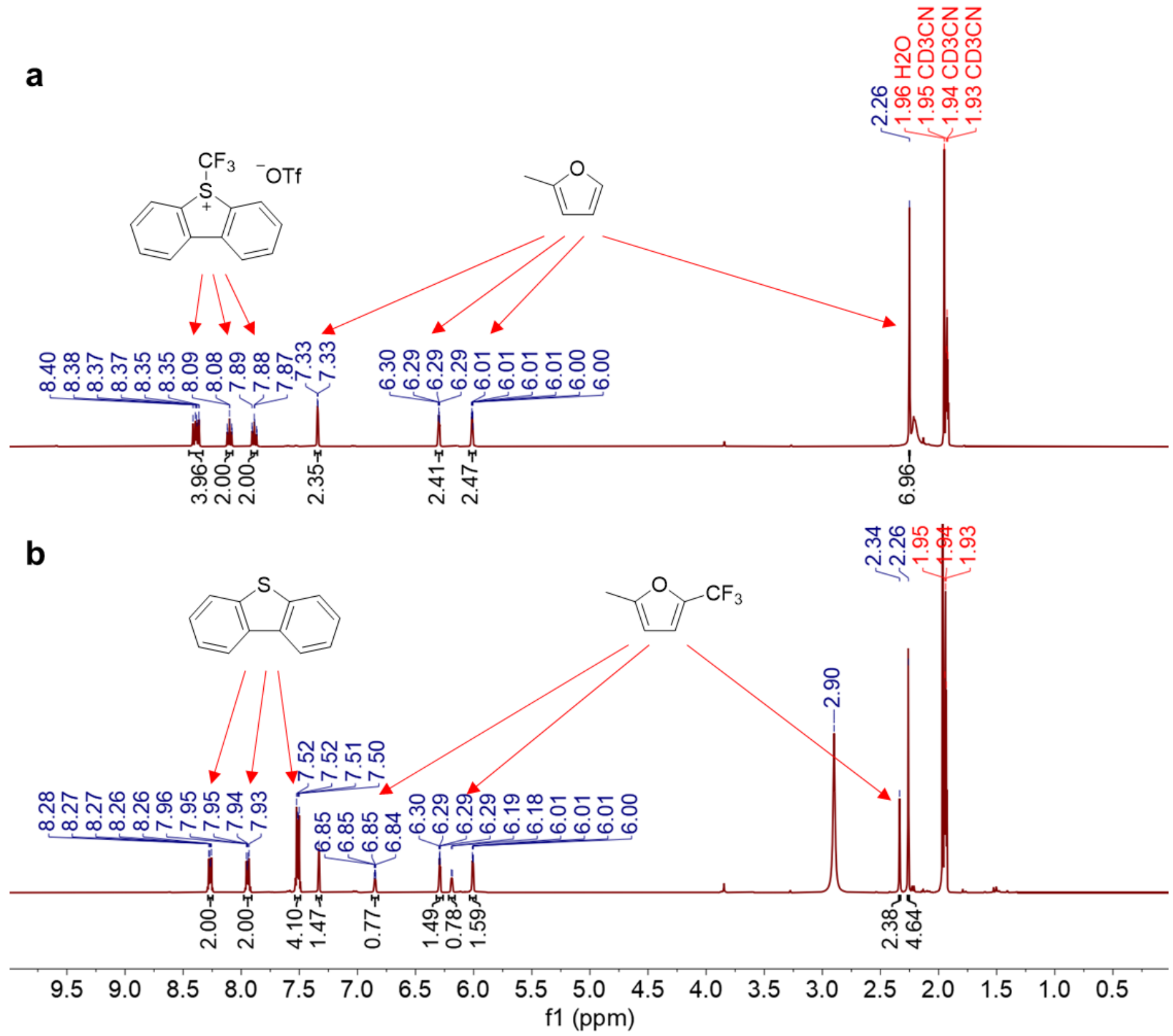

Figure S46. ^{1}H NMR spectra of the reaction mixture in CD_3CN before (**a**) and after (**b**) irradiation from the photocatalytic trifluoromethylation reaction. Conditions: 50 mM 5-(trifluoromethyl)dibenzothiophenium triflate, 2-methyl-furan (3 equiv.), dipotassium phosphate (2 equiv.), Ru-bpyap-OMe (2 mol%), CD_3CN (2 mL), argon atmosphere, irradiation at 850 nm for 24 h at room temperature.

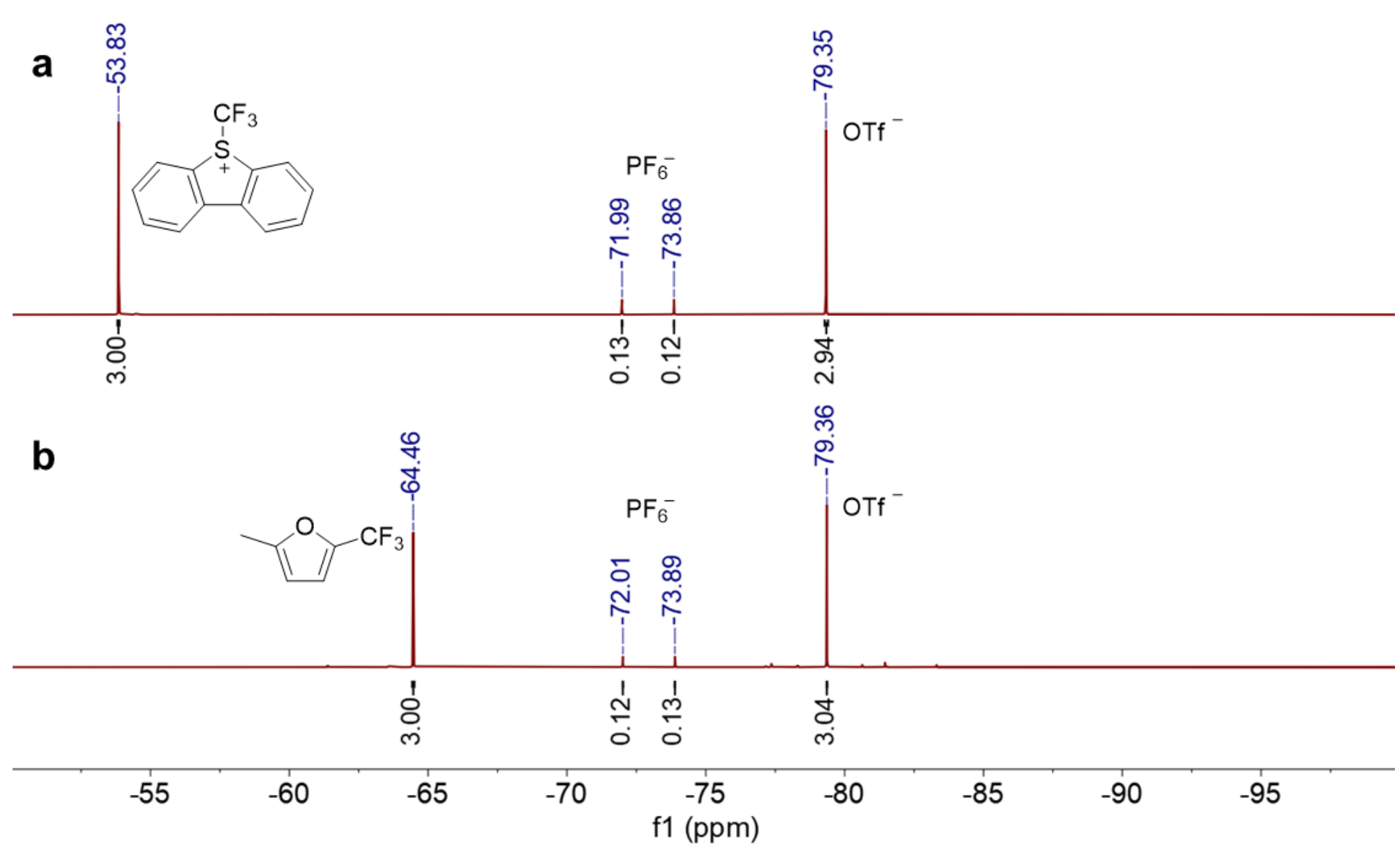

Figure S47. $^{19}F\{^{1}H\}$ NMR spectra of the reaction mixture in CD_3CN before (**a**) and after

(**b**) irradiation from the photocatalytic trifluoromethylation reaction. Conditions: 50 mM 5-(trifluoromethyl)dibenzothiophenium triflate, 2-methyl-furan (3 equiv.), dipotassium phosphate (2 equiv.), Ru-bpyap-OMe (2 mol%), CD_3CN (2 mL), argon atmosphere, irradiation at 850 nm for 24 h at room temperature.

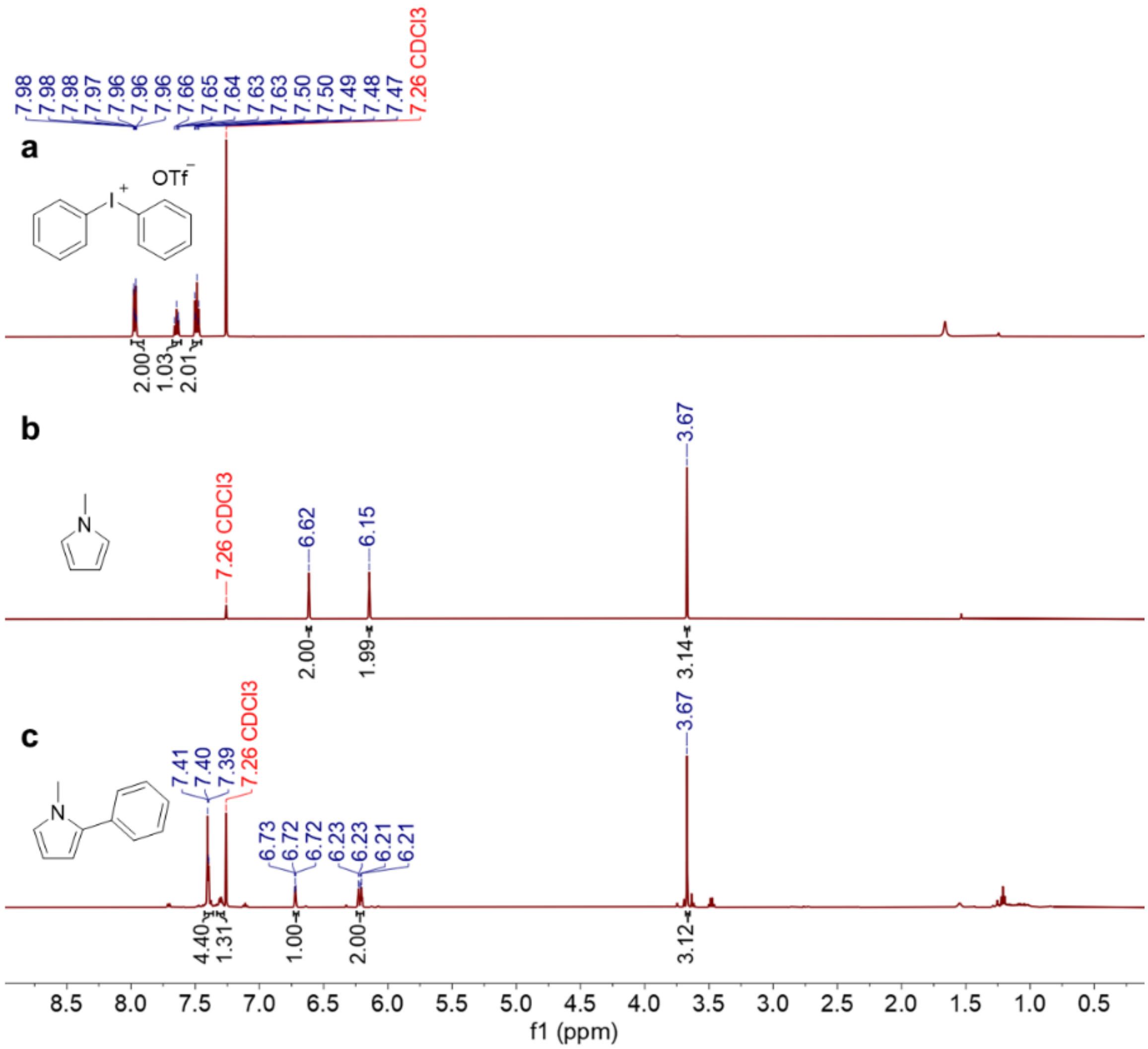

Figure S48. ^{1}H NMR spectra of (**a**) diphenyliodinium triflate, (**b**) 1-methyl-1H-pyrrole, and (**c**) the reaction mixture in $CDCl_3$ after irradiation from the photocatalytic phenylation reaction. Conditions: 50 mM diphenyliodinium triflate,1-methyl-1H-pyrrole (3 equiv.), dipotassium phosphate (2 equiv.), Ru-bpyap-OMe (5 mol%), CH_3CN (2 mL), argon atmosphere, irradiation at 850 nm for 40 h at room temperature.

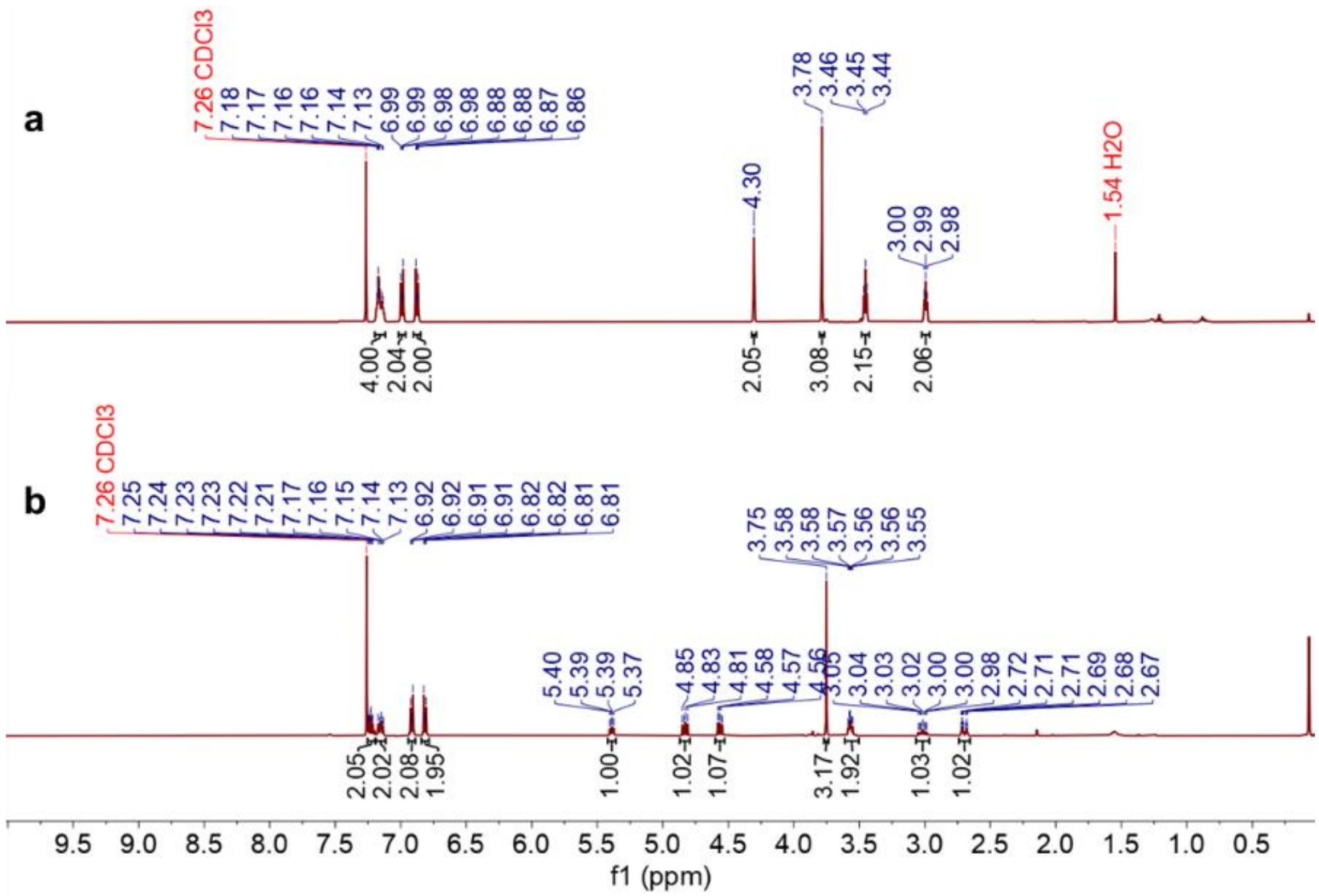

Figure S49. ^{1}H NMR spectra of the reaction mixture in $CDCl_3$ before (**a**) and after (**b**) irradiation from the photocatalytic cross-dehydrogenative coupling (CDC) reaction. Conditions: 50 mM 2-(4-methoxyphenyl)-1,2,3,4-tetrahydroisoquinoline, Ru-bpyap-OMe (1 mol%), CH_3NO_2 (2 mL), O_2 atmosphere, irradiation at 850 nm for 36 h at room temperature.

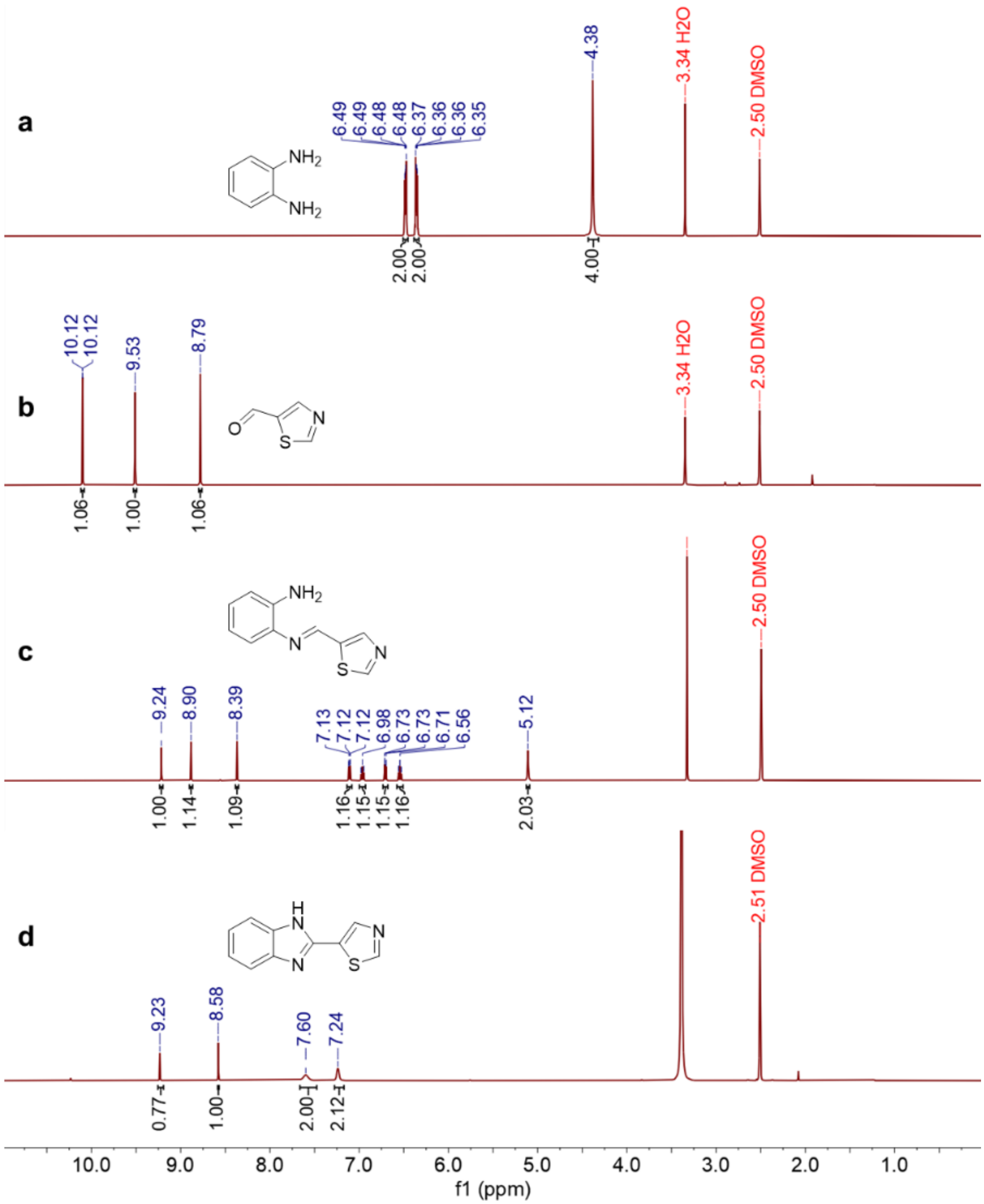

Figure S50. ^{1}H NMR spectra in DMSO-d_6 of (**a**) *o*-phenylenediamine, (**b**) 5-thiazolecarboxaldehyde, the reaction mixture after 15 h (**c**) in dark, and (**d**) under irradiation from the photocatalytic cyclocondensation reaction. Conditions: 50 mM 5-thiazolecarboxaldehyde, *o*-phenylenediamine (1.1 equiv.), Ru-bpyap-OMe (1 mol%), CH_3OH (2 mL), O_2 atmosphere, irradiation at 850 nm for 15 h at room temperature.

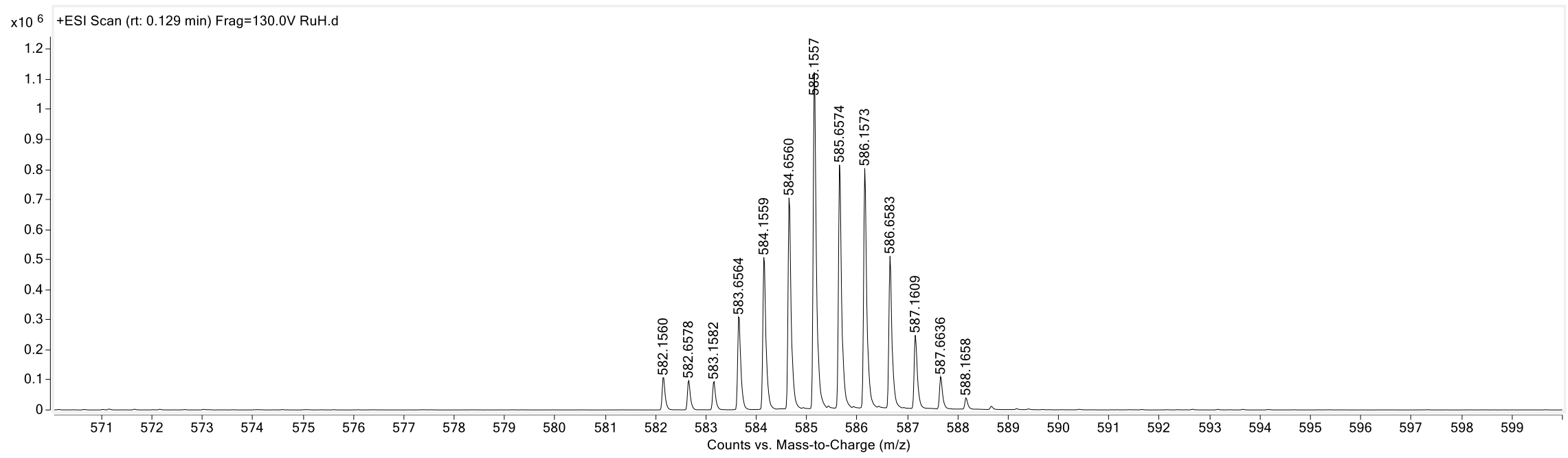

Figure S51. ESI-MS spectrum of **Ru-bpyap**.

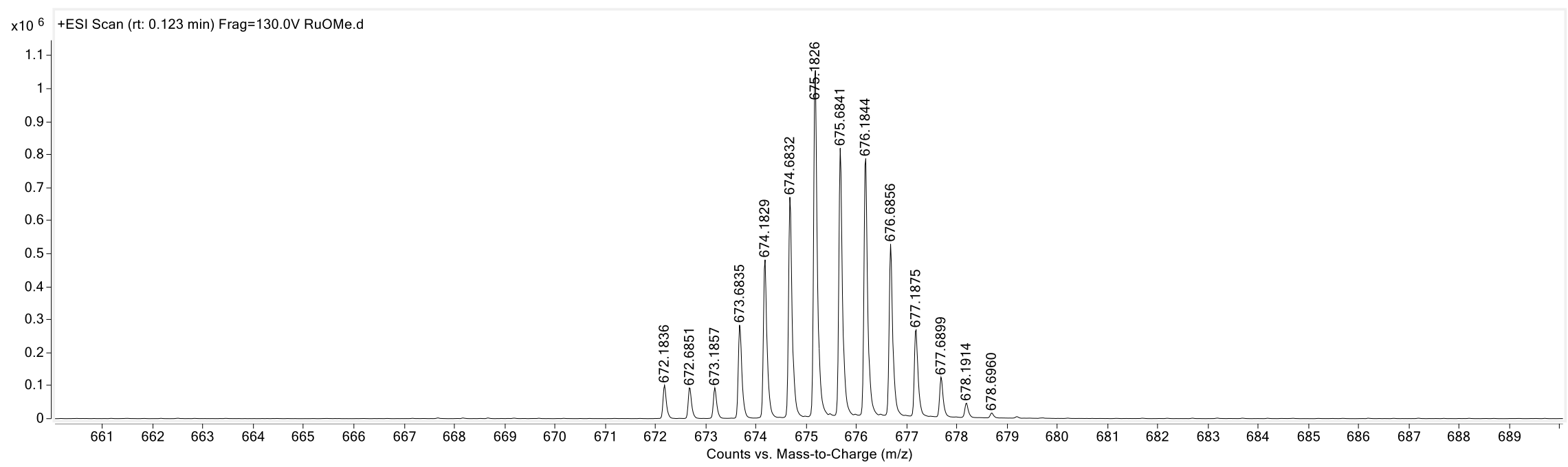

Figure S52. ESI-MS spectrum of **Ru-bpyap-OMe**.

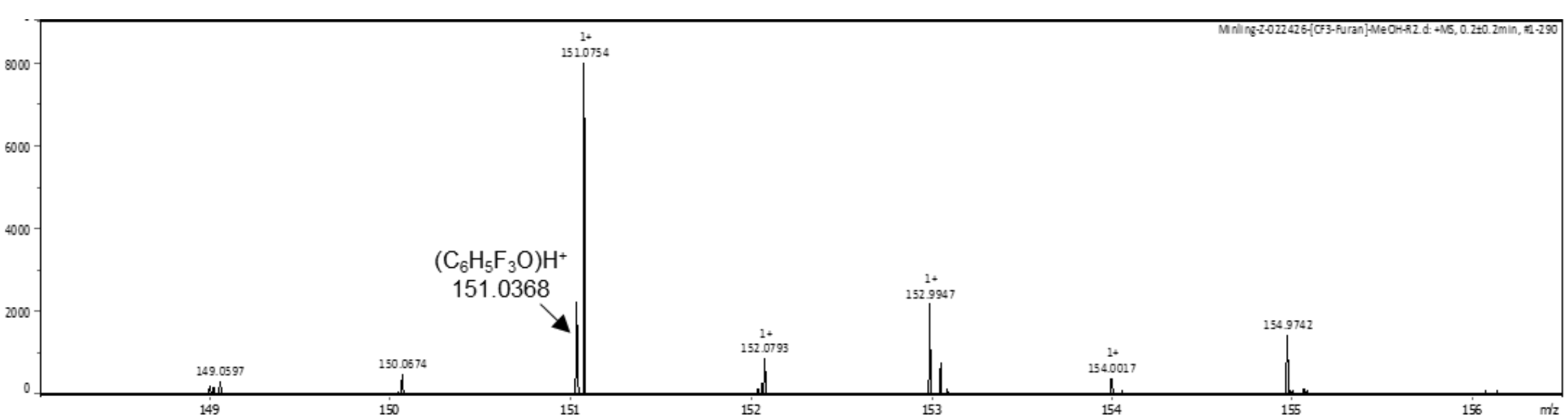

Figure S53. ESI-MS spectra of 5-trifluoromethylation-2-methylfuran.

10. Cartesian coordinates at the optimized geometry

Ru-bpy

C	3.139565	-1.29587	2.771837	H	3.200046	-1.95789	3.627604
C	1.922506	-1.11839	2.124	H	1.026391	-1.6268	2.457153
N	1.783299	-0.30856	1.05823	H	4.977979	0.762728	0.81187
C	2.868652	0.365597	0.58462	H	4.610296	2.024557	-0.81593
C	4.118973	0.226094	1.19333	H	1.64627	3.371254	-3.62524
C	2.616657	1.230073	-0.58519	H	-0.00968	1.926565	-2.45253
C	3.596705	2.017749	-1.19544	H	-1.92257	-0.07506	2.456225
C	1.951402	2.779288	-2.77041	H	-3.29675	-1.79186	3.626423
C	1.019941	1.977235	-2.12122	H	-1.82696	-4.69279	0.813099
N	1.338155	1.218219	-1.05645	H	1.674497	-0.95377	-2.45073
Ru	0.000119	0.000133	0.001027	H	2.09815	-3.1098	-3.62452
C	-1.93046	-1.10533	2.123188	H	-0.55172	-5.00444	-0.81733
C	-2.69268	-2.07057	2.771047	H	0.896891	1.705902	2.454323
C	-1.86293	-3.68022	1.193682	H	0.095886	3.75437	3.623711
C	-1.11734	-2.66708	0.584616	H	-3.1526	3.92753	0.811667
N	-1.15915	-1.38987	1.05776	H	-1.66177	-0.97201	-2.45342
C	-0.24243	-2.88093	-0.58506	H	-3.74143	-0.26198	-3.62621
N	0.386674	-1.76767	-1.05583	H	-4.0604	2.976946	-0.81576
C	1.203492	-1.87104	-2.12027	C	-4.05658	1.423451	-2.29826
C	1.432562	-3.07842	-2.76997	H	-4.96569	1.76852	-2.77827
C	-0.05036	-4.12318	-1.19606	C	-2.65776	-3.38135	2.296662
C	0.007697	2.226654	2.121675	H	-3.23966	-4.16092	2.775839
C	-0.44775	3.369466	2.768982	C	0.793664	-4.22465	-2.29835
C	-2.25738	3.453099	1.192383	H	0.948803	-5.18423	-2.77905
C	-1.75221	2.300699	0.584061	C	4.257518	-0.61106	2.296783
N	-0.62447	1.699548	1.056975	H	5.223688	-0.7254	2.775737
C	-2.37461	1.64918	-0.58522	C	3.263214	2.799325	-2.29797
N	-1.72401	0.548681	-1.05674	H	4.016983	3.413492	-2.77815
C	-2.22136	-0.10631	-2.12196	C	-1.60115	3.99333	2.294708
C	-3.38206	0.298346	-2.77115	H	-1.98586	4.88719	2.773232
C	-3.54709	2.103096	-1.19548				

Ru-bpyap

C	2.988504	1.566595	2.779827	N	-0.09077	1.808052	-1.04843
C	2.076372	0.754576	2.127514	Ru	-0.01267	-0.00153	-6.2E-05
N	1.363346	1.165693	1.060757	C	-0.38234	-2.20602	2.114366
C	1.544599	2.433948	0.594038	C	-0.11968	-3.40078	2.762812
C	2.446019	3.303616	1.200841	C	1.657267	-3.77383	1.179381
C	0.720955	2.797668	-0.57833	C	1.338681	-2.56086	0.575956
C	0.753903	4.050963	-1.18262	N	0.323461	-1.78232	1.047646
C	-0.05981	4.317883	-2.29871	C	2.055094	-2.02003	-0.59852
C	-0.89185	3.281004	-2.76468	N	1.588251	-0.82727	-1.06587
C	-0.87502	2.058514	-2.11505	C	2.184797	-0.26721	-2.13641

C	3.25548	-0.85165	-2.79136	C	-5.78484	-3.34609	-3.48224
C	3.128984	-2.66263	-1.20749	C	-7.60381	2.312568	4.131563
C	-1.71537	1.406908	2.139896	C	-7.60705	3.17152	5.250542
C	-2.87547	1.781424	2.796244	C	-8.82661	1.81027	3.639308
C	-4.10534	0.453453	1.205988	C	-8.81048	3.517349	5.859488
C	-2.90089	0.117286	0.595101	C	-10.0237	2.163076	4.25675
N	-1.71311	0.59392	1.065376	H	-8.82323	1.149534	2.778541
C	-2.8022	-0.76711	-0.58511	H	-8.807	4.178945	6.720156
N	-1.53913	-0.96582	-1.05879	H	-10.9616	1.773481	3.873356
C	-1.36221	-1.76092	-2.13212	C	0.000457	7.95101	-4.10026
C	-2.41056	-2.3875	-2.78412	C	-0.82386	8.20423	-5.21666
C	-3.90238	-1.36597	-1.19176	C	0.845534	8.971605	-3.61643
H	3.535235	1.188078	3.634724	C	-0.79896	9.453054	-5.83183
H	1.895505	-0.25961	2.460662	C	0.861761	10.21647	-4.23998
H	2.584863	4.308254	0.824358	H	1.479236	8.776183	-2.75751
H	1.400603	4.831041	-0.80391	H	-1.43543	9.642228	-6.69066
H	-1.53955	3.430408	-3.61978	H	1.514256	10.99802	-3.86329
H	-1.50003	1.240642	-2.45086	C	5.826813	5.376041	4.127955
H	-1.17699	-1.55245	2.45155	C	6.566679	4.935648	5.245325
H	-0.71587	-3.69464	3.617945	C	6.010646	6.689911	3.64849
H	2.46446	-4.38575	0.799642	C	7.469387	5.795168	5.86572
H	1.776894	0.678524	-2.47032	C	6.916281	7.54037	4.277279
H	3.698316	-0.36065	-3.64918	H	5.441165	7.027959	2.788876
H	3.494321	-3.60834	-0.83016	H	8.035911	5.450705	6.725305
H	-0.74723	1.757243	2.474936	H	7.053638	8.550478	3.904017
H	-2.82283	2.436894	3.656826	C	6.885443	-3.9188	-4.13852
H	-5.04395	0.071694	0.827216	C	7.499857	-3.33095	-5.26405
H	-0.34126	-1.88575	-2.47022	C	7.368249	-5.15056	-3.6491
H	-2.21458	-3.01627	-3.64391	C	8.57334	-3.96609	-5.88276
H	-4.90159	-1.20387	-0.81027	C	8.442507	-5.77625	-4.27622
C	0.926903	-4.21995	2.295886	H	6.895155	-5.60209	-2.78318
C	3.754879	-2.08424	-2.32675	H	9.042103	-3.50902	-6.74869
C	-3.72457	-2.19565	-2.31381	H	8.809753	-6.72414	-3.89525
C	-4.11447	1.300362	2.329224	C	1.824385	-7.74343	4.092653
C	3.191548	2.881435	2.316674	C	1.08543	-8.18053	5.211941
C	-5.33119	1.653688	2.962865	C	2.880011	-8.54135	3.603893
C	-6.37805	1.956172	3.503789	C	1.400409	-9.39029	5.82511
C	-0.03854	5.586969	-2.9278	C	3.185618	-9.74922	4.225432
C	-0.01984	6.67681	-3.46808	H	3.44787	-8.2044	2.742778
C	4.11255	3.752927	2.948302	H	0.828505	-9.72166	6.686276
C	4.902823	4.502096	3.490721	H	3.999192	-10.359	3.844901
C	4.848563	-2.72405	-2.96017	H	0.273414	-7.56639	5.587399
C	5.787802	-3.27457	-3.50309	H	7.127902	-2.38338	-5.63988
C	1.238863	-5.45092	2.923704	H	6.424711	3.924827	5.61353
C	1.509099	-6.50794	3.46194	H	-1.47382	7.418742	-5.58824
C	-4.8326	-2.81429	-2.94331	H	-6.66603	3.557503	5.628682

C	-6.89615	-3.97166	-4.11251	C	9.046528	-5.18742	-5.39196
C	-6.70101	-4.81336	-5.22758	H	9.883861	-5.67899	-5.87783
C	-8.20277	-3.75594	-3.62664	C	7.646307	7.096645	5.384809
C	-7.79296	-5.42359	-5.83905	H	8.351309	7.76332	5.871991
C	-9.28693	-4.37153	-4.24657	C	0.04178	10.46016	-5.34662
H	-8.35199	-3.1083	-2.76885	H	0.057856	11.43239	-5.82967
H	-7.63682	-6.07052	-6.69671	C	-10.0191	3.015583	5.365624
H	-10.2902	-4.20183	-3.86814	H	-10.955	3.288139	5.84379
H	-5.6956	-4.97886	-5.60078	C	-9.08569	-5.20513	-5.35157
C	2.448701	-10.176	5.335005	H	-9.93408	-5.68323	-5.83166
H	2.690746	-11.1186	5.816558				

Ru-bpyap-OMe

C	3.361498	0.367095	2.78438	H	1.675818	-0.93179	2.466041
C	2.21677	-0.05545	2.131396	H	3.993292	3.060517	0.821938
N	1.704614	0.585085	1.061824	H	3.085154	3.978433	-0.80496
C	2.339726	1.697424	0.593575	H	-0.15545	3.743794	-3.63093
C	3.495897	2.177583	1.200645	H	-0.92183	1.694288	-2.45983
C	1.709011	2.334968	-0.5819	H	-1.6541	-0.99448	2.46123
C	2.199243	3.488344	-1.18628	H	-2.03211	-3.15173	3.628779
C	1.54354	4.034185	-2.3062	H	0.649286	-4.99016	0.805618
C	0.39082	3.370179	-2.7735	H	1.924204	-0.03866	-2.45793
C	-0.04185	2.227798	-2.12296	H	3.320468	-1.72157	-3.63159
N	0.592686	1.709103	-1.053	H	1.902118	-4.65598	-0.81756
Ru	-0.00332	0.002412	0.001706	H	-0.04262	1.921113	2.46806
C	-1.16437	-1.89958	2.124029	H	-1.72222	3.322554	3.640514
C	-1.37042	-3.10468	2.772573	H	-4.65372	1.931272	0.810401
C	0.132431	-4.11958	1.186719	H	-0.99957	-1.63425	-2.46588
C	0.294218	-2.87602	0.584067	H	-3.15503	-2.00041	-3.64109
N	-0.35283	-1.7719	1.055538	H	-4.99156	0.678956	-0.81509
C	1.163173	-2.64408	-0.58948	C	-0.71143	-4.26007	2.304829
N	1.178309	-1.36299	-1.05693	C	2.721977	-3.34296	-2.31285
C	1.946435	-1.06865	-2.12462	C	-4.26186	-0.67946	-2.31624
C	2.722193	-2.01136	-2.77644	C	-3.3419	2.738023	2.313619
C	1.919577	-3.64243	-1.19543	C	4.035262	1.515205	2.319884
C	-1.07129	1.950301	2.131035	C	-4.34231	3.516461	2.94137
C	-2.01211	2.728978	2.782157	C	-5.20538	4.185624	3.480585
C	-3.64178	1.940978	1.192559	C	2.028306	5.205567	-2.93368
C	-2.64575	1.180687	0.587792	C	2.447269	6.21422	-3.47269
N	-1.36613	1.18758	1.059561	C	5.209518	1.989521	2.950002
C	-2.87873	0.316146	-0.58883	C	6.220767	2.399116	3.491344
N	-1.77597	-0.33431	-1.05887	C	3.497185	-4.34534	-2.9414
C	-1.90371	-1.14191	-2.13026	C	4.165287	-5.20896	-3.48105
C	-3.10773	-1.34131	-2.78291	C	-0.88643	-5.51647	2.930695
C	-4.12136	0.160712	-1.19532	C	-1.0357	-6.59862	3.469319
H	3.729402	-0.18349	3.641524	C	-5.51738	-0.8487	-2.94537

C	-6.59896	-0.99345	-3.48634	H	-2.57461	-7.13052	5.598316
C	-6.21363	4.964039	4.104352	H	5.770233	-4.89945	-5.60441
C	-5.91514	5.765707	5.223525	H	7.448478	1.326869	5.618074
C	-7.54109	4.951483	3.615439	H	1.391686	7.449998	-5.60363
C	-6.89832	6.532137	5.841533	H	-4.90092	5.784939	5.608968
C	-8.52268	5.711624	4.226296	C	-7.85993	-1.15933	-4.11406
H	-7.7867	4.339015	2.754074	C	-8.0015	-2.00095	-5.23477
C	-8.21183	6.508851	5.344247	C	-9.00271	-0.48224	-3.6273
H	-6.63627	7.137856	6.699728	C	-9.23495	-2.16886	-5.85643
H	-9.54367	5.70932	3.859717	C	-10.2317	-0.64586	-4.24144
C	2.938808	7.387974	-4.09902	H	-8.9097	0.169734	-2.76494
C	2.282893	7.93526	-5.2191	C	-10.3604	-1.48969	-5.361
C	4.099183	8.03398	-3.6116	H	-9.31	-2.82281	-6.71607
C	2.758696	9.086242	-5.83948	H	-11.1144	-0.13146	-3.87654
C	4.576656	9.178777	-4.2247	H	-7.13311	-2.52633	-5.61879
H	4.615648	7.624554	-2.7496	O	-9.24544	7.212821	5.865531
C	3.912265	9.716047	-5.34361	C	-9.00525	8.04359	7.004364
H	2.231373	9.481309	-6.69858	H	-8.2722	8.82634	6.778392
H	5.465503	9.681967	-3.85922	H	-8.65864	7.454281	7.860944
C	7.399449	2.878569	4.117796	H	-9.96411	8.503059	7.244483
C	7.941482	2.21475	5.235726	O	4.463211	10.83776	-5.86705
C	8.055473	4.034601	3.633268	O	10.86353	4.366616	5.888683
C	9.096924	2.678644	5.85676	O	7.190065	-9.24584	-5.87432
C	9.204637	4.500394	4.247262	O	-1.63673	-11.6058	5.865526
H	7.650166	4.557097	2.77296	O	-11.6063	-1.57779	-5.88599
C	9.736584	3.827957	5.363915	C	3.842835	11.44083	-7.0056
H	9.488035	2.145703	6.714193	H	2.819323	11.76023	-6.77855
H	9.715499	5.385804	3.884111	H	3.830791	10.75647	-7.8615
C	4.942463	-6.21654	-4.10744	C	11.46121	3.735314	7.024053
C	5.747876	-5.9152	-5.22315	H	11.77066	2.709844	6.792173
C	4.925514	-7.54601	-3.62412	H	10.77716	3.725829	7.880268
C	6.514013	-6.89745	-5.843	C	8.02653	-9.00222	-7.00824
C	5.685588	-8.52663	-4.23663	H	8.80948	-8.27145	-6.77556
H	4.310104	-7.79379	-2.76548	H	7.441854	-8.65093	-7.86605
C	6.486754	-8.21284	-5.35093	C	-11.813	-2.41537	-7.02634
H	7.122454	-6.63322	-6.69861	H	-11.572	-3.46058	-6.80145
H	5.680248	-9.54909	-3.87419	H	-11.2155	-2.0781	-7.881
C	-1.20643	-7.85963	4.095667	C	-2.47824	-11.8108	7.003343
C	-2.05041	-7.99984	5.214756	H	-3.52222	-11.5676	6.775145
C	-0.53113	-9.0038	3.609586	H	-2.14255	-11.2142	7.859154
C	-2.22202	-9.23321	5.835521	H	4.449788	12.31338	-7.24736
C	-0.69825	-10.2327	4.223079	H	-12.8724	-2.33143	-7.26867
H	0.122552	-8.91182	2.748407	H	12.33963	4.332834	7.268083
C	-1.54439	-10.3599	5.341068	H	8.485496	-9.96083	-7.25029
H	-2.87746	-9.30748	6.693986	H	-2.39731	-12.8705	7.245823
H	-0.18504	-11.1162	3.858646				

11. Reference

(1) Houk, A.L.; Givens, R. S.; Elles, C.G. Two-Photon Activation of p-Hydroxyphenacyl Phototriggers: Toward Spatially Controlled Release of Diethyl Phosphate and ATP. *J. Phys. Chem. B* **2016**, *120* (16), 3178–3186.

(2) Srivastava, P.; Stierwalt, D. A.; Elles, C.G. Broadband Two-Photon Absorption Spectroscopy with Stimulated Raman Scattering as an Internal Standard. *Anal. Chem.* **2023**, *95* (35), 13227–13234.

(3) Frisch, M. J.; Trucks, G. W.; Schlegel, H. B.; Scuseria, G. E.; Robb, M. A.; Cheeseman, J. R.; Scalmani, G.; Barone, V.; Petersson, G. A.; Nakatsuji, H.; Li, X.; Caricato, M.; Marenich, A. V.; Bloino, J.; Janesko, B. G.; Gomperts, R.; Mennucci, B.; Hratchian, H. P.; Ortiz, J. V.; Izmaylov, A. F.; Sonnenberg, J. L.; Williams; Ding, F.; Lipparini, F.; Egidi, F.; Goings, J.; Peng, B.; Petrone, A.; Henderson, T.; Ranasinghe, D.; Zakrzewski, V. G.; Gao, J.; Rega, N.; Zheng, G.; Liang, W.; Hada, M.; Ehara, M.; Toyota, K.; Fukuda, R.; Hasegawa, J.; Ishida, M.; Nakajima, T.; Honda, Y.; Kitao, O.; Nakai, H.; Vreven, T.; Throssell, K.; Montgomery Jr., J. A.; Peralta, J. E.; Ogliaro, F.; Bearpark, M. J.; Heyd, J. J.; Brothers, E. N.; Kudin, K. N.; Staroverov, V. N.; Keith, T. A.; Kobayashi, R.; Normand, J.; Raghavachari, K.; Rendell, A. P.; Burant, J. C.; Iyengar, S. S.; Tomasi, J.; Cossi, M.; Millam, J. M.; Klene, M.; Adamo, C.; Cammi, R.; Ochterski, J. W.; Martin, R. L.; Morokuma, K.; Farkas, O.; Foresman, J. B.; Fox, D. J. Wallingford, CT, 2016.

(4) Becke, A. D. Density-functional exchange-energy approximation with correct asymptotic-behavior. *Phys. Rev. A* **1988**, *38* (6), 3098.

(5) Becke, A. Density-functional thermochemistry. III. The role of exact exchange. *J. Chem. Phys.* **1993**, *98* (7), 5648–5652.

(6) Lee, C.; Yang, W.; Parr, R. G. Development of the Colle-Salvetti correlation-energy formula into a functional of the electron density. *Phys. Rev. B* **1988**, *37* (2), 785.

(7) Hay, P. J.; Wadt, W. R. Ab initio effective core potentials for molecular calculations. Potentials for the transition metal atoms Sc to Hg. *J. Chem. Phys.* **1985**, *82* (1), 270–283.

(8) Krishnan, R.; Binkley J. S.; Binkley, J. S.; Seeger, R.; Pople, J. A. Self-consistent molecular orbital methods. XX. A basis set for correlated wave functions. *J. Chem. Phys.* **1980**, *72* (1), 650–654.

(9) Hariharan, P. C.; Pople, J. A. The influence of polarization functions on molecular orbital hydrogenation energies. *Theor. Chim. Acta* **1973**, *28* (3), 213–222.

(10) Francl, M. M.; Pietro, W. J.; Hehre, W. J.; Binkley, J. S.; Gordon, M. S.; DeFrees, D. J.; Pople, J. A. Self-consistent molecular orbital methods. XXIII. A polarization-type basis set for second-row elements. *J. Chem. Phys.* **1982**, *77* (7), 3654–3665.

(11) Clark, T.; Chandrasekhar, J.; Spitznagel, G. W.; Schleyer, P. V. R. Efficient diffuse function-augmented basis sets for anion calculations. III. The 3-21+ G basis set for first-row elements, Li–F. *J. Comput. Chem.* **1983**, *4* (3), 294–301.

(12) O'boyle, N. M.; Tenderholt, A. L.; Langner, K. M. Cclib: a library for package-independent computational chemistry algorithms. *J. Comput. Chem.* **2008**, *29* (5), 839–845.

(13) Humphrey, W.; Dalke, A.; Schulten, K. VMD: visual molecular dynamics. *J. Mol. Graph.* **1996**, *14* (1), 33–38.
